# Tailoring the AIE Chromogen 2-(2-Hydroxyphenyl)benzothiazole for Use in Enzyme-Triggered Molecular Brachytherapy

**DOI:** 10.3390/molecules27248682

**Published:** 2022-12-08

**Authors:** Zhiyuan Wu, Jinghuai Dou, Kathy-Uyen Nguyen, Jayden C. Eppley, Kittipan Siwawannapong, Yunlong Zhang, Jonathan S. Lindsey

**Affiliations:** Department of Chemistry, North Carolina State University, Raleigh, NC 27695, USA

**Keywords:** aggregation, cancer, insolubilization, molecular design, phosphodiester, precipitation, radiotherapy

## Abstract

A targeted strategy for treating cancer is antibody-directed enzyme prodrug therapy, where the enzyme attached to the antibody causes conversion of an inactive small-molecule prodrug into an active drug. A limitation may be the diffusion of the active drug away from the antibody target site. A related strategy with radiotherapeutics entails enzymatically promoted conversion of a soluble to insoluble radiotherapeutic agent, thereby immobilizing the latter at the target site. Such a molecular brachytherapy has been scarcely investigated. In distinct research, the advent of molecular designs for aggregation-induced emission (AIE) suggests translational use in molecular brachytherapy. Here, several 2-(2-hydroxyphenyl)benzothiazole substrates that readily aggregate in aqueous solution (and afford AIE) were elaborated in this regard. In particular, (1) the 2-(2-hydroxyphenyl) unit was derivatized to bear a pegylated phosphodiester that imparts water solubility yet undergoes enzymatic cleavage, and (2) a *p*-phenol unit was attached to the benzo moiety to provide a reactive site for final-step iodination (here examined with natural abundance iodide). The pegylated phosphodiester-iodinated benzothiazole undergoes conversion from aqueous-soluble to aqueous-insoluble upon treatment with a phosphatase or phosphodiesterase. The aggregation is essential to molecular brachytherapy, whereas the induced emission of AIE is not essential but provides a convenient basis for research development. Altogether, 21 compounds were synthesized (18 new, 3 known via new routes). Taken together, blending biomedical strategies of enzyme prodrug therapy with materials chemistry concerning substances that undergo AIE may comprise a step forward on the long road toward molecular brachytherapy.

## 1. Introduction

Brachytherapy is a medical treatment where radionuclide-bearing macroscopic objects are implanted in a tumor. The term “brachy” (Gk, n. β*ρ*αχυ′), meaning “short” or “few”, refers to the proximal location of the radionuclide-bearing seeds (typically pins or beads) to the diseased tissue [1,2]. While successful for the treatment of localized solid tumor cancers, brachytherapy has not been adapted for treatment of metastatic cancer given the challenge of identification and surgical implantation in metastases. At a pole apart from surgical implantation of macroscopic objects are various strategies for molecular localization of therapeutic agents at or near the tumor site. Such methods include antibody-directed enzyme prodrug therapy (ADEPT) [3,4,5,6], pretargeted radioimmunotherapy (PRIT) [7,8], enzyme-instructed self-assembly (EISA) [9], and enzyme-mediated cancer imaging and therapy (EMCIT) [10,11,12,13,14,15,16,17]. Each has pros and cons.

ADEPT and PRIT rely on antibodies for targeted delivery of a therapeutic agent. ADEPT employs an antibody–enzyme conjugate that following localization at the target site causes conversion of a subsequently administered prodrug to a drug. The target-site localization can be thwarted by the diffusibility of the released drug, and chemotherapy is known to be limited in general given the intrinsic heterogeneity of tumor tissue and the Darwinian nature of cancer [18]. On the other hand, radiation can eradicate a heterogeneous cellular population within a tumor [19]. PRIT employs an antibody bearing a conjugatable motif rather than an enzyme; following localization at the target site, administration of a radionuclide-bearing molecule equipped with a complementary conjugatable motif results in covalent attachment to the antibody. The quantity of radionuclide that can be delivered appears to be comparable with the quantity of antibody.

**Figure 1:**
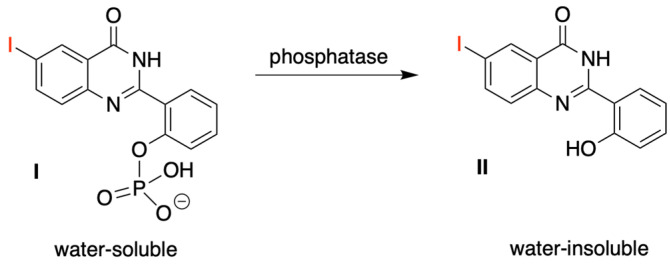
Quinazolinyl–phosphoester cleavage.

EMCIT and EISA rely on the presence of up-regulated quantities of endogenous enzyme in the tumor for localization of a therapeutic agent. In one example of EISA [20], a nanoparticle bearing amphiphilic peptides undergoes peptidolysis by extracellular matrix metalloproteinases, causing circulating nanoparticles to aggregate in the tumor extracellular space. In EMCIT, a radionuclide-containing agent equipped with an enzymatically cleavable water-solubilizing group is converted to a water-insoluble precipitate by enzymes in the tumor. The chief structure investigated has been a water-soluble radioiodinated quinazolinyl–phosphoester (**I**), which upon treatment with alkaline phosphatase affords the water-insoluble counterpart (**II**) [10,12,13,17] (Figure 1). Substantial difficulty was encountered in the synthesis of the quinazolinyl–phosphoester (**I**), which was found to exist as a mixture of two compounds, only one of which was hydrolyzable by alkaline phosphatase (the other is not shown in Figure 1) [12]. A porphyrin–phosphoester also could be used [21]. This approach requires the presence of an endogenous enzyme that is more abundant in the tumor than in normal cells, although melding with an ADEPT procedure, where the enzyme is delivered by targeting, has been proposed [22]. More elaborate therapeutic strategies also have been proposed that encompass the EMCIT process [23,24,25,26,27].

The successful use of enzyme-mediated localization of a therapeutic agent requires substantial excess of the enzyme in diseased versus normal tissue. One approach is to use a non-native (i.e., exogenous) enzyme rather than rely on differential expression of an endogenous enzyme. Such a strategy has been employed in the earliest instantiations of the ADEPT approach [28] but, to our knowledge, has not been employed with EMCIT. The development of targeted strategies requires focus on multiple, rather independent components. Recently, we engineered a non-native, archaeal phosphodiesterase to trigger the cleavage of a benzothiazole–phosphodiester to release the benzothiazole under conditions wherein a phosphatase was comparatively inactive [29]. In this paper, we focus on chemistry of the benzothiazole unit. The molecular design and synthesis of the benzothiazole–phosphodiester are extended to accommodate iodination as required for use in brachytherapy. Here, cold iodide is employed as a model study but under conditions identical to those for use with radioiodide. In short, the research reported here focuses on the “A” part of AIE, which is essential for molecular brachytherapy, whereas the “IE” is described as a convenient marker during developmental work.

## 2. Reconnaissance

**Figure 2:**
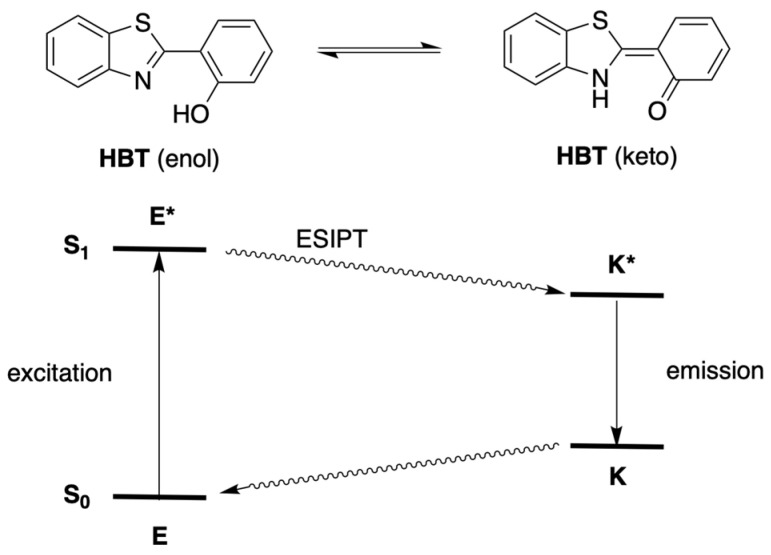
ESIPT process of **HBT** causing conversion between enol and keto forms.

The compound 2-(2-hydroxyphenyl)benzothiazole (**HBT**) is well-known for its solid-state fluorescence, more commonly referred to as AIE [30], and thus may be a suitable candidate for radiopharmaceutical development. The intermolecular hydrogen bonding rigidifies the molecular conformation of **HBT** and restricts intramolecular rotations, which underpins the AIE effect [31]. In addition to restriction of intramolecular rotations, hydrogen bonding also facilitates an excited-state intramolecular proton transfer (ESIPT) process between enol/keto forms and thus generates solid-state emission with a large Stokes shift (Figure 2) [30,32,33].

**Figure 3:**
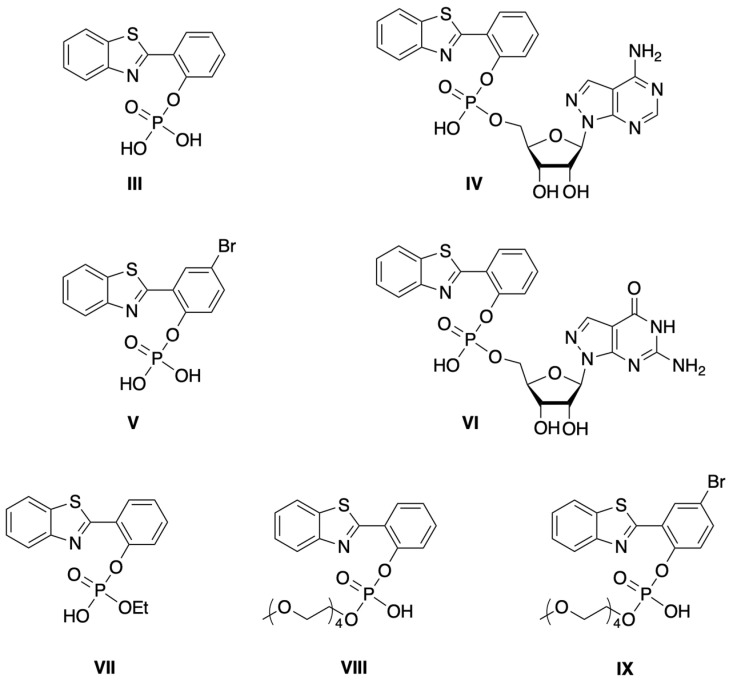
Reported HBT probes **III**−**IX**.

**Figure 4:**
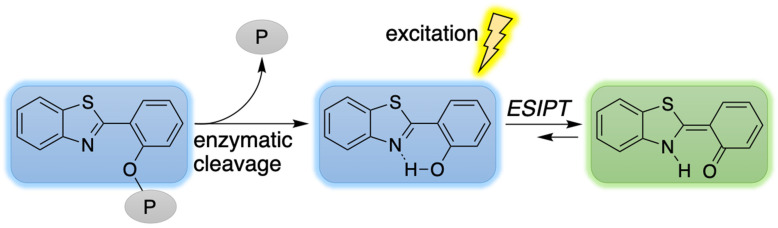
Proposed strategy for immobilizing **HBT** upon enzymatic cleavage.

Blocking the hydroxy group by phosphorylation, as shown for **III**–**IX**, can trap the HBT unit in the enol form and prohibit the ESIPT process. (Figure 3) [29,32,34,35]. Enzymatic cleavage of the phosphoryl group unveils the hydroxy group, and the HBT unit can undergo ESIPT with accompanying emission, typically in the green region (Figure 4).

**Scheme 1:**
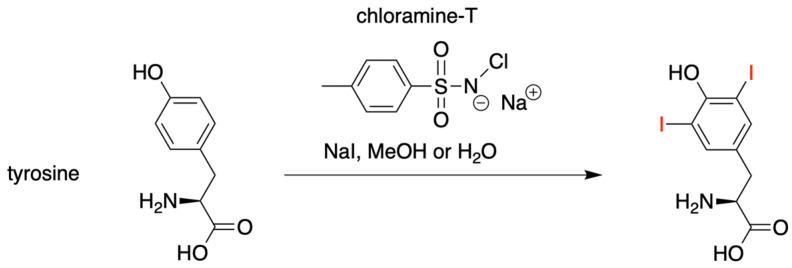
Common method for iodination of tyrosine.

To exploit the aggregation phenomenon in a molecular brachytherapy strategy, the HBT substrate needs to be radiolabeled. Radiolabeling generally is done at the last step of the synthesis to limit challenges in purification and handling. Moreover, the radiolabeling must be achieved without altering the soluble-insoluble conversion process, both in terms of enzymatic specificity and solubility features. Here, we explored several HBT derivatives and their amenability toward radiolabeling. A new route to introduce radioactive iodine relies on the copper-mediated conversion of a boronic ester [36]; however, we were unable to prepare the required boronate of the HBT derivative. A more traditional chemistry employs *N*-chloro-*p*-toluenesulfonamide (chloramine-T) for iodination of substrates such as the phenol of tyrosine, which has been widely used in labeling proteins (Scheme 1) [37,38,39]. Here, we focused on the installation of natural abundance iodine as a mock (cold) version of ^131^I.

## 3. Results and Discussion

### 3.1. Synthesis

#### 3.1.1. HBT Derivatives for Studies of Iodination

**Scheme 2:**
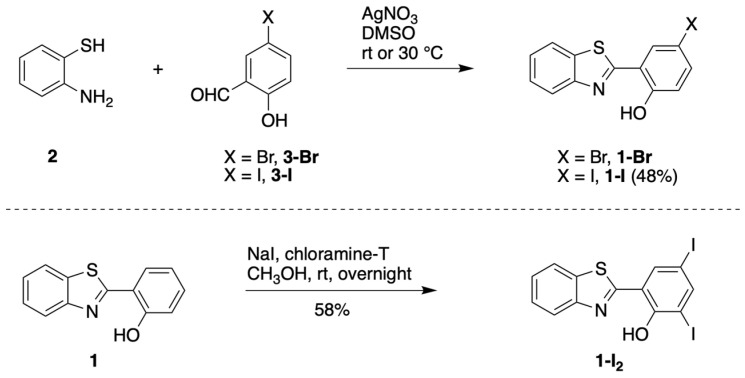
Synthesis of halogen-substituted HBT derivatives.

We first synthesized several model compounds for assessing the installation of iodide. Compound **1-Br** was synthesized by reaction of 2-aminobenzenethiol (**2**) and 5-bromosalicylaldehyde (**3-Br**) following a reported method [40] using AgNO_3_ in dimethylsulfoxide (DMSO) (Scheme 2). A similar reaction from 5-iodosalicylaldehyde (**3-I**) gave **1-I** in 48% yield. The derivative **1-I** was also reported to be accessible from **1** by reaction with NaI in the presence of chloramine-T [41]. In our hands, the occurrence of iodination at one or both *o*- and *p*-sites resulted in a mixture that proved difficult to separate. For simplification, treatment of **1** with excess NaI provided the known [42] diiodinated product **1-I_2_** in 58% yield.

**Scheme 3:**
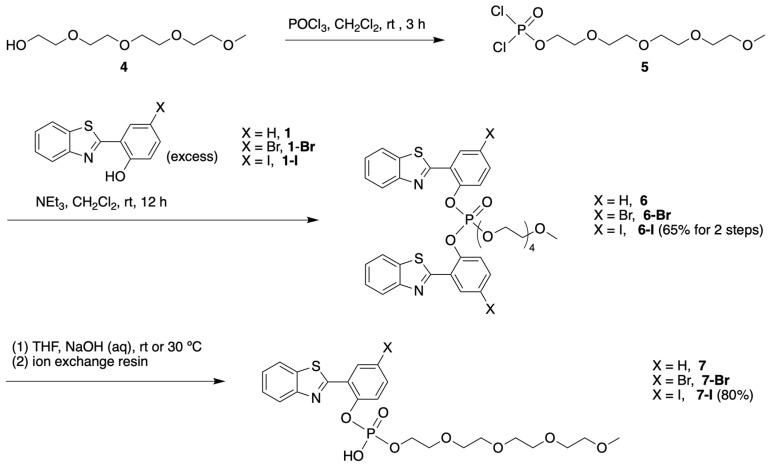
Synthesis of phosphoester derivatives of HBT.

The next design feature entailed installation of a water-soluble phosphodiester unit. The reaction of the short polyethylene glycol (PEG) chain **4** with POCl_3_ gave the dichlorophosphoester derivative **5** (Scheme 3) [29]. The reaction of crude **5** with excess **1-I** in the presence of triethylamine gave phosphotriester **6-I** in 65% yield (two steps). Subsequent treatment with 5% aqueous NaOH solution in tetrahydrofuran (THF) caused removal of one HBT unit to give the target phosphodiester **7-I** in 80% yield.

#### 3.1.2. Introduction of Iodine with HBT Substrates

**Scheme 4:**
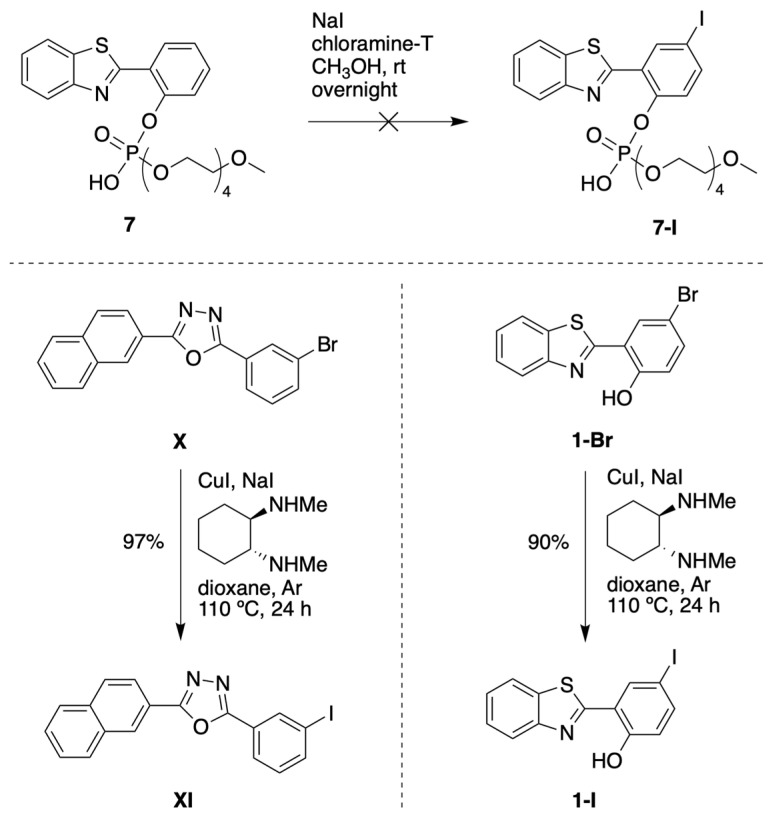
Halogen replacement with HBT derivatives.

Treatment of **1** with NaI and chloramine-T gave an acceptable yield of the diiodinated product, as described above. On the other hand, treatment of phosphodiester **7** to the same conditions did not afford iodination (Scheme 4, top panel). The apparent deactivation by the phosphoester moiety thus precludes use of direct radioiodination at the final step of the synthesis.

A reported aromatic Finkelstein-like reaction [43] (**X** → **XI**) prompted analogous conversion of **1-Br** to **1-I** (Scheme 4). We found the conversion of **1-Br** to **1-I** to be <90% on the basis of ^1^H NMR spectroscopy. Our concerns were as follows: (1) the separation of **1-I** in the presence of unreacted **1-Br** is difficult; (2) the condition for iodination is also considered to be too harsh for the reaction of the analogous phosphodiester substrate **7-Br**; (3) the reaction time is longer than other iodination methods; and (4) the required concentrations of both **1-Br** and NaI are quite high. The implications of the latter requirement are subtle: because only tiny quantities of a radioiodide source are employed, achieving a high concentration requires doping with a large quantity of cold iodide, thereby diluting the radiochemical yield.

#### 3.1.3. HBT Derivatives with an Appended Aryloxy Group for Iodination

**Scheme 5:**
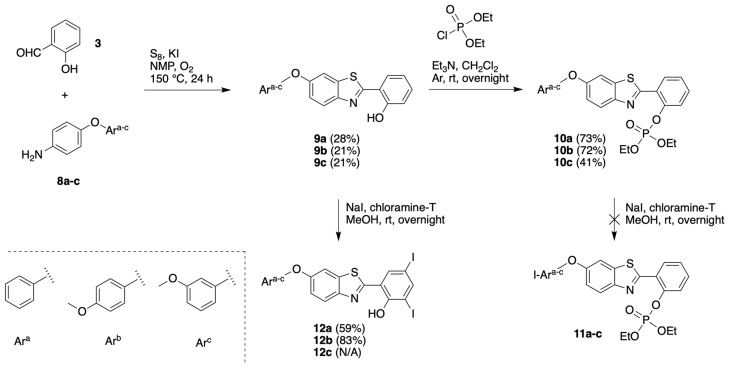
Synthesis of HBT derivatives with an appended aryloxy group.

As several iodination methods examined above were unsuccessful, a new set of derivatives **9a-c** was proposed and synthesized following a known procedure [44] (Scheme 5). The synthesis entailed reaction of salicylaldehyde (**3**) and a 4-(aryloxy)aniline (**8a-c**) at elevated temperature in *N*-methyl-2-pyrrolidone (NMP) in the presence of elemental sulfur and KI under air. The new derivatives were designed to overcome the following issues: (1) the phosphodiester analogues do not readily undergo iodination or halogen conversion on the 2-phenolic ring; and (2) the halogen-substituted phosphodiesters (**7-Br** and **7-I**) in either water or phosphate buffer solution (pH 7.0) were found to undergo spontaneous self-cleavage to some extent [29]. We expected that an appended aryloxy ring on the benzo unit of the benzothiazole would provide a distinct reaction site for iodination under the classic chloramine-T condition. Moreover, the appended aryloxy group could be rendered more reactive by introducing an additional methoxy group. This new iodination site might also provide higher stability of the phosphodiesters.

Treatment of **9a–c** with diethyl chlorophosphate gave the respective phosphotriesters **10a–c**. Reaction of the triesters with excess NaI and chloramine-T led to no corresponding iodinated compounds (**11a–c**); instead, formation of **12a–c** was observed due to in situ dephosphorylation. To further confirm the reactivity of the appended aryloxy group, reaction of non-protected **9a–c** with NaI and chloramine-T under the same condition gave **12a–c** as the major products, where the iodination proceeded exclusively on the 2-phenol rather than on the appended aryloxy group. Hence, this design was not satisfactory.

#### 3.1.4. HBT Derivatives with an Appended Phenol Group for Iodination

**Scheme 6:**
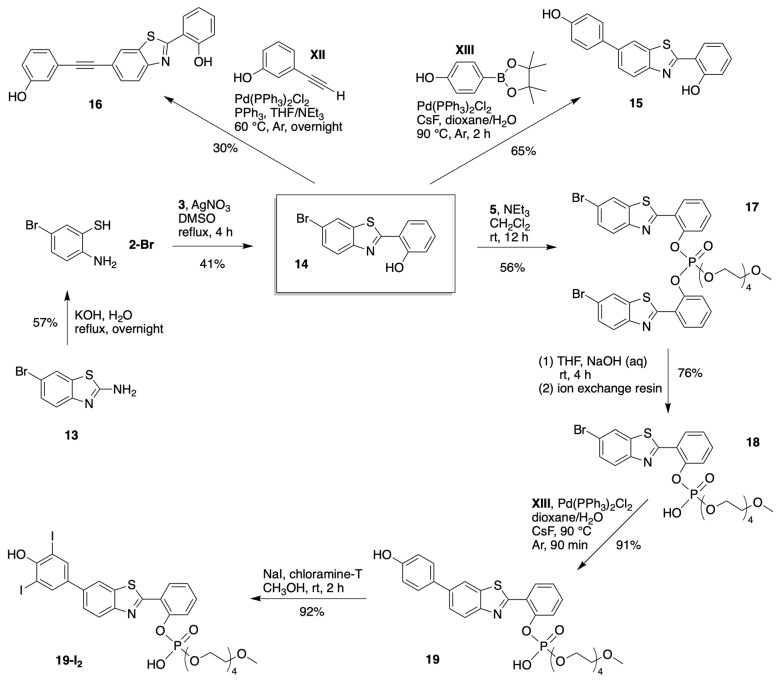
Installation of appended phenols on HBT derivatives.

In an alternative design, we sought to append a phenol to the benzo unit of the benzothiazole. Known compound **2-Br** [45] was converted to the new derivative 6-bromo-2-(2-hydroxyphenyl)-1,3-benzothiazole (**14**) in 40% yield (Scheme 6). We first examined whether **14**, which lacks a PEG-phosphodiester group, is stable toward Suzuki or Sonogashira coupling conditions. In so doing, reaction of **14** with 3-ethynylphenol (**XII**) or 4-(4,4,5,5-tetramethyl-1,3,2-dioxaborolan-2-yl)phenol (**XIII**) gave **15** or **16** in yield of 30% or 65%, respectively. We then subjected **14** to the established two-step procedure (as shown in Scheme 3) for installation of the PEG-phosphodiester. In this manner, intermediate **17** was formed and then hydrolyzed to give the desired phosphodiester **18** in an overall yield of 43%. Compound **18** was then treated to the Suzuki coupling condition with **XIII** to give in 91% yield the HBT derivative bearing an appended phenol on the benzo unit and a PEG-phosphodiester on the 2-phenol unit (**19**). The reaction of **19** with NaI and chloramine-T for 2 h consumed all starting material and gave the desired diiodinated product **19-I_2_**. ^1^H NMR spectroscopic examination of **19-I_2_** showed the disappearance of the resonances of the protons *ortho* to the hydroxy group and the upfield shifts of peaks attributed to the two remaining aromatic protons (*meta* to the hydroxy group), consistent with formation of the desired diiodinated product.

### 3.2. Chemical Characterization

**Figure 5:**
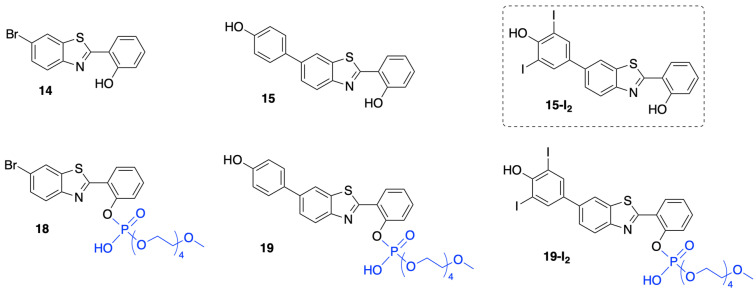
Structures of HBT derivatives and phosphodiester counterparts.

Each new compound was characterized by ^1^H and proton-decoupled ^13^C NMR spectroscopy as well as by electrospray ionization mass spectrometry (ESI-MS) for accurate mass analysis. The NMR spectra for all new compounds are provided as Supplementary Materials. Solid compounds also were subjected to melting point determinations. Each phosphodiester derivative also was characterized by ^31^P NMR spectroscopy. A single ^31^P resonance was observed in each case with chemical shift (in ppm relative to H_3_PO_4_ as an external standard) of −13.40 (**6-I**) and −13.12 (**17**) for the diarylphosphotriesters; −6.94 (**10a**), −6.95 (**10b**), and −6.95 (**10c**) for the monoarylphosphotriesters; and −6.61 (**7-I**), −6.61 (**18**), −5.62 (**19**), and −5.68 (**19-I_2_**) for the monoarylphosphodiesters. Absorption and emission spectra were collected of selected synthetic compounds (**14**, **15**, **18**, **19**, and **19-I_2_**) as described below. Compounds **14**, **15**, and **15-I_2_** (not synthesized) are the respective products from **18**, **19**, and **19-I_2_** after enzymatic cleavage (Figure 5).

### 3.3. Absorption and Emission Properties

#### 3.3.1. Homogeneous Solution

**Figure 6:**
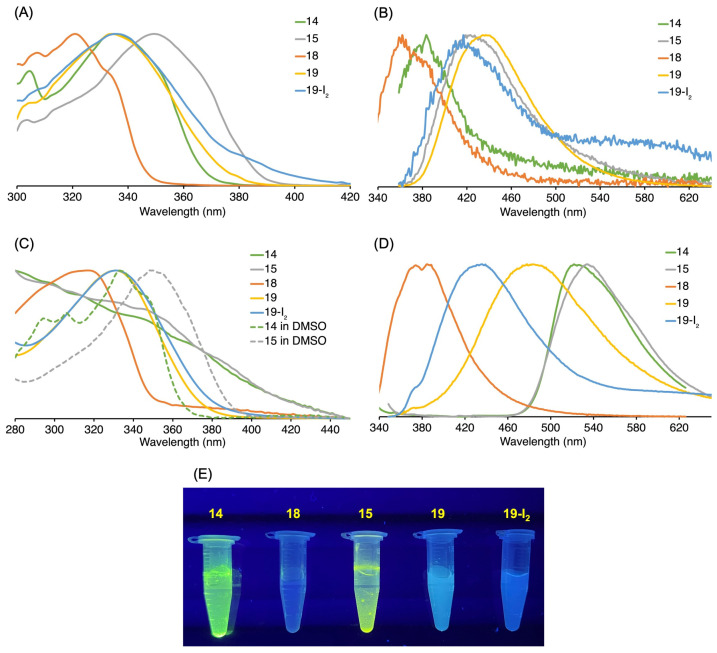
(**A**) Absorption spectra (normalized) at room temperature of **14**, **15**, **18**, **19**, and **19-I_2_** in ethanol. (**B**) Emission spectra (normalized) at room temperature of **14**, **15**, **18**, **19**, and **19-I_2_** in ethanol. Spectra were collected with a 2 nm bandpass, and 4 spectra were averaged to generate the display shown. (**C**) Absorption spectra (normalized) at room temperature of **14**, **15**, **18**, **19**, and **19-I_2_** (20 μM) in H_2_O containing 0.1% DMSO, as well as **14** and **15** (20 μM) in 100% DMSO. (**D**) Emission spectra (normalized) at room temperature of **14**, **15**, **18**, **19**, and **19-I_2_** (20 μM) in H_2_O containing 0.1% DMSO. Spectra were collected with a 5 nm bandpass, and 20 spectra were averaged to generate the display shown. (**E**) Visualization (UV illumination) of **14**, **18**, **15**, **19**, and **19-I_2_** (20 μM) in H_2_O containing 0.1% DMSO at room temperature.

The absorption spectra of **14**, **15**, **18**, **19**, and **19-I_2_** in ethanol are shown in Figure 6A (vide infra). The absorption peak maxima range from 321 to 349 nm. The fluorescence spectra are shown in Figure 6B. The emission peak maxima range from 361 to 436 nm. The fluorescence quantum yield (Φ_f_) values were determined using quinine sulfate as a standard, which exhibits spectral maxima (λ_abs_ 347 nm, λ_em_ 446 nm) and bands that are conveniently similar to those of the five compounds examined here. The absorption, fluorescence, and Φ_f_ values are provided in Table 1. The Φ_f_ values range from 0.017 to 0.64.

Understanding of such values requires context. First, the Φ_f_ of the core compound 2-phenylbenzothiazole (lacking the 2-hydroxy group of the HBT molecules) in *n*-heptane at room temperature is 0.005 [46]. Second, the Φ_f_ of 2-(2-hydroxyphenyl)benzothiazole (**HBT** or **1**) in a mixed solvent of methylcyclohexane and 2-methylbutane at 298 K is 0.011 [47], 0.01 in CH_2_Cl_2_ [48], and 0.0014 in acetonitrile [49]. Third, the Φ_f_ of **1** increases by ~30-fold upon decrease in temperature to 96 K [47]. Fourth, for **1** in the polar solvent neat ethanol at 298 K, the Φ_f_ of the enol form was 0.019, and the keto form was 0.002, giving a total value of 0.021 [47]. The values increased to 0.128 and 0.033, respectively, at 96 K [47]. The spectral features of 2-(2-hydroxyphenyl)benzothiazole (**1**) are known to be sensitive to the installation of substituents [48,50], as are those of 2-phenylbenzothiazole [51].

**Table 1:** Absorption and emission properties of compounds in ethanol.

Compound	λ_abs_ (nm)	λ_exc_ (nm)	λ_em_ (nm) *^b^*	λ_f_ *^c^*
**14**	335	347	383	0.017
**15**	349	347	422	0.066
**18**	321	321*^a^*	361	0.026
**19**	334	347	436	0.64
**19-I_2_**	336	347	416	0.027

*^a^* No obvious absorbance for **18** was observed at 347 nm. *^b^* Excitation band pass 2 nm; emission band pass 2 nm. *^c^* The fluorescence quantum yields were determined relative to quinine sulfate in 0.05 M sulfuric acid (Φ_f_ = 0.55) [47,52] with application of the refractive index correction factor *n_sample_*^2^/*n_standard_*^2^ = 1.046 (*n_sample_*: refractive index of the sample (ethanol 1.36), *n_standard_*: refractive index of the standard (0.05 M H_2_SO_4_, 1.33)).

Some comments about the relationship of structure and Φ_f_ values can be proffered. Compound **14**, which is a bromo derivative of **1**, exhibits a Φ_f_ value similar to that of **1**. Compound **18**, the PEG-ylated analogue of **14**, exhibits a Φ_f_ value similar to that of **14**. Compound **15**, the *p*-phenol-substituted analogue of **1**, exhibits a Φ_f_ value of 0.066, approximately three times that of **1**. Upon PEG-phosphorylation of **15** to create **19**, the Φ_f_ value increases by ~10-fold, to 0.64. Finally, installation of two iodo atoms (**19-I_2_**) to **19** results in a low Φ_f_ value (0.027). The generally low values of Φ_f_ cohere with the literature reports for similar compounds, as does the high value for **19** given the known effects of substituents on the HBT chromophore. If required to speculate, it may be that the *p*-phenol substituent at the 5-position enhances the fluorescence, whereas the 2-hydroxyphenyl (or even the phenyl) group at the 2-position suppresses fluorescence; the PEG-phosphoryl group causes steric hindrance and shuts off the suppressive effect of the 2-aryl group giving the high value observed for **19**. The low value of **19-I_2_** would then stem from a combination of lessened electron-releasing effect of the *p*-hydroxy group and the heavy atom effect caused by the two iodine atoms. Certainly, additional compounds and time-resolved studies are essential for in-depth understanding of the limited results for the compounds examined here in homogeneous solution.

#### 3.3.2. Heterogeneous Samples

Our emphasis is to tailor the HBT compound for use in molecular brachytherapy. Here, we relied on the induced emission of the synthetic HBT substrates as a signal to demarcate the presence of aggregation derived from compounds **18**, **19**, and **19-I_2_** upon hydrolysis by the action of a phosphodiesterase, thereby liberating the corresponding compounds lacking the PEG-phosphodiester moiety. The complexity of the aforementioned solution spectral features of the compounds **14**, **15**, **18**, **19**, and **19-I_2_** described above do not augur well for understanding parallel features of heterogeneous mixtures. Nonetheless, to explore the capacity to use AIE as a phenomenon in the development of agents for molecular brachytherapy, data were collected concerning the absorption and emission of the compounds **14**, **15**, **18**, **19**, and **19-I_2_** under various conditions. The data are qualitative in nature, which suffices for the research thrust toward molecular brachytherapy.

The absorption spectra of **14**, **15**, **18**, **19**, and **19-I_2_** (20 μM) were collected in water (containing 0.1% DMSO from the stock solution). The spectra are shown in Figure 6C. Compounds **18**, **19**, and **19-I_2_** showed very similar spectra in the aqueous solution as in ethanol. On the other hand, compounds **14** and **15** in aqueous solution gave no obvious peak, which is attributed to poor solubility and the presence of aggregated particles. Dilution of the samples of **14** and **15** to 2 μM gave no change in the spectra. Such broadened spectra are as expected for compounds that are largely insoluble in water and are as desired for molecular brachytherapy. Upon dissolution in DMSO, **14** and **15** displayed spectra resembling those in ethanol consistent with a homogeneous solution.

The emission spectra of the target compounds in aqueous solution (0.1% DMSO in H_2_O) are shown in Figure 6D. The spectral parameters for the five compounds are listed in Table 2. Compounds **14** and **15** emitted at ~530 nm; such a large Stokes shift is characteristic of ESIPT in the HBT compounds. Both compounds also exhibited AIE, which was readily detected visually upon UV illumination (Figure 6E). The observation of AIE in **14** and **15** solutions coheres with the absorption spectra. For **18**, **19**, and **19-I_2_**, the spectra were rather similar in aqueous solution with those in ethanol. Most importantly, no AIE features were observed in aqueous solution (Figure 6E). The observation of AIE in **14** and **15** but not **18**, **19**, and **19-I_2_** is as desired for the molecular brachytherapy process.

**Figure A1:**
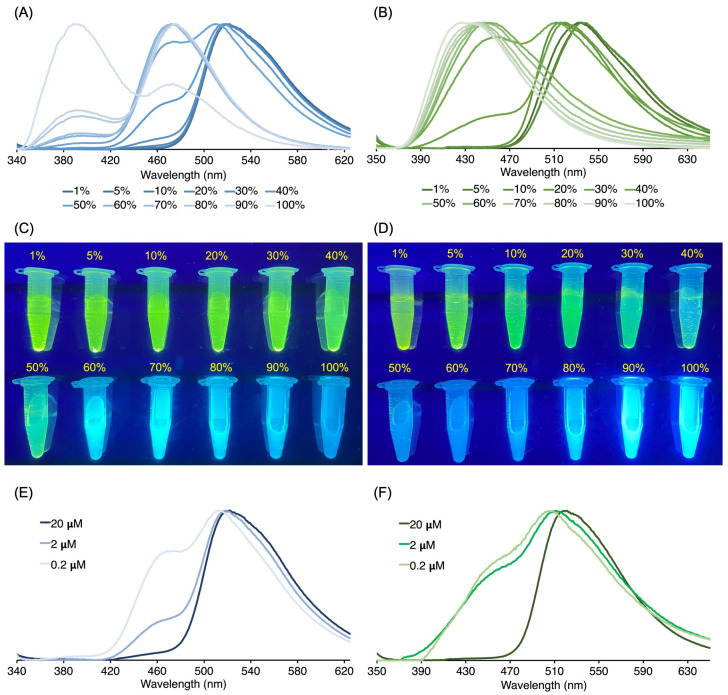
(**A**) Emission spectra (normalized) of **14** (20 μM) in various ratios of DMSO/H_2_O at room temperature (λ_exc_ = 318 nm). (**B**) Emission spectra (normalized) of **15** (20 μM) in various ratios of DMSO/H_2_O at room temperature (λ_exc_ = 330 nm). (**C**) Visualization (UV illumination) of **14** (20 μM) in various ratios of DMSO/H_2_O at room temperature. (**D**) Visualization (UV illumination) of **15** (20 μM) in various ratios of DMSO/H_2_O at room temperature. (**E**) Emission spectra (normalized) of **14** (with various concentrations) in H_2_O containing 20% DMSO at room temperature (λ_exc_ = 318 nm). (**F**) Emission spectra (normalized) of **15** (with various concentrations) in H_2_O containing 20% DMSO at room temperature (λ_exc_ = 330 nm).

A process of molecular brachytherapy must function at low concentration amidst environmental conditions ranging from physiological saline, interaction with membranous constituents, and the presence of diverse biomolecules. The properties of HBT derivatives **14** and **15**, which are the expected products of enzymatic treatment, were further examined in media comprised of various ratios of water and DMSO. Both water and DMSO are polar solvents. The results are shown in the Appendix A (Figure A1). The AIE phenomenon persists for **14** and **15** even in the presence of 40–50% DMSO.

The concentration dependence also was examined for **14** and **15** over the range 0.2–20 μM in water containing 20% DMSO (Figure A1). The AIE effect was diminished at 2 μM versus that at 20 μM.

**Figure A2:**
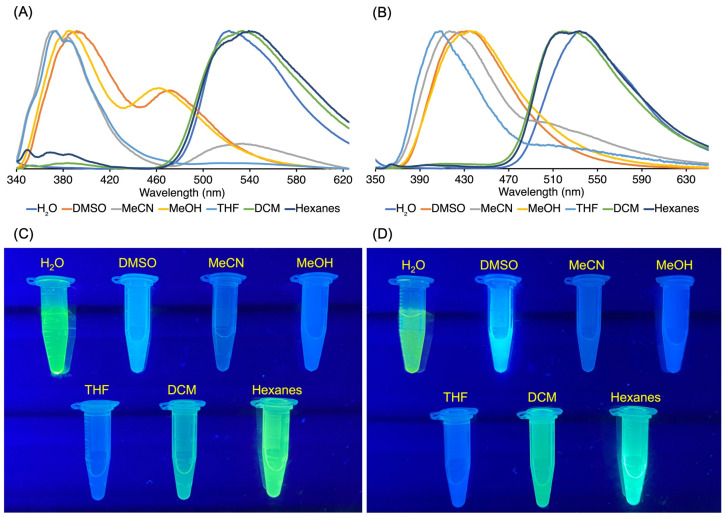
(**A**) Emission spectra (normalized) of **14** (20 μM) in various solvents at room temperature (λ_exc_ = 318 nm). (**B**) Emission spectra (normalized) of **15** (20 μM) in various solvents at room temperature (λ_exc_ = 330 nm). (**C**) Visualization (UV illumination) of **14** (20 μM) in various solvents at room temperature. (**D**) Visualization (UV illumination) of **15** (20 μM) in various solvents at room temperature.

Compounds **14** and **15** also were examined in organic solvents of lesser polarity than DMSO. The results are shown in the Appendix A (Figure A2) and are comparable to the solvent-dependent features exhibited by other HBT derivatives [53]. The AIE effect of **14** and **15**, at least with regards to formation of visible precipitates, was observed only in water.

### 3.4. Treatment with Phosphodiesterase and Alkaline Phosphatase Enzymes

**Scheme 7:**
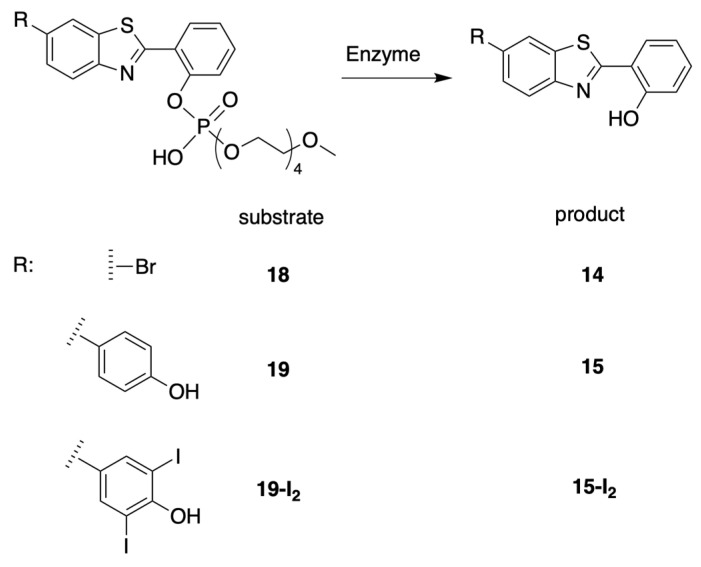
Enzymatic treatments of phosphodiester HBT derivatives.

To explore enzyme-triggered molecular brachytherapy, the three phosphoester substrates **18**, **19**, and **19-I_2_** were subjected to enzymatic assays (Scheme 7). All the enzyme assays were performed in aqueous media (0.1% DMSO in buffered aqueous solution). According to the previous studies of expected products upon enzymatic cleavage (**14**, **15**, and **15-I_2_**), aggregation of the resulting hydroxybenzothiazole unit and strong emission therefrom were expected.

**Figure 7:**
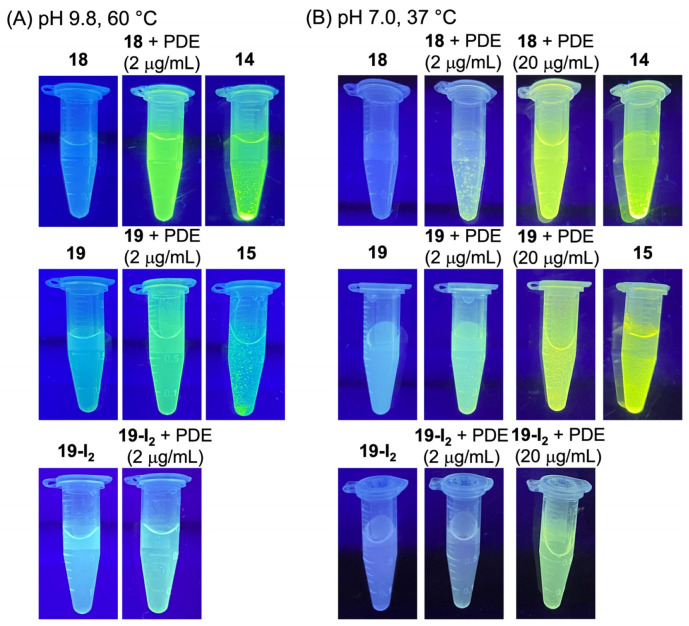
Visualization (UV illumination) of samples. (**A**) Treatment at pH 9.8 and 60 °C for 24 h. Left column—no enzyme with **18**, **19**, and **19-I_2_** (20 μM). Middle column—treatment of **18**, **19**, and **19-I_2_** (20 μM) with PDE (2 μg/mL). Right column—**14** and **15** (20 μM) in PDE buffer (positive controls). (**B**) Treatment under physiological condition (pH 7.0 and 37 °C) for 24 h. First column—no enzyme with **18**, **19**, and **19-I_2_** (20 μM). Second or third column—treatment of **18**, **19**, and **19-I_2_** (20 μM) with PDE (2 μg/mL) or PDE (20 μg/mL), respectively. Fourth column—**14** and **15** (20 μM) in PDE buffer (positive controls).

Three substrates (**18**, **19**, and **19-I_2_**) were treated with an exogeneous phosphodiesterase, the archaeal phosphodiesterase from *Methanococcus jannaschii* 0936. This phosphodiesterase (hereafter referred to as PDE) is a compact dimer (36 kDa) and exhibits optimal activity at 60–70 °C and pH 9.8 [29,54]. Hence, the first enzymatic assay was conducted under the optimal condition for PDE (pH 9.8, 60 °C). After incubating the three substrates (20 μM) with PDE (2 μg/mL) for 24 h, AIE (green) was unexpectedly observed in each case (Figure 7A). A similar weak green emission was also observed in the negative control (without addition of PDE) for **19-I_2_** after a 24 h incubation period, indicating the slight instability of **19-I_2_** at high temperature (60 °C).

For life sciences applications, activity at 37 °C and pH ~7.0 is preferred. The PDE still retains partial activity towards the standard substrate, bis(*p*-nitrophenyl) phosphate, under physiological conditions. Substrates **18**, **19**, and **19-I_2_** were then reexamined under physiological condition (pH 7.0, 37 °C) with two concentrations of PDE. Treatment with 2 μg/mL of PDE for 24 h resulted in AIE for all three substrates. On the other hand, treatment with 20 μg/mL of PDE resulted in stronger AIE for all three substrates (Figure 7B). The results show that the emission intensity is dependent upon enzyme concentration.

**Figure 8:**
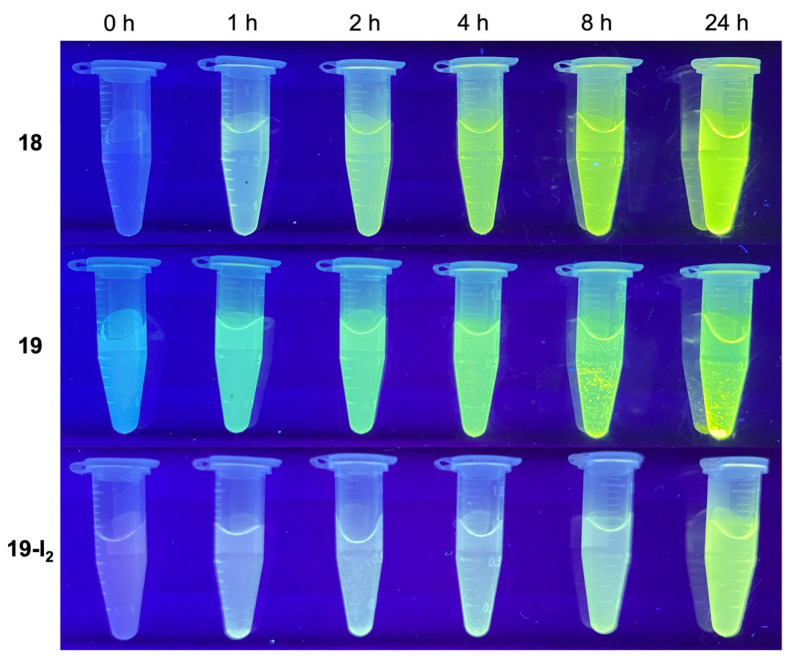
Visualization (UV illumination) upon treatment with PDE (20 μg/mL) under physiological condition (pH 7.0 and 37 °C) over time with substrates **18** (**top row**), **19** (**middle row**), and **19-I_2_** (**bottom row**), each at 20 μM.

The time course upon treatment of the three substrates with PDE under physiological conditions was then investigated. The cleavage of the phosphoester group and subsequent AIE from the resulting products could be detected after 1 h of incubation (Figure 8). The emission intensity increased with longer incubation time. 

**Figure 9:**
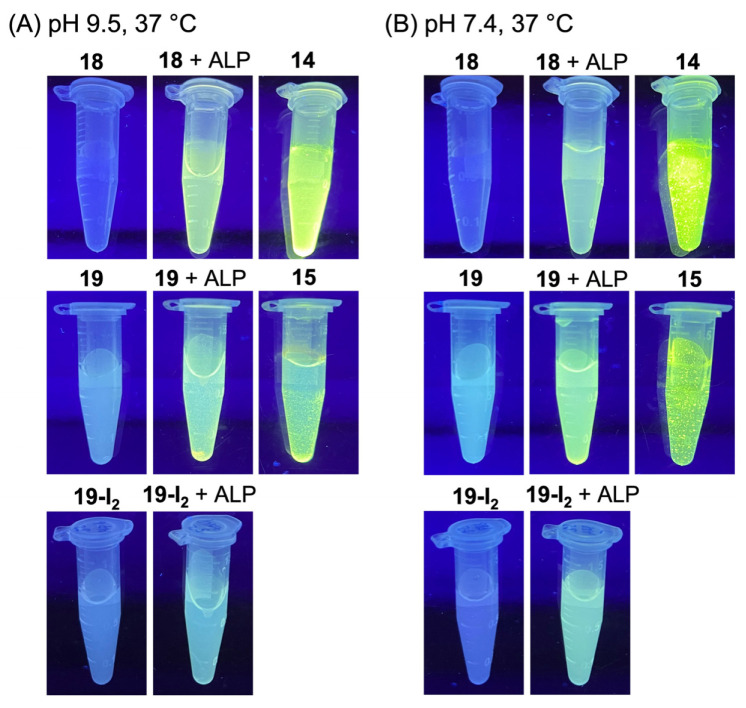
Visualization (UV illumination) of samples. (**A**) Treatment at pH 9.5 and 37 °C for 24 h. Left column—no enzyme with **18**, **19**, and **19-I_2_** (20 μM). Middle column—treatment of **18**, **19**, and **19-I_2_** (20 μM) with ALP (20 μg/mL). Right column—**14** and **15** (20 μM) in ALP buffer (positive controls). (**B**) Treatment under physiological condition (pH 7.4 and 37 °C) for 24 h. Left column—no enzyme with **18**, **19**, and **19-I_2_** (20 μM). Middle column—treatment of **18**, **19**, and **19-I_2_** (20 μM) with ALP (20 μg/mL). Right column—**14** and **15** (20 μM) in PBS buffer (positive controls).

**Figure A3:**
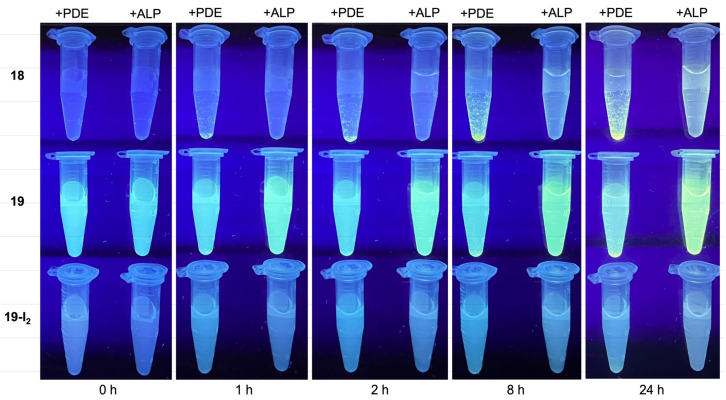
Visualization (UV illumination) over time upon enzymatic treatment (100 nM enzyme) under physiological conditions of **18** (top row), **19** (middle row), and **19-I_2_** (bottom row). Each sample was at 20 μM (left member of each pair: PDE treatment; right member of each pair: ALP treatment).

Alkaline phosphatase (ALP) enzymes, which catalyze the hydrolysis of a wide variety of phosphoester substrates, are found in many tissues of nearly all living organisms [35]. In general, ALP is comprised of a dimer (~160 kDa) and is stable in the pH range of 7.5–9.5. The hydrolysis of **18**, **19**, and **19-I_2_** was then examined under two different conditions to test the specificity towards ALP (Figure 9). Incubation for 24 h at pH 9.5 or pH 7.4 resulted in AIE under both conditions, but the emission intensity was noticeably weaker than that upon treatment with PDE. A direct comparison of the enzymatic treatments (PDE versus ALP) towards three target substrates (**18**, **19**, and **19-I_2_**) under physiological condition (pH ~7, 37 °C) was performed. The concentration of enzyme used in previous studies was 20 μg/mL, which corresponds to 555 nM for PDE (~36 kDa) and 125 nM for ALP (~160 kDa). For direct comparison, the concentration of both PDE and ALP was set here at 100 nM. The results are shown in the Appendix A (Figure A3). The enzyme PDE was found to cleave **18** and **19** more extensively to form the products that exhibit AIE than was that for the enzyme ALP. No significant difference was observed between the two enzymes for the substrate **19-I_2_**.

In a previous study [29], HBT phosphoester derivatives treated with ALP (0.2 μg/mL) gave no observable AIE, whereas mutant PDEs (0.2 μg/mL) showed significant emission. Here, a higher ALP concentration (20 μg/mL, ~0.2 U/mL) was used, which is more comparable with in vivo conditions. Concentrations of ALP in serum have been reported within the range of 44 to 147 U/L. While the three substrates (**18**, **19**, and **19-I_2_**) are cleaved to varying extent by the high concentration of ALP, far more extensive cleavage is observed upon treatment with the non-mutant PDE (the archaeal phosphodiesterase from *Methanococcus jannaschii* 0936). Further investigation of PDE mutants that exhibit higher specificity and faster rates of hydrolysis towards substrates will be required to differentiate the activity from endogenous phosphatase enzymes.

## 4. Materials and Methods

### 4.1. General Methods

^1^H and proto-decoupled ^13^C NMR spectra were recorded in CDCl_3_ at room temperature unless noted otherwise. Several ^13^C NMR peaks showed apparent splitting, which resulted in a higher count of resonances than the number of carbons. The splitting patterns are reported as individual singlets as shown in the ^13^C NMR spectra (ESI). ^31^P NMR spectroscopy was performed at room temperature with H_3_PO_4_ in D_2_O as external standard (referenced to 0.00 ppm), although in some instances the external standard was omitted without change in the observed ^31^P resonance. Absorption and emission spectra were collected in toluene at room temperature. Silica gel (40 μm average particle size) was used for column chromatography. Si-diol (functionalized silica) TLC plates and powder (40–63 μm) were purchased from SiliCycle. Commercial compounds were used as received. Compounds **1-Br** [40], **7** [29], and **7-Br** [29] were prepared as described in the literature.

### 4.2. Synthesis

*2-(2-Hydroxy-5-iodophenyl)-1,3-benzothiazole* (**1-I**). (A) Direct synthesis: following a reported method [40] with some modification, a suspension of 2-aminobenzenethiol (**2**, 84 μL, 0.81 mmol), 5-iodosalicylaldehyde (200 mg, 0.81 mmol), and AgNO_3_ (18 mg, 0.16 mmol) in DMSO (4.6 mL) was stirred at 30 °C for 24 h. The reaction was quenched by the addition of water, concentrated, and washed through a silica pad (3 cm × 5 cm) with CH_2_Cl_2_/hexanes (1:1). The crude mixture was concentrated and recrystallized in chloroform/ethanol to give a pale-yellow solid (136 mg, 48%): mp 173–174 °C; ^1^H NMR (CDCl_3_, 600 MHz) *δ* 12.58 (s, 1H), 7.99 (d, *J* = 8.1 Hz, 1H), 7.95 (d, *J* = 2.0 Hz, 1H), 7.92 (d, *J* = 7.8 Hz, 1H), 7.61 (dd, *J* = 8.7, 2.1 Hz, 1H), 7.54–7.50 (m, 1H), 7.45–7.42 (m, 1H), 6.89 (d, *J* = 8.7 Hz, 1H); ^13^C NMR (CDCl_3_, 150 MHz) *δ* 167.6, 157.7, 151.6, 141.1, 136.5, 132.6, 126.9, 125.9, 122.4, 121.6, 120.3, 119.1, 80.2; ESI-MS obsd 353.9448, calcd 353.9444 [(M + H)^+^, M = C_13_H_8_INOS].

(B) *Iodination of 2-(2-hydroxyphenyl)-1,3-benzothiazole* (**1**): following a reported method [41] with some modification, a suspension of **1** (262 mg, 1.15 mmol) and NaI (180 mg, 1.2 mmol) in methanol (12 mL) was treated with chloramine-T (680 mg, 2.4 mmol) under argon and stirred at room temperature for 6 h. The reaction was concentrated and washed with water to remove a soluble side product. The residue was then recrystallized in chloroform/ethanol to give the title compound with some 3,5-diiodinated byproduct. The NMR data of the major product are consistent with the one synthesized directly.

(C) *Conversion from 2-(2-hydroxy-5-bromophenyl)-1,3-benzothiazole* (**1-Br**): following a reported method [43] with some modification, a mixture of **1-Br** (163 mg, 0.532 mmol), CuI (10 mg, 53 μmol), and NaI (250 mg, 1.6 mmol) was placed in a Schlenk flask and degassed under reduced pressure for 30 min. Under a stream of argon, deaerated 1,4-dioxane (0.50 mL) and *trans-N,N’*-dimethylcyclohexane-1,2-diamine (9.0 μL, 57 μmol) were added into the flask. The suspension was stirred in an oil bath heated to 110 °C for 24 h, quenched by the addition of NH_3_·H_2_O (5 mL) and H_2_O (20 mL), extracted with CH_2_Cl_2_, and concentrated. Attempts at chromatography and recrystallization both gave **1-I** as the major product with residual **1-Br** remaining. The ratio was determined on the basis of ^1^H NMR spectroscopy.

*2-(2-Hydroxy-3,5-diiodophenyl)-1,3-benzothiazole* (**1-I_2_**). Following a reported method [41] with some modification, a suspension of **1** (50 mg, 0.22 mmol) and NaI (83 mg, 0.55 mmol) in methanol (3.0 mL) was treated with chloramine-T (310 mg, 1.1 mmol) under argon and stirred overnight at room temperature. The mixture was concentrated and washed with water to remove a soluble side product. The residue was then recrystallized in chloroform/ethanol to give a pale-yellow solid (61 mg, 58%): mp 172–174 °C; ^1^H NMR (CDCl_3_, 500 MHz) *δ* 13.72 (s, 1H), 8.11 (d, *J* = 2.0 Hz, 1H), 7.97 (d, *J* = 8.1 Hz, 1H), 7.93 (d, *J* = 8.1 Hz, 1H), 7.93 (d, *J* = 2.0 Hz, 1H), 7.57–7.51 (m, 1H), 7.49–7.44 (m, 1H); ^13^C NMR (CDCl_3_, 125 MHz) *δ* 166.7, 156.7, 151.0, 148.9, 136.5, 132.8, 127.2, 126.3, 122.4, 121.8, 118.4, 87.7, 80.7; ESI-MS obsd 479.8412, calcd 479.8411 [(M + H)^+^, M = C_13_H_7_I_2_NOS].

*2-Amino-5-bromobenzenethiol* (**2-Br**). Following a reported method [45] with some modification, a suspension of **13** (3.0 g, 13 mmol) and potassium hydroxide (14.7 g, 0.26 mol) in water (30 mL) was refluxed overnight. The reaction mixture was slowly acidified with AcOH at 4 °C, prompting precipitation. The suspension was filtered and washed with water. The solid residue on the filter paper was dissolved in CH_2_Cl_2_ and filtered through a silica pad (3 cm × 3 cm). Then, the filtrate was concentrated and recrystallized in EtOH, yielding a yellow-brown solid (1.53 g, 57%): mp 120–122 °C; ^1^H NMR (CD_3_OD, 500 MHz) *δ* 7.22 (dd, *J* = 8.6, 2.4 Hz, 1H), 7.07 (d, *J* = 2.4 Hz, 1H), 6.70 (d, *J* = 8.7 Hz, 1H); ^13^C NMR (CD_3_OD, 125 MHz) *δ* 150.2, 139.2, 135.3, 120.2, 117.8, 108.4; ESI-MS obsd 201.9331, calcd 201.9332 [(M + H)^+^, M = C_6_H_6_BrNS].

*Bis(2-(1,3-benzothiazol-2-yl)-4-iodophenyl) (2,5,8,11-tetraoxatridecan-13-yl) phosphate* (**6-I**). A solution of 2,5,8,11-tetraoxatridecan-13-ol (**4**, 10 μL, 50 μmol) in anhydrous CH_2_Cl_2_ (0.50 mL) was treated with phosphoryl chloride (4.7 μL, 50 μmol) under argon. The reaction mixture was stirred at room temperature for 3 h, followed by the addition of **1-I** (35 mg, 0.10 mmol) and triethylamine (50 μL, 0.33 mmol). The mixture was stirred at room temperature for 12 h and then loaded onto a silica column. Chromatography (silica, CH_2_Cl_2_ with 0% to 50% ethyl acetate) gave a colorless oil (31 mg, 65%): ^31^P NMR (CDCl_3_, 240 MHz) *δ* −13.40; ^1^H NMR (CDCl_3_, 600 MHz) *δ* 8.66 (s, 2H), 8.05 (d, *J* = 8.2 Hz, 2H), 7.76 (d, *J* = 8.0 Hz, 2H), 7.64 (dd, *J* = 8.7, 2.0 Hz, 2H), 7.50 (t, *J* = 7.7 Hz, 2H), 7.45 (d, *J* = 8.7 Hz, 2H), 7.39 (t, *J* = 7.6 Hz, 2H), 4.49 (dt, *J* = 8.9, 4.5 Hz, 2H), 3.7–3.69 (m, 2H), 3.58 (d, *J* = 7.4 Hz, 6H), 3.52–3.48 (m, 6H), 3.34 (s, 3H); ^13^C NMR (CDCl_3_, 150 MHz) *δ* 159.9, 152.2, 147.94, 147.90, 140.2, 138.8, 135.9, 126.91, 126.86, 126.5, 125.6, 123.4, 122.16, 122.15, 121.5, 89.5, 71.9, 70.60, 70.56, 70.52, 70.49, 70.57, 69.62, 69.58, 69.37, 69.3, 59.0; attempts to obtain mass spectral characterization (MALDI-MS) were not successful.

*(2-(1,3-Benzothiazol-2-yl)-4-iodophenyl) (2,5,8,11-tetraoxatridecan-13-yl) hydrogen phosphate* (**7-I**). A solution of **6-I** (27 mg, 28 μmol) in THF (1.0 mL) was treated with aqueous NaOH (5%, 30 μL, 38 μmol). The reaction mixture was stirred overnight at room temperature, quenched by washing through an ion exchange resin pad, and concentrated. Column chromatography (silica, CH_2_Cl_2_ with 0% to 20% methanol) yielded a light-brown oil (14 mg, 80%): ^31^P NMR (CDCl_3_, 240 MHz) *δ* –6.61; ^1^H NMR (CDCl_3_, 600 MHz) *δ* 8.74 (s, 1H), 8.07 (d, *J* = 8.1 Hz, 1H), 7.87 (d, *J* = 7.8 Hz, 1H), 7.64 (d, *J* = 7.1 Hz, 1H), 7.54–7.46 (m, 2H), 7.36 (t, *J* = 7.5 Hz, 1H), 4.17 (s, 2H), 3.58–3.43 (m, 15H), 3.32 (s, 3H); ^13^C NMR (CDCl_3_, 150 MHz) *δ* 161.4, 151.9, 149.9, 140.0, 137.7, 136.2, 126.2, 125.2, 122.9, 122.6, 121.5, 87.2, 71.4, 70.6, 70.2, 70.1, 70.01, 69.97, 66.4, 59.1; ESI-MS obsd 624.0307, calcd 624.0312 [(M + H)^+^, M = C_22_H_27_INO_8_PS].

*2-(2-Hydroxyphenyl)-6-phenoxy-1,3-benzothiazole* (**9a**). Following a reported method [44] with some modification, a suspension of 4-phenoxyaniline (**8a**, 55 mg, 0.30 mmol), salicylaldehyde (**3**, 21 μL, 0.20 mmol), S_8_ (32 mg, 1.0 mmol), and KI (10 mg, 60 μmol) in *N*-methyl-2-pyrrolidone (1.0 mL) was stirred at 150 °C under an atmosphere of O_2_ for 24 h. The reaction mixture was allowed to cool to room temperature and then was concentrated. Column chromatography (silica, hexanes/ethyl acetate (300:1 to 100:1)) gave a pale-yellow solid (18 mg, 28%): mp 140–142 °C; ^1^H NMR (CDCl_3_, 600 MHz) *δ* 12.35 (s, 1H), 7.91 (d, *J* = 8.8 Hz, 1H), 7.63 (dd, *J* = 7.8, 1.6 Hz, 1H), 7.44 (d, *J* = 2.4 Hz, 1H), 7.40–7.34 (m, 3H), 7.20 (dd, *J* = 8.8, 2.4 Hz, 1H), 7.16 (tt, *J* = 7.4, 1.1 Hz, 1H), 7.09 (dd, *J* = 8.3, 1.2 Hz, 1H), 7.08–7.05 (m, 2H), 6.94 (td, *J* = 7.5, 1.2 Hz, 1H); ^13^C NMR (CDCl_3_, 75 MHz) *δ* 168.3, 157.7, 157.0, 155.7, 147.9, 134.0, 132.6, 130.0, 128.3, 123.9, 123.0, 119.6, 119.2, 118.9, 117.8, 116.9, 110.6; ESI-MS obsd 320.0746, calcd 320.0740 [(M + H)^+^, M = C_19_H_13_NO_2_S].

*2-(2-Hydroxyphenyl)-6-(4-methoxyphenoxy)-1,3-benzothiazole* (**9b**). Following a reported method [44] with some modification, a suspension of 4-(4-methoxyphenoxy)aniline (**8b**, 160 mg, 0.75 mmol), salicylaldehyde (**3**, 53 μL, 0.50 mmol), S_8_ (160 mg, 5.0 mmol), and KI (50 mg, 0.30 μmol) in *N*-methyl-2-pyrrolidone (2.5 mL) was stirred at 150 °C under an atmosphere of O_2_ for 24 h. The reaction mixture was allowed to cool to room temperature and concentrated. Column chromatography (silica, hexanes/ethyl acetate (300:1 to 100:1)) gave a pale-yellow solid (54 mg, 21%): mp 145–147 °C; ^1^H NMR (CDCl_3_, 600 MHz) *δ* 12.36 (s, 1H), 7.89 (d, *J* = 8.9 Hz, 1H), 7.63 (dd, *J* = 7.8, 1.5 Hz, 1H), 7.38–7.34 (m, 2H), 7.17 (dd, *J* = 8.9, 2.5 Hz, 1H), 7.09 (dd, *J* = 8.3, 1.1 Hz, 1H), 7.06–7.02 (m, 2H), 6.97–6.90 (m, 3H), 3.83 (s, 3H); ^13^C NMR (CDCl_3_, 150 MHz) *δ* 167.9, 157.7, 157.0, 156.4, 149.8, 147.4, 134.0, 132.5, 128.2, 122.9, 121.1, 119.5, 118.0, 117.8, 116.9, 115.1, 109.0, 55.7; ESI-MS obsd 350.0852, calcd 350.0845 [(M + H)^+^, M = C_20_H_15_NO_3_S].

*2-(2-Hydroxyphenyl)-6-(3-methoxyphenoxy)-1,3-benzothiazole* (**9c**). Following a reported method [44] with some modification, a suspension of 4-(3-methoxyphenoxy)aniline (**8c**, 162 mg, 0.75 mmol), salicylaldehyde (**3**, 53 μL, 0.50 mmol), S_8_ (160 mg, 5.0 mmol), and KI (86 mg, 0.50 μmol) in *N*-methyl-2-pyrrolidone (2.0 mL) was stirred at 150 °C under an atmosphere of O_2_ for 24 h. The reaction mixture was allowed to cool to room temperature and concentrated. Column chromatography (silica, hexanes/ CH_2_Cl_2_ (300:1 to 100:1)) gave a pale-yellow solid (54 mg, 21%): mp 125–127 °C; ^1^H NMR (CDCl_3_, 500 MHz) *δ* 12.35 (s, 1H), 7.93 (d, *J* = 8.8 Hz, 1H), 7.65 (dd, *J* = 7.8, 1.6 Hz, 1H), 7.47 (d, *J* = 2.4 Hz, 1H), 7.40–7.35 (m, 1H), 7.31–7.25 (m, 1H), 7.22 (dd, *J* = 8.8, 2.4 Hz, 1H), 7.10 (d, *J* = 8.3, 1H), 6.97–6.93 (m, 1H), 6.75–6.69 (m, 1H), 6.67–6.58 (m, 2H), 3.80 (s, 3H); ^13^C NMR (CDCl_3_, 125 MHz) *δ* 168.4, 161.1, 158.2, 157.7, 155.4, 148.0, 133.9, 132.6, 130.4, 128.3, 123.0, 119.6, 119.0, 117.82, 116.8, 111.2, 110.8, 109.5, 105.2, 55.4; ESI-MS obsd 350.0851, calcd 350.0845 [(M + H)^+^, M = C_20_H_15_NO_3_S].

*Diethyl (2-(6-phenoxy-1,3-benzothiazol-2-yl)phenyl) phosphate* (**10a**). A sample of **9a** (13 mg, 40 μmol) was dissolved in anhydrous CH_2_Cl_2_ (1.0 mL) under argon. The homogeneous mixture was then treated with diethyl phosphorochloridate (2.3 μL, 16 μmol). Triethylamine (3.0 μL, 19 μmol) was added dropwise, and the mixture was stirred overnight at room temperature. Column chromatography (silica, CH_2_Cl_2_ with 0% to 16% ethyl acetate) afforded a non-crystalline white solid (5.3 mg, 73%): ^31^P NMR (CDCl_3_, 160 MHz) *δ* −6.94; ^1^H NMR (CDCl_3_, 600 MHz) *δ* 8.42 (dt, *J* = 7.9, 1.6 Hz, 1H), 8.04 (d, *J* = 8.8 Hz, 1H), 7.65 (d, *J* = 8.3 Hz, 1H), 7.49 (d, *J* = 2.4 Hz, 1H), 7.46 (ddd, *J* = 8.4, 7.3, 1.8 Hz, 1H), 7.41–7.36 (m, 2H), 7.32 (t, *J* = 7.6 Hz, 1H), 7.23 (dd, *J* = 8.8, 2.4 Hz, 1H), 7.16 (t, *J* = 7.4 Hz, 1H), 7.07 (d, *J* = 7.4 Hz, 2H), 4.31–4.20 (m, 4H), 1.33 (t, *J* = 7.1 Hz, 6H); ^13^C NMR (CDCl_3_, 150 MHz) *δ* 161.2, 157.2, 155.2, 148.6, 148.6, 148.5, 137.3, 131.6, 130.1, 129.9, 125.1, 124.7, 124.6, 124.0, 123.7, 119.91, 119.90, 119.1, 118.8, 110.3, 65.10, 65.06, 16.15, 16.11; attempts to obtain mass spectral characterization (ESI-MS) were not successful.

*Diethyl (2-(6-(4-methoxyphenoxy)-1,3-benzothiazol-2-yl)phenyl) phosphate* (**10b**). A sample of **9b** (19 mg, 54 μmol) was dissolved in anhydrous CH_2_Cl_2_ (1.0 mL) under argon. The homogeneous mixture was then treated with diethyl phosphorochloridate (10 μL, 65 μmol). Triethylamine (17 μL, 0.11 mmol) was added dropwise, and the mixture was stirred overnight at room temperature. Column chromatography (silica, CH_2_Cl_2_ with 0% to 5% ethyl acetate) afforded a white solid (19 mg, 72%): mp 87–89 °C; ^31^P NMR (CDCl_3_, 160 MHz) *δ*–6.95; ^1^H NMR (CDCl_3_, 600 MHz) *δ* 8.40 (dt, *J* = 7.9, 1.6 Hz, 1H), 8.01 (d, *J* = 8.8 Hz, 1H), 7.65 (dt, *J* = 8.4, 1.1 Hz, 1H), 7.47–7.42 (m, 1H), 7.39 (d, *J* = 2.5 Hz, 1H), 7.31 (td, *J* = 7.7, 1.2 Hz, 1H), 7.19 (dd, *J* = 8.9, 2.5 Hz, 1H), 7.06–7.02 (m, 2H), 6.95–6.90 (m, 2H), 4.31–4.19 (m, 4H), 3.83 (s, 3H), 1.35–1.29 (m, 6H); ^13^C NMR (CDCl_3_, 150 MHz) *δ* 160.8, 156.6, 156.2, 150.1, 148.54, 148.49, 148.2, 137.3, 131.5, 130.1, 125.1, 124.7, 124.7, 123.9, 121.0, 119.91, 119.90, 117.9, 115.0, 108.8, 65.1, 65.0, 55.7, 16.15, 16.11; ESI-MS obsd 486.1138, calcd 486.1135 [(M + H)^+^, M = C_24_H_24_NO_6_PS].

*Diethyl (2-(6-(3-methoxyphenoxy)-1,3-benzothiazol-2-yl)phenyl) phosphate* (**10c**). A sample of **9c** (11 mg, 30 μmol) was dissolved in anhydrous CH_2_Cl_2_ (0.50 mL) under argon. The homogeneous mixture was then treated with diethyl phosphorochloridate (5.8 μL, 40 μmol). Triethylamine (9.2 μL, 60 mmol) was added dropwise, and the mixture was stirred overnight at room temperature. The reaction mixture was concentrated and loaded onto a preparative TLC plate (25 cm × 25 cm, 0.25 mm). Chromatography (R_f_ = 0.30, silica, hexanes/ethyl acetate (2:1)) gave a non-crystalline white solid (6.0 mg, 41%): ^31^P NMR (CDCl_3_, 160 MHz) *δ*–6.95; ^1^H NMR (CDCl_3_, 600 MHz) *δ* 8.42 (dt, *J* = 7.9, 1.6 Hz, 1H), 8.04 (d, *J* = 8.8 Hz, 1H), 7.66 (dt, *J* = 8.4, 1.1 Hz, 1H), 7.52 (d, *J* = 2.4 Hz, 1H), 7.50–7.43 (m, 1H), 7.32 (t, J = 7.5 Hz, 1H), 7.28–7.25 (m, 1H), 7.24 (dd, J = 8.8, 2.4 Hz, 1H), 6.73–6.68 (m, 1H), 6.66–6.61 (m, 2H), 4.31–4.21 (m, 4H), 3.80 (s, 3H), 1.35–1.31 (m, 6H); ^13^C NMR (CDCl_3_, 150 MHz) *δ* 161.3, 161.1, 158.5, 154.9, 148.7, 148.6, 148.5, 137.3, 131.6, 130.3, 130.1, 125.1, 124.7, 124.6, 124.0, 119.91, 119.89, 118.9, 111.2, 110.6, 109.3, 105.1, 65.12, 65.08, 55.4, 16.2, 16.1; ESI-MS obsd 486.1141, calcd 486.1135 [(M + H)^+^, M = C_24_H_24_NO_6_PS].

*2-(2-Hydroxy-3,5-diiodophenyl)-6-phenoxy-1,3-benzothiazole* (**12a**). A suspension of **9a** (4.5 mg, 14 μmol) and NaI (10 mg, 67 μmol) in methanol (0.50 mL) was treated with chloramine-T (40 mg, 0.14 mmol) under argon and stirred overnight at room temperature. The reaction mixture was concentrated and loaded onto a preparative TLC plate (25 cm x 25 cm, 0.25 mm). Chromatography (R_f_ = 0.30, silica, hexanes/ CH_2_Cl_2_ (3:1)) gave a white solid (4.7 mg, 59%): mp 184–186 °C; ^1^H NMR (CDCl_3_, 600 MHz) *δ* 13.56 (b, 1H), 8.10 (d, *J* = 2.0 Hz, 1H), 7.91 (d, *J* = 8.9 Hz, 1H), 7.87 (d, *J* = 2.0 Hz, 1H), 7.45 (d, *J* = 2.4 Hz, 1H), 7.43–7.38 (m, 2H), 7.24 (dd, *J* = 8.9, 2.4 Hz, 1H), 7.19 (tt, *J* = 7.5, 1.1 Hz, 1H), 7.10–7.06 (m, 2H); ^13^C NMR (CDCl_3_, 150 MHz) *δ* 165.4, 156.6, 156.5, 156.4, 148.7, 147.0, 136.3, 134.2, 130.1, 124.2, 123.3, 119.5, 119.3, 118.5, 110.3, 87.6, 80.7; ESI-MS obsd 571.8672, calcd 571.8673 [(M + H)^+^, M = C_19_H_11_I_2_NO_2_S].

*2-(2-Hydroxy-3,5-diiodophenyl)-6-(4-methoxyphenoxy)-1,3-benzothiazole* (**12b**). A suspension of **9b** (5.0 mg, 14 μmol) and NaI (10 mg, 67 μmol) in methanol (0.50 mL) was treated with chloramine-T (40 mg, 0.14 mmol) under argon and stirred overnight at room temperature. The reaction mixture was concentrated and loaded onto a preparative TLC plate (25 cm x 25 cm, 0.25 mm). Chromatography (R_f_ = 0.30, silica, hexanes/ CH_2_Cl_2_ (3:1)) gave a white solid (7.0 mg, 83%): mp 188–190 °C; ^1^H NMR (CDCl_3_, 600 MHz) *δ* 13.56 (b, 1H), 8.09 (d, *J* = 2.0 Hz, 1H), 7.87 (d, *J* = 8.9 Hz, 1H), 7.85 (d, *J* = 2.0 Hz, 1H), 7.35 (d, *J* = 2.4 Hz, 1H), 7.20 (dd, *J* = 8.9, 2.5 Hz, 1H), 7.06–7.02 (m, 2H), 6.97–6.91 (m, 2H), 3.84 (s, 3H); ^13^C NMR (CDCl_3_, 150 MHz) *δ* 165.0, 157.7, 156.5, 156.4, 149.4, 148.5, 146.5, 136.2, 134.2, 123.2, 121.3, 118.6, 118.4, 115.2, 108.8, 87.5, 80.7, 55.7; ESI-MS obsd 601.8781, calcd 601.8778 [(M + H)^+^, M = C_20_H_13_I_2_NO_3_S].

*2-(2-Hydroxy-3,5-diiodophenyl)-6-(3-methoxyphenoxy)-1,3-benzothiazole* (**12c**). A suspension of **9c** (5.0 mg, 14 μmol) and NaI (10 mg, 67 μmol) in methanol (0.50 mL) was treated with chloramine-T (40 mg, 0.14 mmol) under argon and stirred overnight at room temperature or 50 °C. Several crude samples upon carrying out this reaction were combined, concentrated, and loaded onto a preparative TLC plate (25 cm × 25 cm, 1.0 mm). Chromatography (R_f_ = 0.50, silica, hexanes/CH_2_Cl_2_ (2:1)) gave a white solid: mp 124–125 °C; ^1^H NMR (CDCl_3_, 600 MHz) *δ* 8.10 (d, *J* = 2.0 Hz, 1H), 7.91 (d, *J* = 8.9 Hz, 1H), 7.87 (d, *J* = 1.9 Hz, 1H), 7.47 (d, *J* = 2.4 Hz, 1H), 7.29 (t, *J* = 8.2 Hz, 1H), 7.26–7.23 (m, 1H), 6.75–6.72 (m, 1H), 6.67–6.64 (m, 1H), 6.63 (t, *J* = 2.3 Hz, 1H), 3.81 (s, 3H); ^13^C NMR (CDCl_3_, 150 MHz) *δ* 165.5, 161.2, 157.8, 156.5, 156.2, 148.7, 147.1, 136.3, 134.2, 130.5, 123.7, 119.4, 118.5, 111.5, 110.5, 109.7, 105.5, 87.6, 80.7, 77.2, 77.0, 76.8, 55.5; ESI-MS obsd 601.8779, calcd 601.8778 [(M + H)^+^, M = C_20_H_13_I_2_NO_3_S].

*6-Bromo-2-(2-hydroxyphenyl)-1,3-benzothiazole* (**14**). Following a reported method [40] with some modification, a suspension of **2-Br** (210 mg, 1.0 mmol), salicylaldehyde (**3**, 130 μL, 1.2 mmol), and AgNO_3_ (110 mg, 1.0 mmol) in DMSO (10 mL) was stirred under argon at 100 °C for 9 h. The reaction was allowed to cool to room temperature, washed through a silica pad (3 cm × 3 cm) with CH_2_Cl_2_, and concentrated. The crude mixture was then recrystallized in ethanol/ethyl acetate to give a pale-yellow solid (130 mg, 41%): mp 203–205 °C; ^1^H NMR (CDCl_3_, 600 MHz) *δ* 12.23 (s, 1H), 8.03 (d, *J* = 1.9 Hz, 1H), 7.83 (d, *J* = 8.6 Hz, 1H), 7.66 (dd, *J* = 7.8, 1.5 Hz, 1H), 7.60 (dd, *J* = 8.6, 1.9 Hz, 1H), 7.40 (ddd, *J* = 8.5, 7.2, 1.5 Hz, 1H), 7.10 (dd, *J* = 8.3, 1.1 Hz, 1H), 6.96 (td, *J* = 7.6, 1.1 Hz, 1H); ^13^C NMR (CDCl_3_, 150 MHz) *δ* 169.8, 158.0, 150.8, 134.3, 133.2, 130.2, 128.4, 124.1, 123.2, 119.7, 119.1, 118.0, 116.4; ESI-MS obsd 305.9589, calcd 305.9582 [(M + H)^+^, M = C_13_H_8_BrNOS].

*6-(4-Hydroxyphenyl)-2-(2-hydroxyphenyl)-1,3-benzothiazole* (**15**). A solution of 1,4-dioxane/H_2_O (2:1) was deaerated with argon for 1 h. A mixture of **14** (62 mg, 0.20 mmol), 4-(4,4,5,5-tetramethyl-1,3,2-dioxaborolan-2-yl)phenol (**XIII**, 84 mg, 0.38 mmol), Pd(PPh_3_)_2_Cl_2_ (14 mg, 0.020 mmol), and CsF (120 mg, 0.80 mmol) was placed in a Schlenk flask, which was then subjected to high vacuum for 30 min. The Schlenk flask was then filled with argon followed by the addition of degassed 1,4-dioxane/H_2_O (1.0 mL, 2:1). The reaction mixture was heated to 90 °C, stirred for 90 min, allowed to cool to room temperature, and concentrated. Column chromatography (silica, CH_2_Cl_2_) followed by recrystallization in chloroform/hexanes, gave a yellow solid (42 mg, 65%): mp 224–225 °C; ^1^H NMR (THF-*d*_8_, 500 MHz) *δ* 12.20 (s, 1H), 8.46 (s, 1H), 8.18 (s, 1H), 8.00 (d, *J* = 8.4 Hz, 1H), 7.78–7.71 (m, 2H), 7.58–7.52 (m, 2H), 7.39–7.31 (m, 1H), 7.03 (d, *J* = 8.3 Hz, 1H), 6.93 (t, *J* = 7.5 Hz, 1H), 6.88–6.83 (m, 2H); ^13^C NMR (THF-*d*_8_, 125 MHz) *δ* 169.5, 158.8, 158.6, 151.3, 139.9, 134.3, 133.2, 131.8, 129.0, 128.8, 126.4, 122.6, 120.0, 119.5, 118.3, 117.6, 116.4; ESI-MS obsd 320.0742, calcd 320.0740 [(M + H)^+^, M = C_19_H_13_NO_2_S].

*6-(2-(3-Hydroxyphenyl)ethynyl)-2-(2-hydroxyphenyl)-1,3-benzothiazole* (**16**). A solution of THF/triethylamine (5:1) was deaerated with argon for 1 h. A mixture of **14** (30 mg, 98 μmol), Pd(PPh_3_)_2_Cl_2_ (7.0 mg, 10 μmol), and CuI (3.8 mg, 20 μmol) was placed in a Schlenk flask, which was subjected to high vacuum for 30 min. The Schlenk flask was then filled with argon followed by the addition of degassed THF/triethylamine (1.0 mL, 5:1) and 3-ethynylphenol (**XII**, 13 μL, 0.12 mmol). The reaction mixture was heated to 60 °C, stirred overnight, allowed to cool to room temperature, and concentrated. Column chromatography (Si-diol silica, CH_2_Cl_2_) gave an impure mixture. Further washing with CH_2_Cl_2_ afforded a pure yellow solid (10 mg, 30%): mp 208–210 °C; ^1^H NMR (THF-*d*_8_, 600 MHz) *δ* 12.01 (s, 1H), 8.50 (s, 1H), 8.18 (d, *J* = 1.6 Hz, 1H), 8.00 (d, *J* = 8.4 Hz, 1H), 7.80 (dd, *J* = 7.8, 1.6 Hz, 1H), 7.65 (dd, *J* = 8.4, 1.6 Hz, 1H), 7.41–7.35 (m, 1H), 7.16 (t, *J* = 7.9 Hz, 1H), 7.04 (d, *J* = 8.3 Hz, 1H), 6.99 (dt, *J* = 7.6, 1.3 Hz, 1H), 6.98–6.92 (m, 2H), 6.80–6.75 (m, 1H); ^13^C NMR (THF-*d*_8_, 150 MHz) *δ* 171.0, 158.9, 158.5, 152.3, 133.9, 133.7, 130.9, 130.1, 129.2, 125.5, 124.6, 123.3, 122.6, 121.6, 120.2, 118.7, 118.4, 117.5, 116.8, 91.2, 88.7; ESI-MS obsd 344.0740, calcd 344.0744 [(M + H)^+^, M = C_21_H_13_NO_2_S].

*Bis(2-(6-bromo-1,3-benzothiazol-2-yl)phenyl) (2,5,8,11-tetraoxatridecan-13-yl) phosphate* (**17**). A solution of 2,5,8,11-tetraoxatridecan-13-ol (80 μL, 0.40 mmol) in anhydrous CH_2_Cl_2_ (4.0 mL) was treated with phosphoryl chloride (37 μL, 0.40 mmol) under argon. The reaction mixture was stirred at room temperature for 3 h, followed by the addition of **14** (245 mg, 0.80 mmol) and triethylamine (200 μL, 1.3 mmol). The mixture was stirred at room temperature for 12 h and then chromatographed (silica, ethyl acetate) to give a colorless oil (150 mg, 56%): ^31^P NMR (CDCl_3_, 240 MHz) *δ* –13.12; ^1^H NMR (CDCl_3_, 600 MHz) *δ* 8.32 (d, *J* = 7.9 Hz, 2H), 7.87 (d, *J* = 8.7 Hz, 2H), 7.84 (d, *J* = 2.0 Hz, 2H), 7.72 (d, *J* = 8.3 Hz, 2H), 7.56 (dd, *J* = 8.6, 2.0 Hz, 2H), 7.44–7.38 (m, 2H), 7.29 (t, *J* = 7.6 Hz, 2H), 4.54–4.48 (m, 2H), 3.76–3.70 (m, 2H), 3.61–3.57 (m, 6H), 3.55–3.48 (m, 6H), 3.34 (s, 3H); ^13^C NMR (CDCl_3_, 150 MHz) *δ* 162.3, 151.2, 148.3, 148.2, 137.5, 132.0, 130.4, 129.8, 125.7, 124.6, 124.5, 124.3, 123.9, 120.19, 120.18, 118.8, 71.9, 70.63, 70.57, 70.54, 70.52, 70.48, 69.71, 69.67, 69.14, 69.10, 59.01; ESI-MS obsd 862.9855, calcd 862.9856 [(M + H)^+^, M = C_35_H_33_Br_2_N_2_O_8_PS_2_].

*(2-(6-Bromo-1,3-benzothiazol-2-yl)phenyl) (2,5,8,11-tetraoxatridecan-13-yl) hydrogen phosphate* (**18**). A solution of **17** (39 mg, 45 μmol) in THF (1.0 mL) was treated with aqueous NaOH (5%, 90 μL, 112 μmol). The reaction mixture was stirred at room temperature for 4 h, quenched by the addition of ion exchange resin and Na_2_SO_4_, and concentrated. Column chromatography (silica, CH_2_Cl_2_ with 0% to 20% methanol) yielded a light-brown oil (15 mg, 76%): ^31^P NMR (CDCl_3_, 200 MHz) *δ* –6.61; ^1^H NMR (CDCl_3_, 600 MHz) *δ* 8.35 (d, *J* = 7.8 Hz, 1H), 7.96 (s, 1H), 7.87 (d, *J* = 8.7 Hz, 2H), 7.50 (d, *J* = 8.6 Hz, 1H), 7.30 (b, 1H), 7.09 (t, *J* = 7.5 Hz, 1H), 4.13 (b, 2H), 3.47 (b, 8H), 3.41 (b, 6H), 3.28 (s, 3H); ^13^C NMR (CDCl_3_, 150 MHz) *δ* 164.0, 151.1, 138.0, 131.8, 129.3, 129.1, 124.0, 123.8, 123.5, 123.1, 120.2, 118.0, 71.4, 70.8, 69.87, 69.83, 69.80, 65.5, 59.0; ESI-MS obsd 576.0458, calcd 576.0451 [(M + H)^+^, M = C_22_H_27_BrNO_8_PS].

*(2-(6-(4-Hydroxyphenyl)-1,3-benzothiazol-2-yl)phenyl) (2,5,8,11-tetraoxatridecan-13-yl) hydrogen phosphate* (**19**). A solution of 1,4-dioxane/H_2_O (2:1) was deaerated with argon for 1 h. A mixture of **18** (40 mg, 69 μmol), 4-(4,4,5,5-tetramethyl-1,3,2-dioxaborolan-2-yl)phenol (**XIII**, 25 mg, 115 μmol), Pd(PPh_3_)_2_Cl_2_ (10 mg, 14 μmol), and CsF (35 mg, 0.23 mmol) was placed in a Schlenk flask, which was then subjected to high vacuum for 30 min. The Schlenk flask was then filled with argon followed by the addition of degassed 1,4-dioxane/H_2_O (0.50 mL, 2:1). The reaction mixture was heated to 90 °C, stirred for 90 min, allowed to cool to room temperature, and concentrated. Column chromatography (silica, CH_2_Cl_2_ with 15% methanol) gave a brown oil (37 mg, 91%): ^31^P NMR (CD_3_OD, 200 MHz) *δ*–5.62, ^1^H NMR (CD_3_OD, 500 MHz) *δ* 8.36 (d, *J* = 7.7 Hz, 1H), 8.12–8.08 (m, 1H), 8.00 (d, *J* = 8.5 Hz, 1H), 7.80 (d, *J* = 8.3 Hz, 1H), 7.68 (dd, *J* = 8.5, 1.6 Hz, 1H), 7.52 (d, *J* = 8.5 Hz, 2H), 7.49–7.42 (m, 1H), 7.21 (t, J = 7.6 Hz, 1H), 6.89 (d, J = 8.5 Hz, 2H), 4.18–4.10 (m, 2H), 3.61 (t, *J* = 4.8 Hz, 2H), 3.53–3.47 (m, 10H), 3.473.41 (m, 2H), 3.29 (s, 3H); ^13^C NMR (CD_3_OD, 125 MHz) *δ* 164.9, 158.6, 152.6, 152.5, 151.9, 139.7, 138.2, 133.0, 132.7, 130.0, 129.4, 126.4, 125.3, 125.2, 124.3, 123.4, 121.5, 119.7, 116.8, 72.7, 71.8, 71.7, 71.3, 71.20, 71.18, 71.1, 71.0, 66.73, 66.68, 59.2; ESI-MS obsd 590.1603, calcd 590.1608 [(M + H)^+^, M = C_28_H_32_NO_9_PS].

*(2-(6-(4-Hydroxy-3,5-diiodophenyl)-1,3-benzothiazol-2-yl)phenyl) (2,5,8,11-tetraoxatridecan-13-yl) hydrogen phosphate* (**19-I_2_**). A suspension of **19** (18.5 mg, 31 μmol) and NaI (14 mg, 94 μmol) in methanol (3.7 mL) was treated with chloramine-T (27 mg, 94.0 μmol) and stirred at room temperature for 2 h. The reaction mixture was quenched by the addition of saturated Na_2_S_2_O_3_ solution (1.5 mL) before drying under a high vacuum. Column chromatography (silica, CH_2_Cl_2_ with 5% to 10% methanol) gave a brown non-crystalline solid (24 mg, 92%): ^31^P NMR (CD_3_OD, 200 MHz) *δ –*5.68; ^1^H NMR (CD_3_OD, 600 MHz) *δ* 8.38 (d, *J* = 8.0 Hz, 1H), 8.15 (s, 1H), 8.05 (s, 2H), 8.03 (d, *J* = 8.6 Hz, 1H), 7.80 (d, *J* = 8.4 Hz, 1H), 7.65 (dd, *J* = 8.5, 1.9 Hz, 1H), 7.50–7.44 (m, 1H), 7.26–7.20 (m, 1H), 4.18–4.12 (m, 2H), 3.64 (t, *J* = 4.6 Hz, 2H), 3.57–3.49 (m, 10H), 3.47–3.44 (m, 2H), 3.30 (s, 3H); ^13^C NMR (CD_3_OD, 150 MHz) *δ* 165.7, 157.1, 152.72, 152.69, 152.6, 139.2, 138.3, 137.4, 136.4, 132.8, 130.0, 126.3, 125.16, 125.11, 124.2, 123.7, 121.4, 120.4, 86.2, 72.8, 71.79, 71.74, 71.42, 71.34, 71.27, 71.14, 66.79, 66.75, 59.2; ESI-MS obsd 841.9531, calcd 841.9541 [(M + H)^+^, M = C_28_H_30_I_2_NO_9_PS].

### 4.3. Assays–Methods

#### 4.3.1. Enzymes and Buffers

Phosphodiesterase was from *Methanococcus jannaschii* 0936 (PDE). Alkaline phosphatase from bovine intestinal mucosa (ALP) was purchased from Millipore Sigma (St. Louis, MO). PDE buffer (pH 9.8) contains 50 mM diethanolamine, 40 mM NaCl, and 1 mM NiCl_2_. PDE buffer (pH 7.0) contains 50 mM 3-(*N*-morpholino)propanesulfonic acid (MOPS), 40 mM NaCl, and 1 mM NiCl_2_. PDE buffer was prepared without Ni^2+^ to avoid precipitation; instead, an aliquot of a NiCl_2_ stock solution (1 M in H_2_O) was added before each experiment to complete the buffer preparation. ALP buffer (pH 9.5) contains 100 mM NaCl, 100 mM Tris-Cl, and 5 mM MgCl_2_. PBS buffer (1x, pH 7.4) contains 137 mM NaCl, 2.7 mM KCl, 8 mM Na_2_HPO_4_, and 2 mM KH_2_PO_4_.

#### 4.3.2. Absorption and Emission Measurements

Compounds **14**, **15**, **18**, **19**, and **19-I_2_** were dissolved in DMSO to prepare a 20 mM stock solution. The stock solution of each compound was diluted with ethanol to prepare a sample solution with A ~0.1 (<0.05% DMSO). The same sample solution was subjected to emission measurements and determination of the fluorescence quantum yield. A quantity of 1 μL of the stock solution was mixed with 999 μL of deionized water to prepare a 20 μM solution. Absorption and emission spectra were then recorded. All measurements of emission spectra, including fluorescence quantum yield determinations, were carried out using a Horiba Duetta–Bio instrument at room temperature.

#### 4.3.3. AIE Visualization

Studies to detect AIE were carried by placing an Eppendorf tube containing the reaction mixture on a high-performance UV transilluminator (TFM-20, Ultra-Violet Products). Images were recorded by photography using the camera in an iPhone 13-Pro. 

### 4.4. Assays–Experiments

#### 4.4.1. PDE with Target Compounds under Basic Conditions

Substrate **18**, **19**, or **19-I_2_** (1 μL, 20 mM in DMSO) was first mixed with 999 μL of PDE buffer (pH 9.8) to prepare a 20 μM solution. Then, 20 μL of PDE stock solution (100 μg/mL in H_2_O) was added. The reaction mixture was incubated at 60 °C for 24 h. Substrates diluted with buffer (without addition of enzyme) served as negative control. Emission was then observed with the UV transilluminator. Data are shown in Figure 7A.

#### 4.4.2. PDE with Target Compounds under Physiological Conditions

Substrate **18**, **19**, or **19-I_2_** (1 μL, 20 mM in DMSO) was first mixed with 999 μL of PDE buffer (pH 7.0) to prepare a 20 μM solution. Then, 20 μL of PDE stock solution (100 μg/mL or 1 mg/mL in H_2_O) was added. The reaction mixture was incubated at 37 °C for 24 h. Substrates diluted with buffer (without addition of enzyme) served as negative control. Emission was then observed with the UV transilluminator. Data are shown in Figure 7B.

#### 4.4.3. PDE Timecourse with Target Compounds under Physiological Conditions

Substrate **18**, **19**, or **19-I_2_** (1 μL, 20 mM in DMSO) was first mixed with 999 μL of PDE buffer (pH 7.0) to prepare a 20 μM solution. Then, 20 μL of PDE stock solution (1 mg/mL in H_2_O) was added. The reaction mixture was incubated at 37 °C for 1, 2, 4, 8, and 24 h. Emission was then observed with the UV transilluminator. Data are shown in Figure 8.

#### 4.4.4. ALP with Target Compounds under Basic Conditions

Substrate **18**, **19**, or **19-I_2_** (1 μL, 20 mM in DMSO) was first mixed with 999 μL of ALP buffer (pH 9.5) to prepare a 20 μM solution. Then, 20 μL of ALP stock solution (1 mg/mL in H_2_O) was added. The reaction mixture was incubated at 37 °C for 24 h. Substrates diluted with buffer (without addition of enzyme) served as negative control. Emission was then observed with the UV transilluminator. Data are shown in Figure 9A.

#### 4.4.5. ALP with Target Compounds under Physiological Conditions

Substrate **18**, **19**, or **19-I_2_** (1 μL, 20 mM in DMSO) was first mixed with 999 μL of 1× PBS buffer (pH 7.4) to prepare a 20 μM solution. Then, 20 μL of ALP stock solution (1 mg/mL in H_2_O) was added. The reaction mixture was incubated at 37 °C for 24 h. Substrates diluted with buffer (without addition of enzyme) served as negative control. Emission was then observed with the UV transilluminator. Data are shown in Figure 9B.

#### 4.4.6. PDE vs ALP with Target Compounds under Physiological Conditions–Timecourse

Substrate **18**, **19**, or **19-I_2_** (1 μL, 20 mM in DMSO) was first mixed with 999 μL of PDE buffer (pH 7.0) or 1× PBS buffer (pH 7.4) to prepare a 20 μM solution. Then, enzyme (PDE or ALP) was added to give a concentration of 100 nM. The reaction mixture was incubated at 37 °C for 1, 2, 8 and 24 h. Emission was then observed with the UV transilluminator. Data are shown in Figure A3.

## 5. Outlook

We have drawn on two disparate fields—biomedicine and materials chemistry—in an effort to design compounds for use in enzyme-triggered molecular brachytherapy. Much remains to be done. Some molecular designs that were attractive from an enzyme-cleavage standpoint, particularly the presence of the phosphodiester, proved deactivated toward subsequent iodination. The presence of an appended electron-rich aryloxy group did not give enhanced iodination, prompting use of an appended *p*-phenol motif. The latter design enables iodination at the last step of the synthesis. While many substances are now known to exhibit AIE [55], the aggregation (“A”) features of the HBT substrate are particularly attractive for amenability to enzymatic triggering and iodide incorporation. Further development of this approach requires adapting phosphodiesterase enzymes for the substrates in hand, as well as further alterations to impart diminished aqueous solubility following phosphodiester cleavage. While the work reported herein has focused on enzymatic release of compounds containing the HBT motif, a recent report illustrates the use of light to achieve a similar phenomenon upon exposure to hydrogen sulfide [56]. Together, the studies illustrate the utility of AIE chromogens in new domains.

## Figures and Tables

**Table 2:** Peak maxima of compounds in H_2_O containing 0.1% DMSO.

Compound	λ_abs_ (nm)	λ_exc_ (nm)	λ_em_ (nm)
**14**	334*^a^*	318	522
**15**	350 *^a^*	330	534
**18**	318	318	374, 384
**19**	330	330	480
**19-I_2_**	330	330	435

*^a^* In neat DMSO.

## Data Availability

All data are contained within the paper and Supplementary Materials.

## Supplementary Materials

The following supporting information can be downloaded at: https://www.mdpi.com/article/10.3390/molecules27248682/s1, NMR spectral data for all new compounds.

## Appendix A

Four experiments were carried out in support of the development of the HBT substrates for use in molecular brachytherapy. The experiments examine (1) tolerance toward polar organic–aqueous environments, (2) concentration dependence in polar media, (3) tolerance toward organic media of a wide range of polarity, and (4) differences in behavior toward two enzymes.

(1) The emission properties of **14** and **15** were examined in media composed of various ratios of water and DMSO. The spectra of **14** are shown in Figure A1 panel A. The spectra of **15** are shown in Figure A1 panel B. In both cases, a shift of the emission peak to shorter wavelength (enol form) was observed in media containing a higher percentage of DMSO. For both **14** and **15**, a shoulder ~460 nm was detected when the DMSO percentage was increased to 30%. The enol form (peak maximum ~390 nm for **14** and ~430 nm for **15**) is found to be the dominant isomer in pure DMSO. The samples were examined in an ultraviolet transilluminator. An AIE process is evident for **14** in media containing as much as ~50% DMSO (Figure A1 panel C) and for **15** in media containing as much as ~40% DMSO (Figure A1 panel D).

In summary, the AIE process was resilient toward substantial quantities of the polar organic solvent DMSO.

(2) The concentration dependence of the emission spectra also was examined. Due to the low solubility of **14** and **15**, the emission spectra were acquired in H_2_O containing 20% DMSO. At a concentration of 20 μM, **14** and **15** showed an intense emission peak around 530 nm, which can be assigned to the keto form within aggregates. With lower concentrations (2 or 0.2 μM), a shoulder around 460 nm was revealed, which might be due to unassociated molecules that engage in hydrogen bonding to solvent molecules. The concentration-dependent spectra are displayed for **14** (Figure A1 panel E) and **15** (Figure A1 panel F).

In summary, the AIE effect was diminished, at least under the aqueous DMSO conditions examined here, upon use of concentrations at or below 2 μM.

(3) The emission spectra of HBT derivatives have been shown to be strongly solvent-dependent [53]. Compounds **14** and **15** exhibited similar properties across different solvents (Figure A2, panels A and B, respectively). The enol form (shorter wavelength) is dominant in DMSO, MeCN, MeOH, and THF solutions, while the keto form (longer wavelength) is obtained in dichloromethane (DCM), hexanes, and aqueous solutions. Among all the organic solvents, the emission peak maximum of **14** and **15** shifted to the characteristic enol emission peak with increasing solvent polarity. Unlike **15**, compound **14** in DMSO and MeOH solutions also exhibited a second intense peak at ~460 nm, which is at shorter wavelength than that of the keto peak in other solutions. This second intense peak has been reported to derive from the stacking of the enol form of HBT causing a decrease in the transition energy [53]. In summary, the AIE effect of **14** and **15**, at least with regards to formation of visible precipitates, is observed only in water.

(4) A comparison of the enzymatic treatment (PDE versus ALP) of three target substrates (**18**, **19**, and **19-I_2_**) under physiological condition (pH ~7, 37 °C) was performed. The results are shown in Figure A3. After 1 h incubation, emissive particles (**18** and **19**) and weak emission (**19-I_2_**) were observed when treated with PDE, while an increase in emission was only observed for **19** upon treatment with ALP. After 24 h incubation, emissive particles were observed for **18** and **19** under PDE treatment. No obvious precipitate was observed with ALP treatment. Substrate **19-I_2_** under the two conditions did not show obvious differences from the other two substrates.

In summary, the enzyme PDE cleaves **18** and **19** more extensively to form the products that exhibit AIE than is observed with the enzyme ALP. No significant difference was observed between the two enzymes for the substrate **19-I_2_**.
