# Supplementary material for: Tailoring the AIE Chromogen 2-(2-Hydroxyphenyl)benzothiazole for Use in Enzyme-Triggered Molecular Brachytherapy
Source: Molecules. 2022 Dec 8;27(24):8682. doi: 10.3390/molecules27248682 (PMC9786593; doi:10.3390/molecules27248682)

**Tailoring the AIE Chromogen 2-(2-Hydroxyphenyl)benzothiazole for Use in Enzyme-Triggered Molecular Brachytherapy**

Zhiyuan Wu, Jinghuai Dou, Kathy-Uyen Nguyen, Jayden C. Eppley, Kittipan Siwawannapong, Yunlong Zhang, and Jonathan S. Lindsey*

Department of Chemistry, North Carolina State University, Raleigh, North Carolina 27695-8204, USA

* Correspondence: jlindsey@ncsu.edu (J.S.L.); Tel.: +1-919-515-6406

**Contents** **Pages**

$^{1}H$, $^{13}C$, and $^{31}P$ NMR spectra S2–S52

**1-I**: $^{1}H$ NMR ($CDCl_3$, 600 MHz)

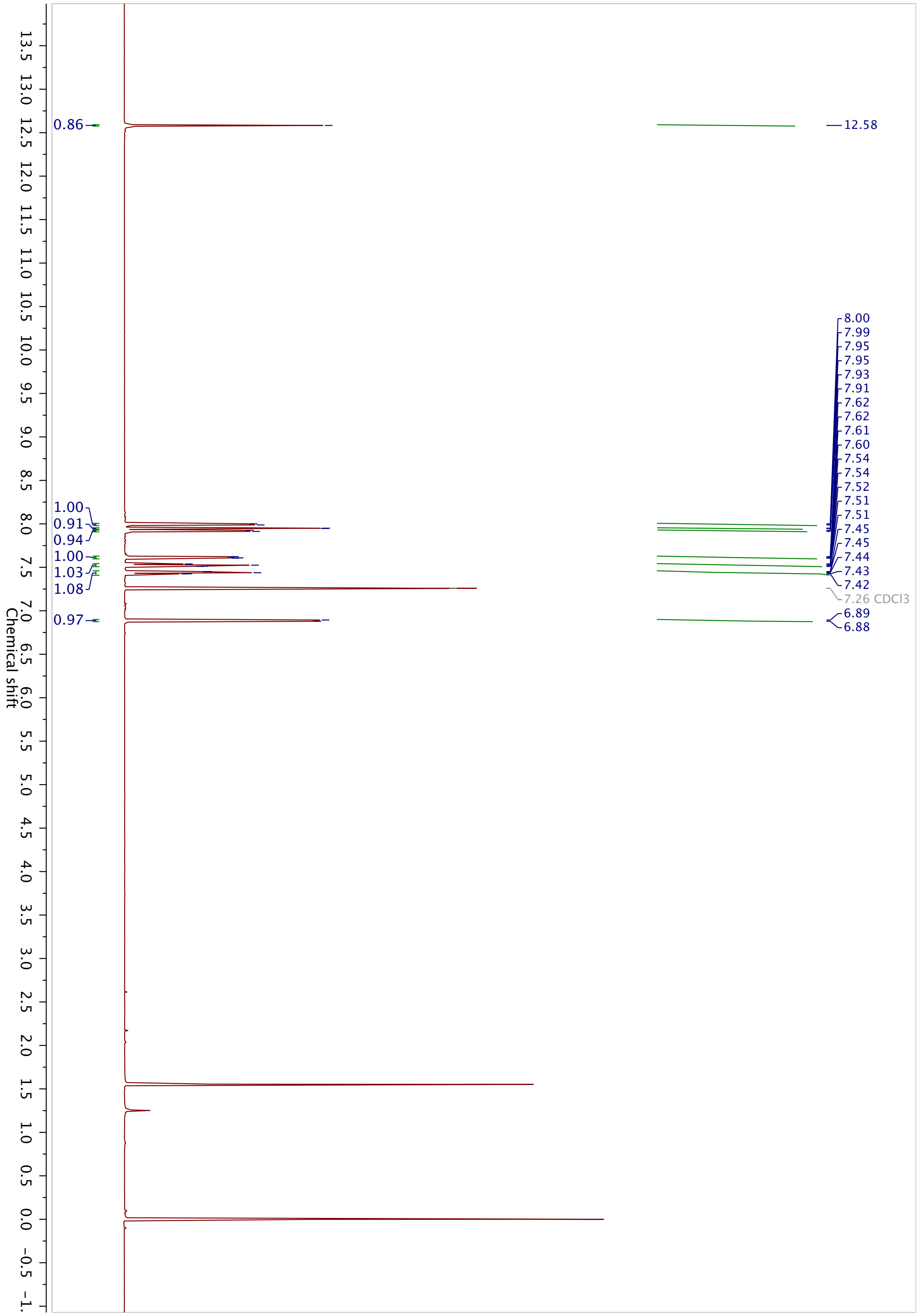

**1-I**: $^{13}$C NMR ($CDCl_3$, 150 MHz)

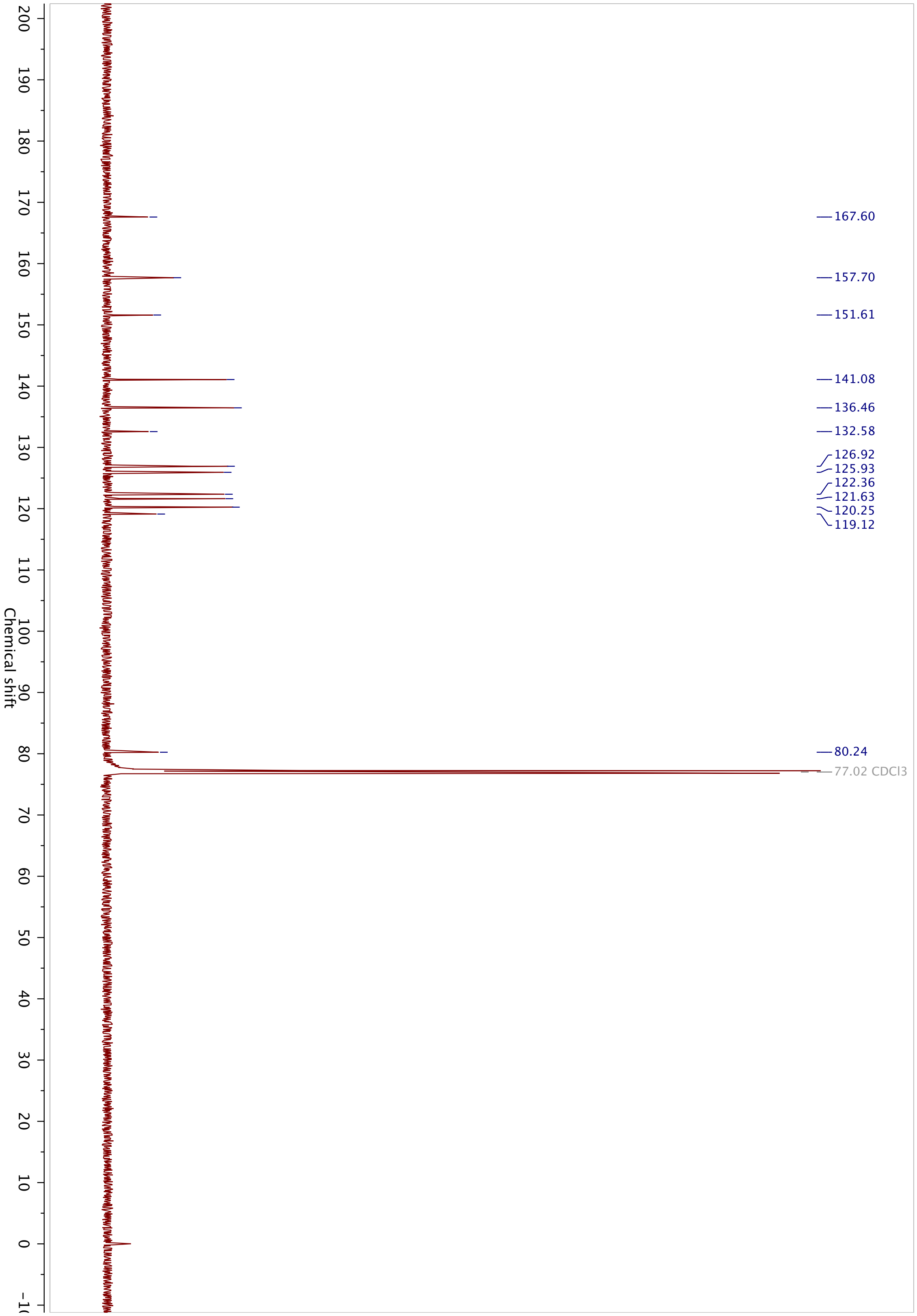

**1-I₂**: $^{1}$H NMR ($CDCl_3$, 500 MHz)

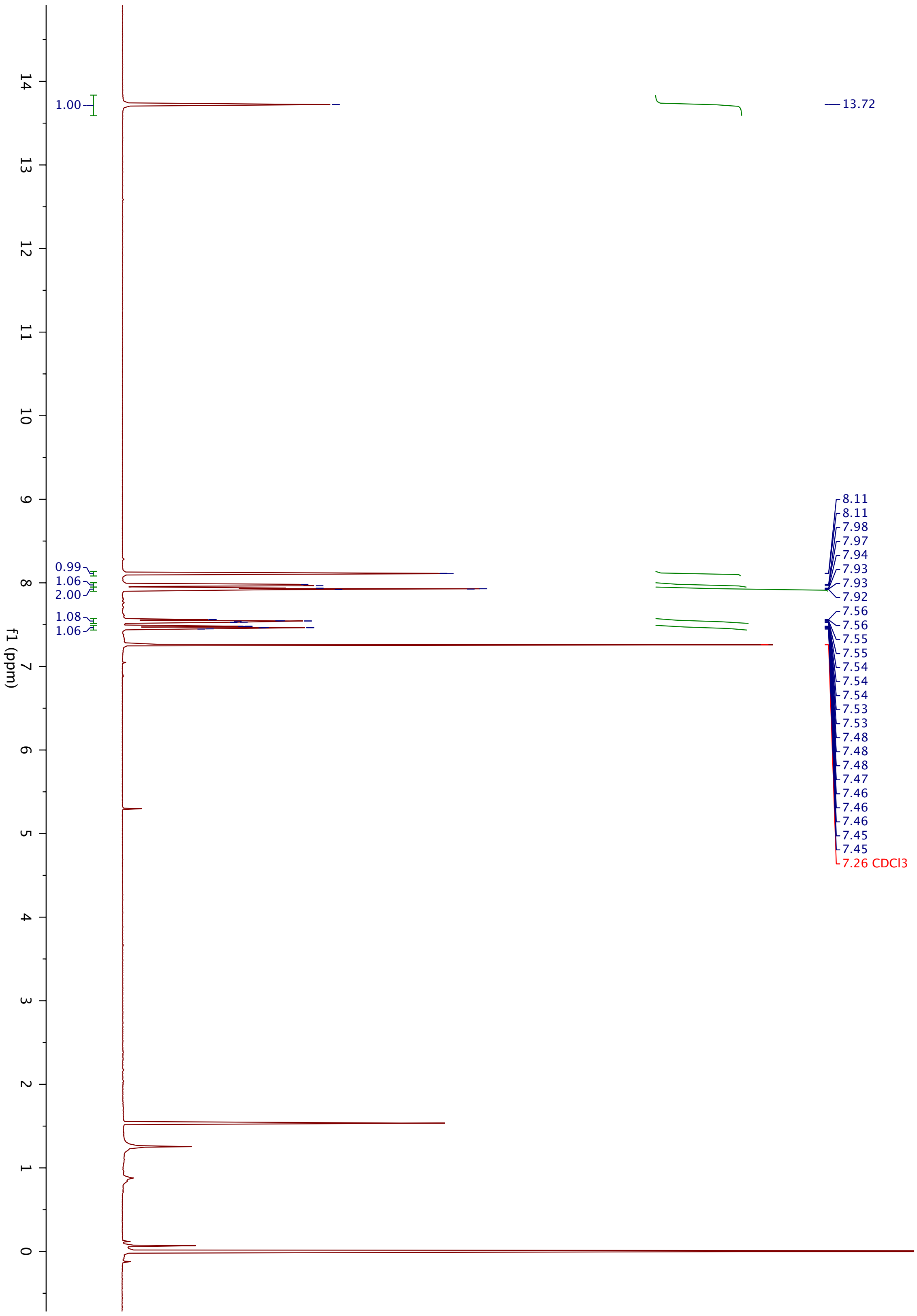

**1-I$_2$**: $^1$H NMR ($CDCl_3$, 125 MHz)

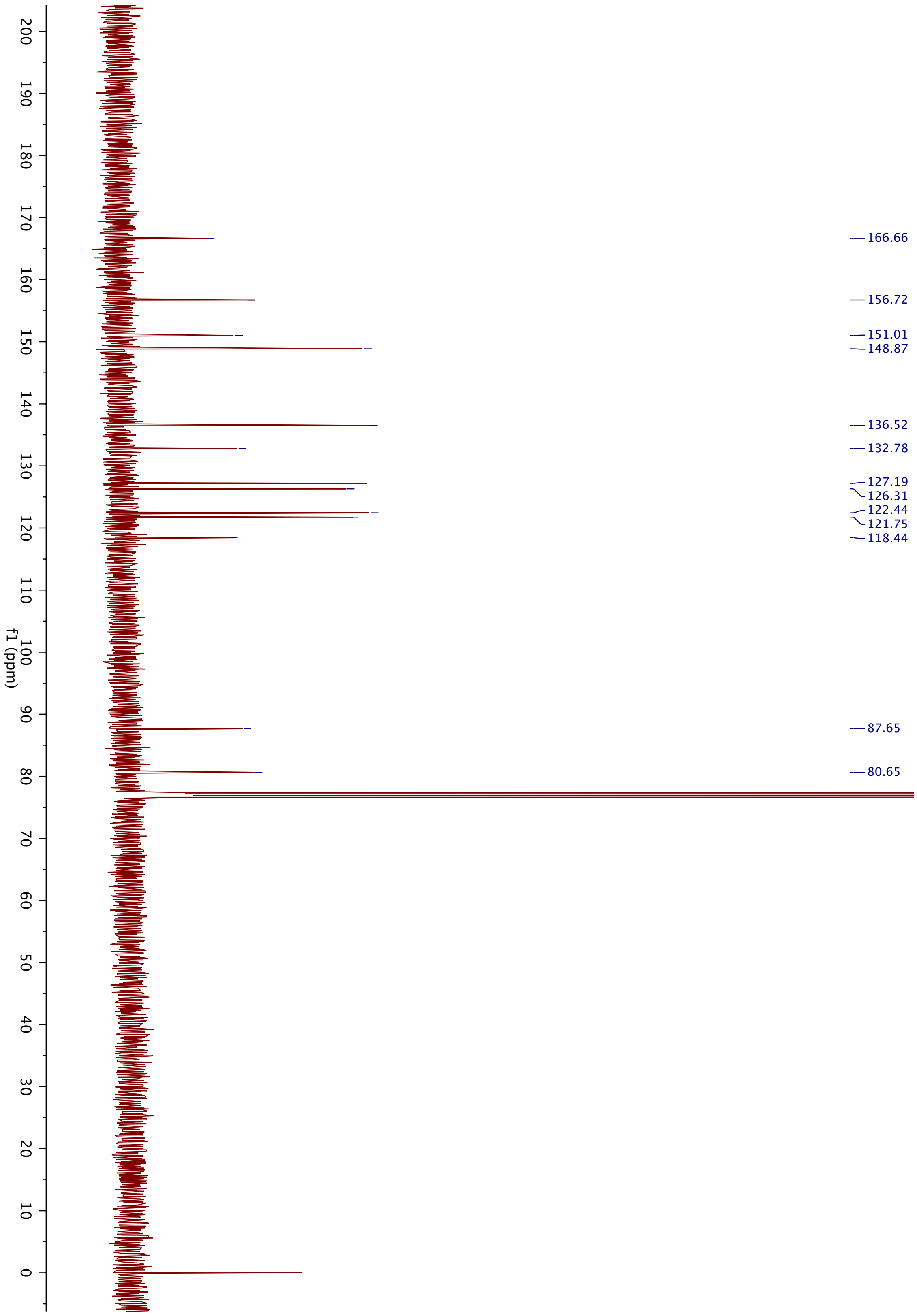

**2-Br**: $^1$H NMR ($CD_3OD$, 500 MHz)

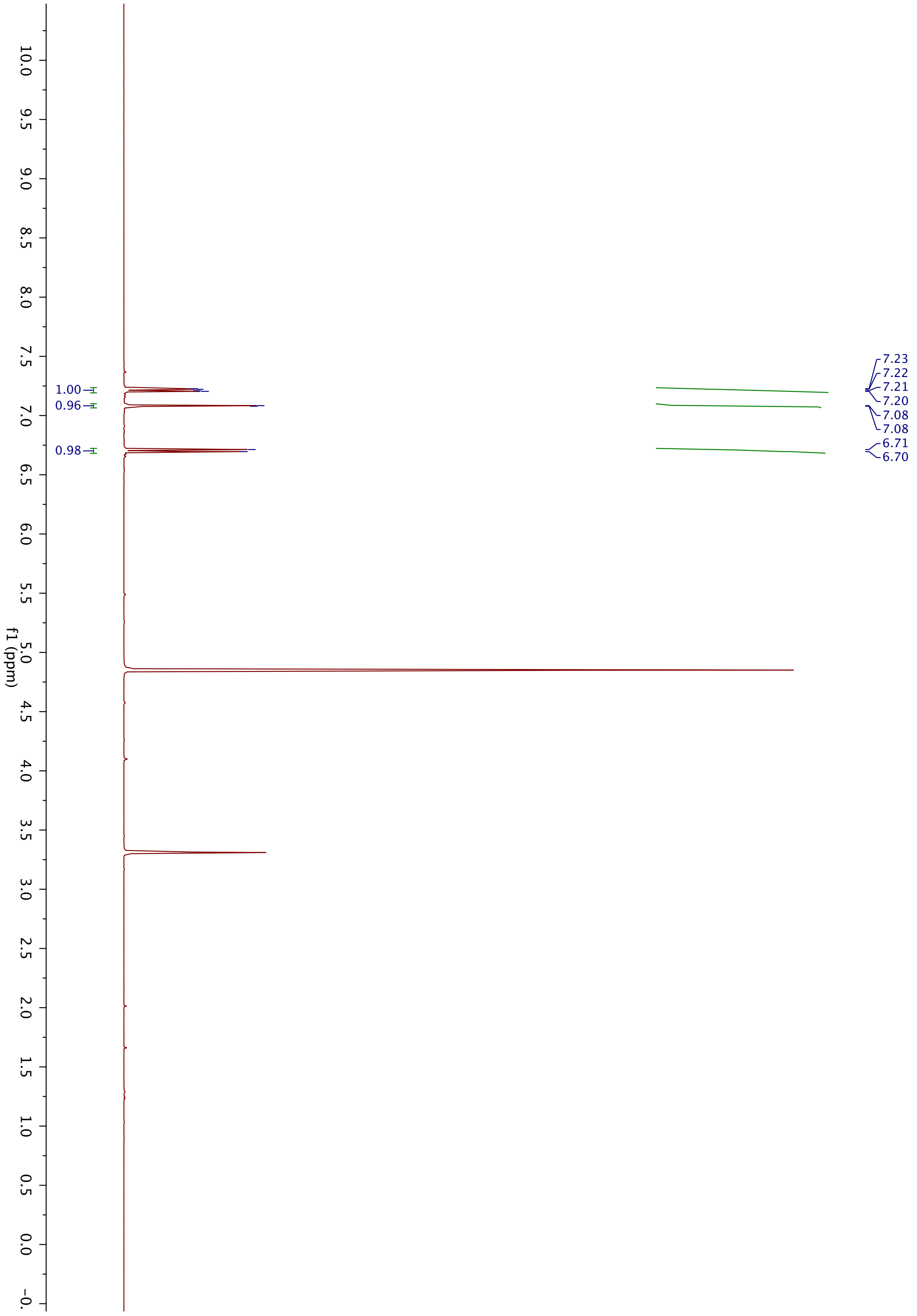

**2-Br**: $^{13}C$ NMR ($CD_3OD$, 125 MHz)

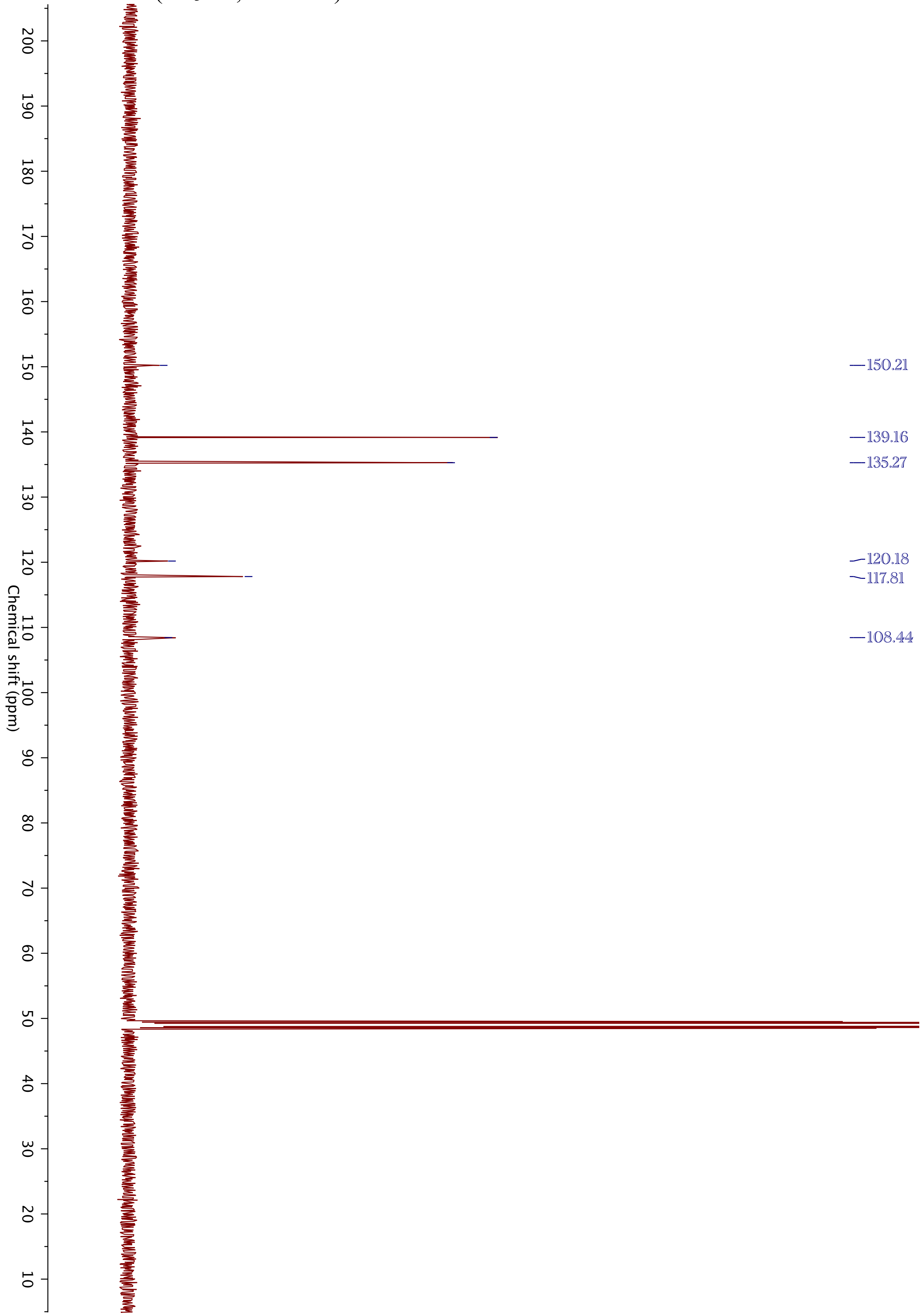

**6-I**: $^{31}$P NMR ($CDCl_3$, 240 MHz)

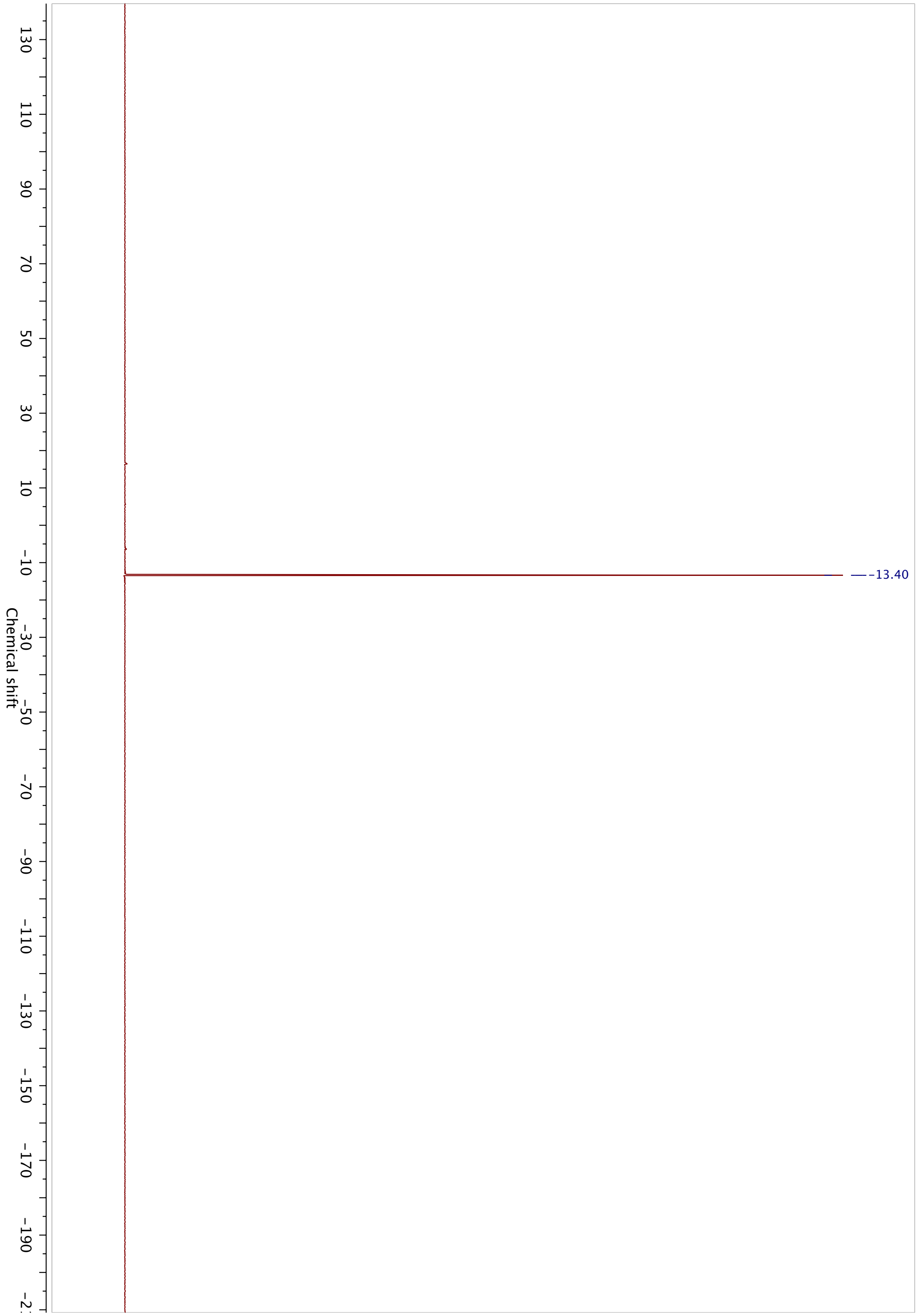

**6-I**: $^{1}$H NMR ($CDCl_3$, 600 MHz)

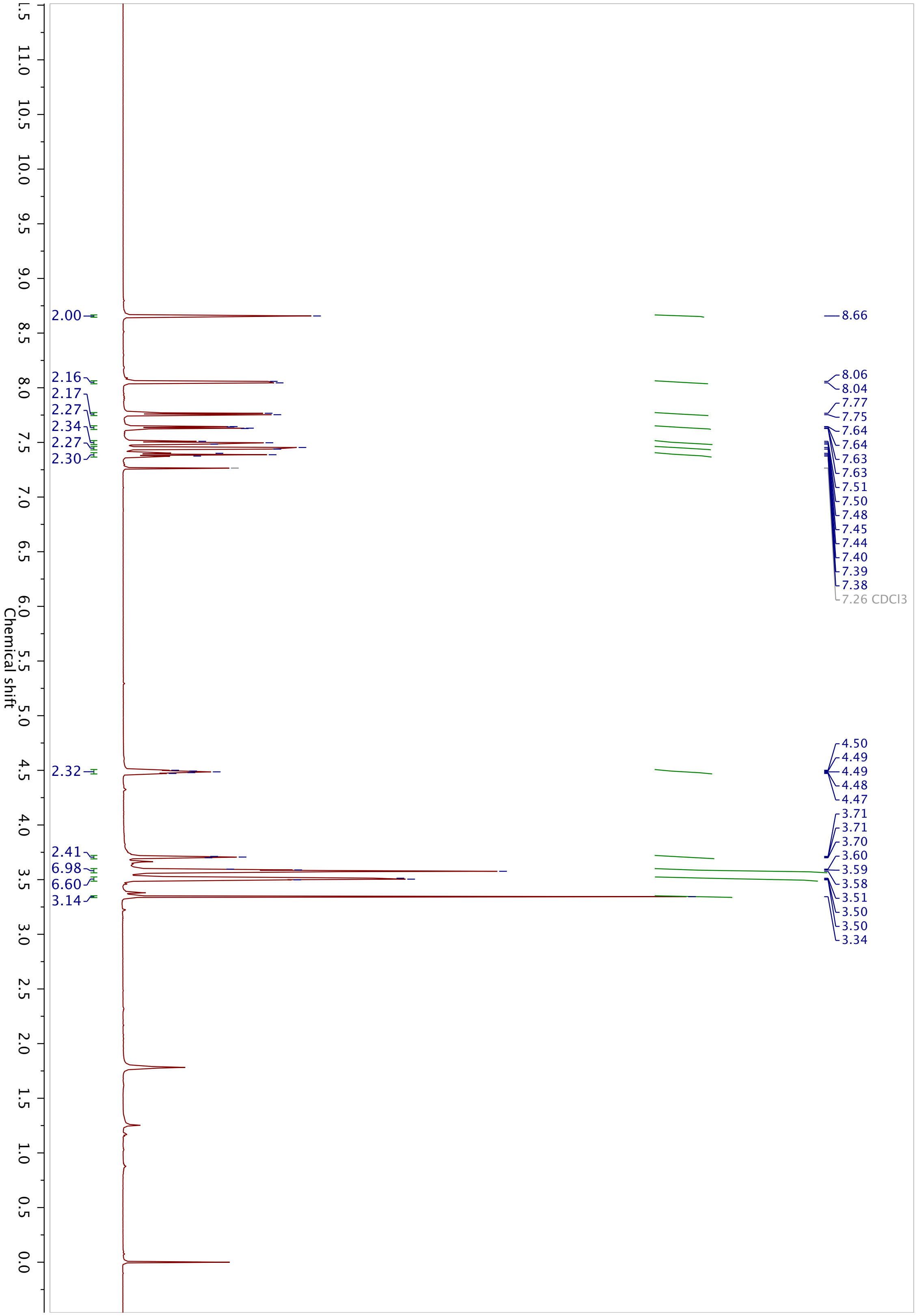

**6-I**: $^{13}C$ NMR ($CDCl_3$, 150 MHz)

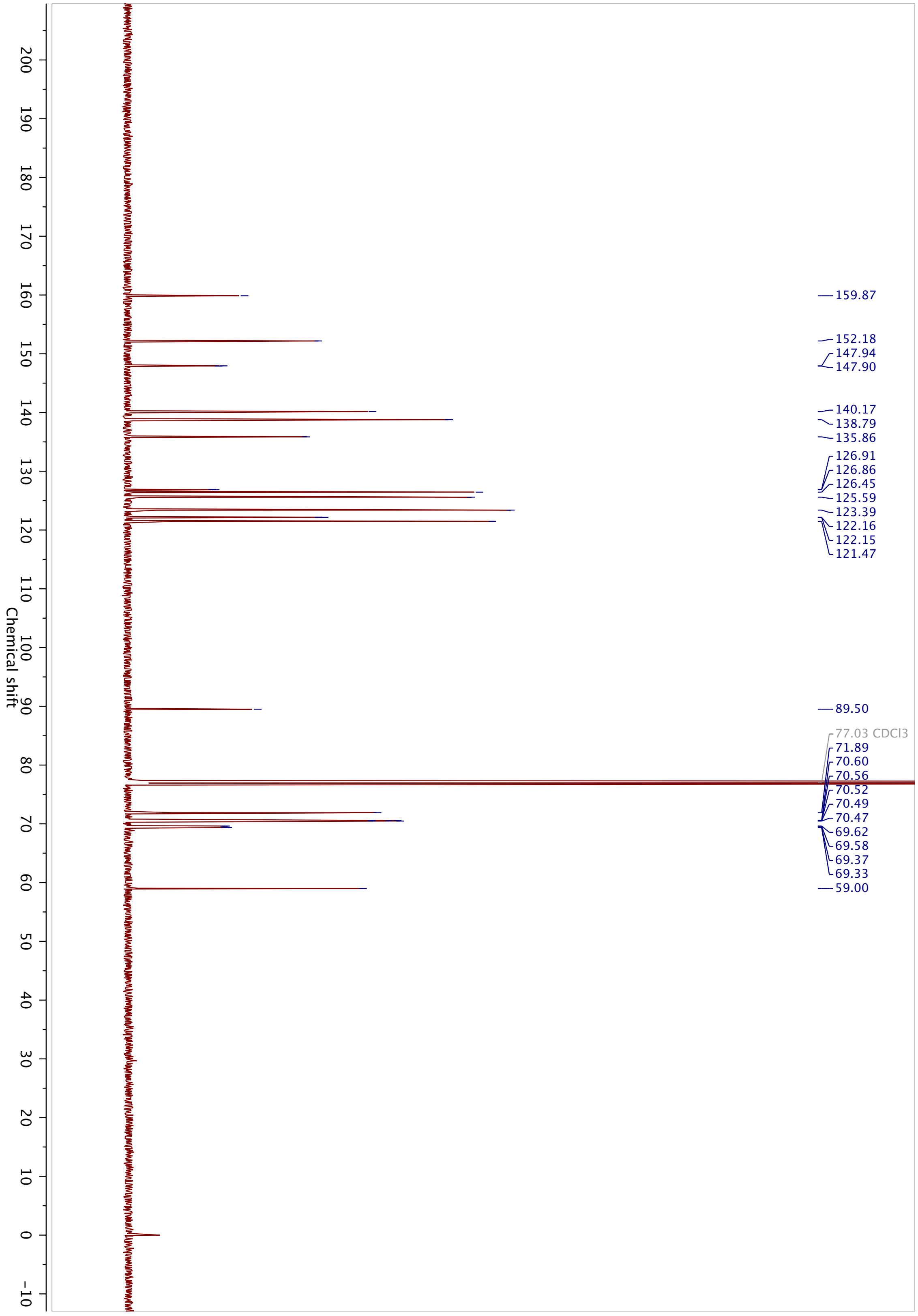

**7-I**: $^{31}$P NMR ($CDCl_3$, 240 MHz)

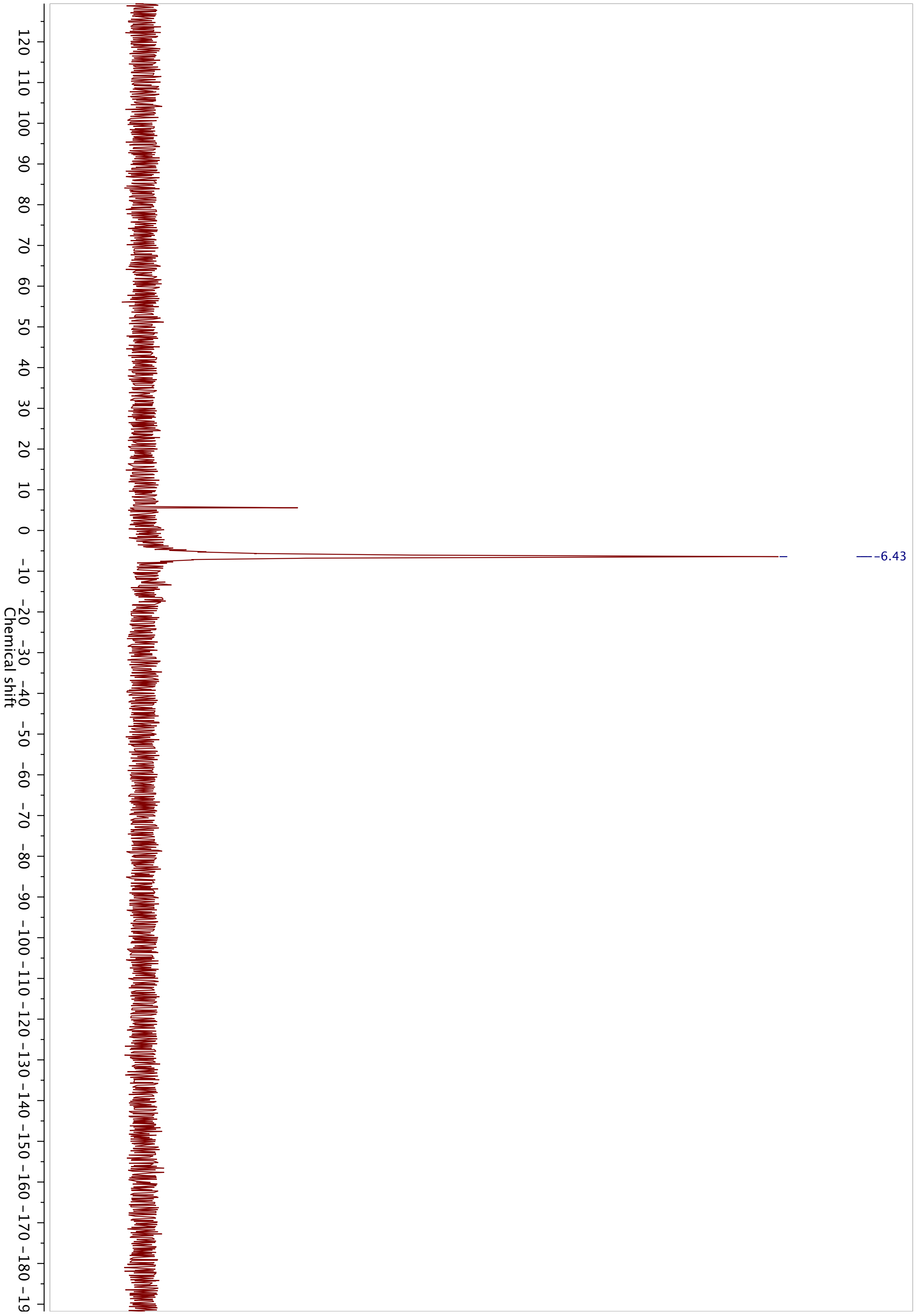

**7-I**: $^1$H NMR ($CDCl_3$, 600 MHz)

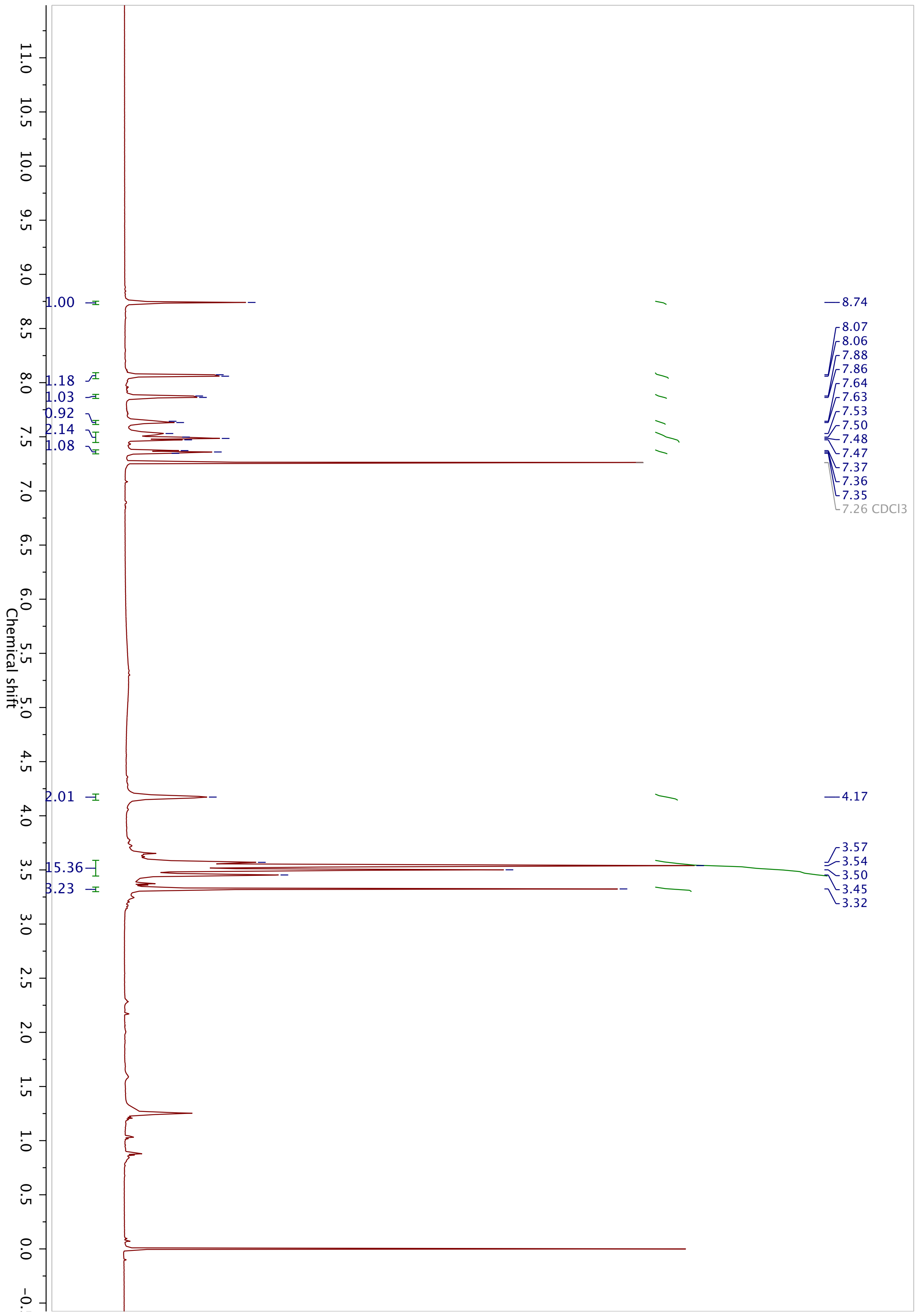

**7-I**: $^{13}$C NMR ($CDCl_3$, 150 MHz)

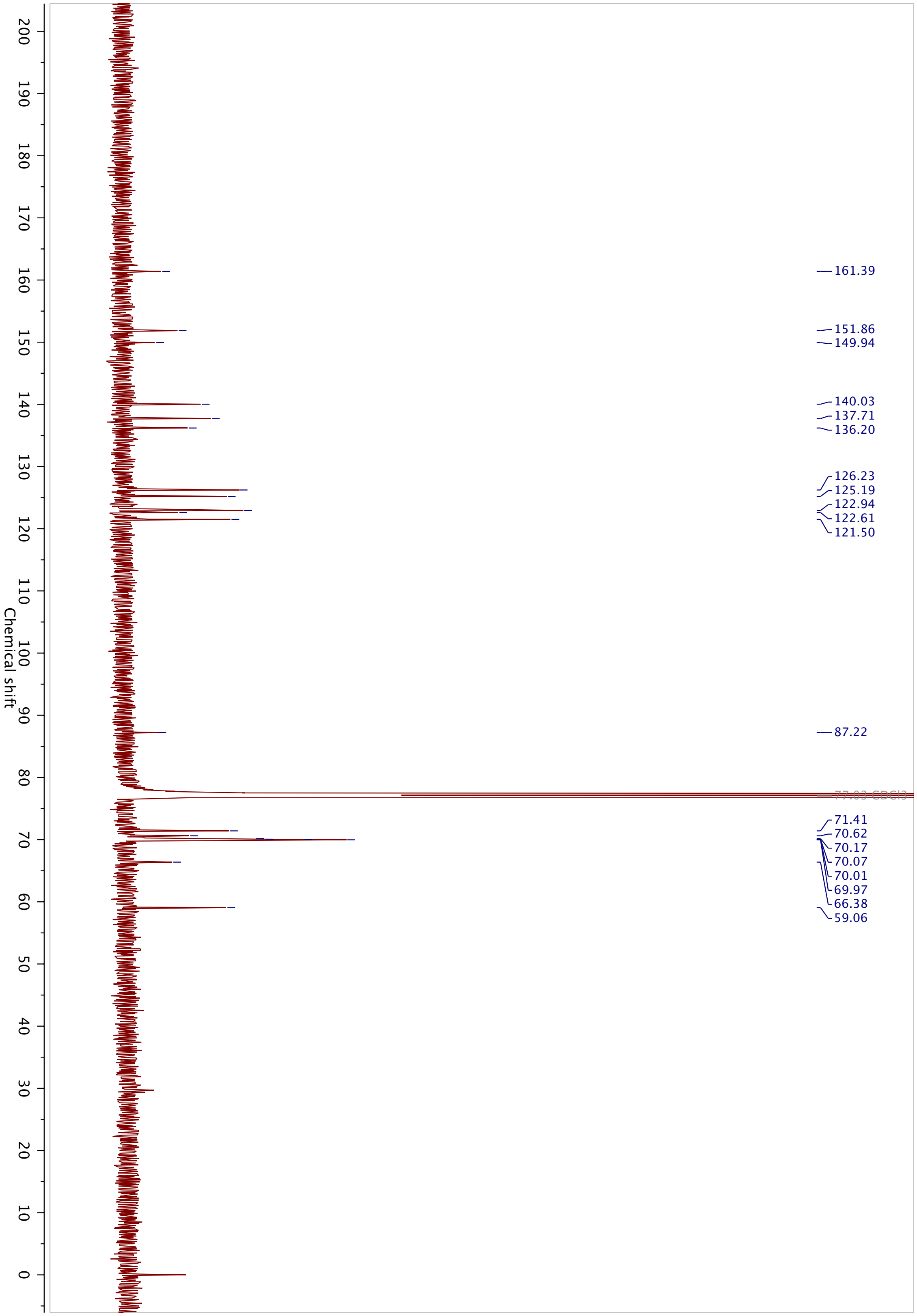

**9a**: $^{1}$H NMR ($CDCl_3$, 600 MHz)

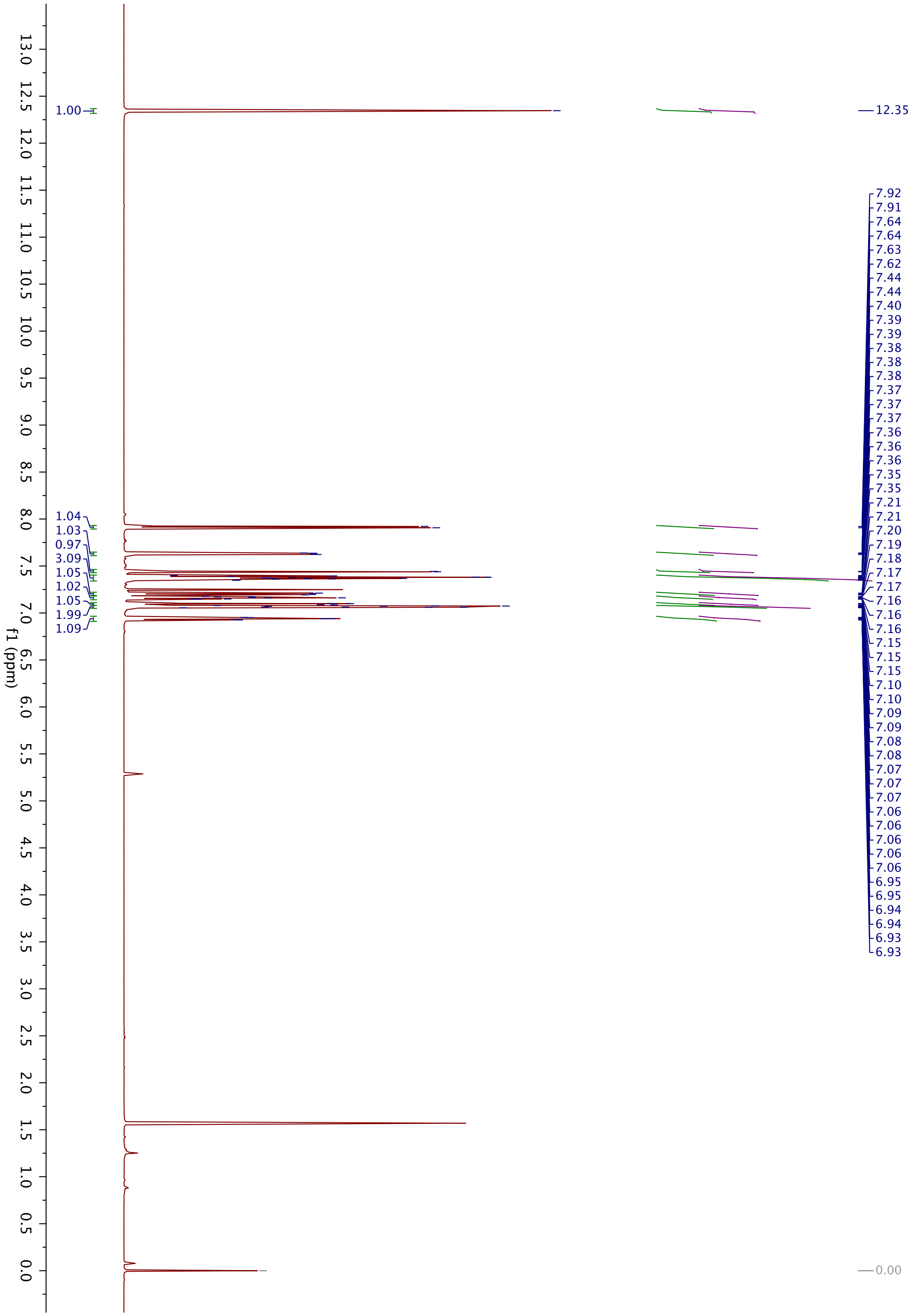

**9a**: $^{13}C$ NMR ($CDCl_3$, 150 MHz)

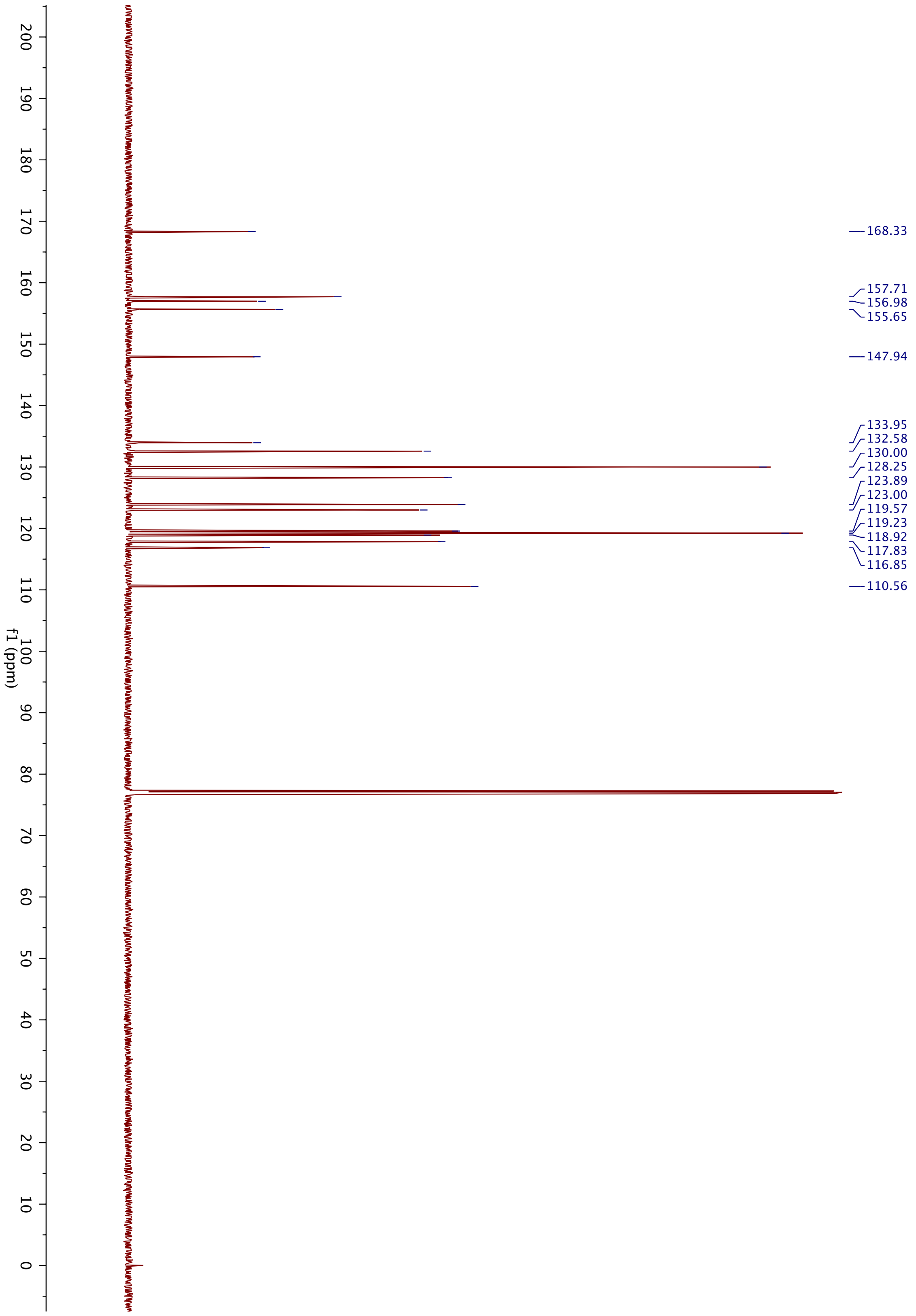

**9b**: $^1$H NMR ($CDCl_3$, 600 MHz)

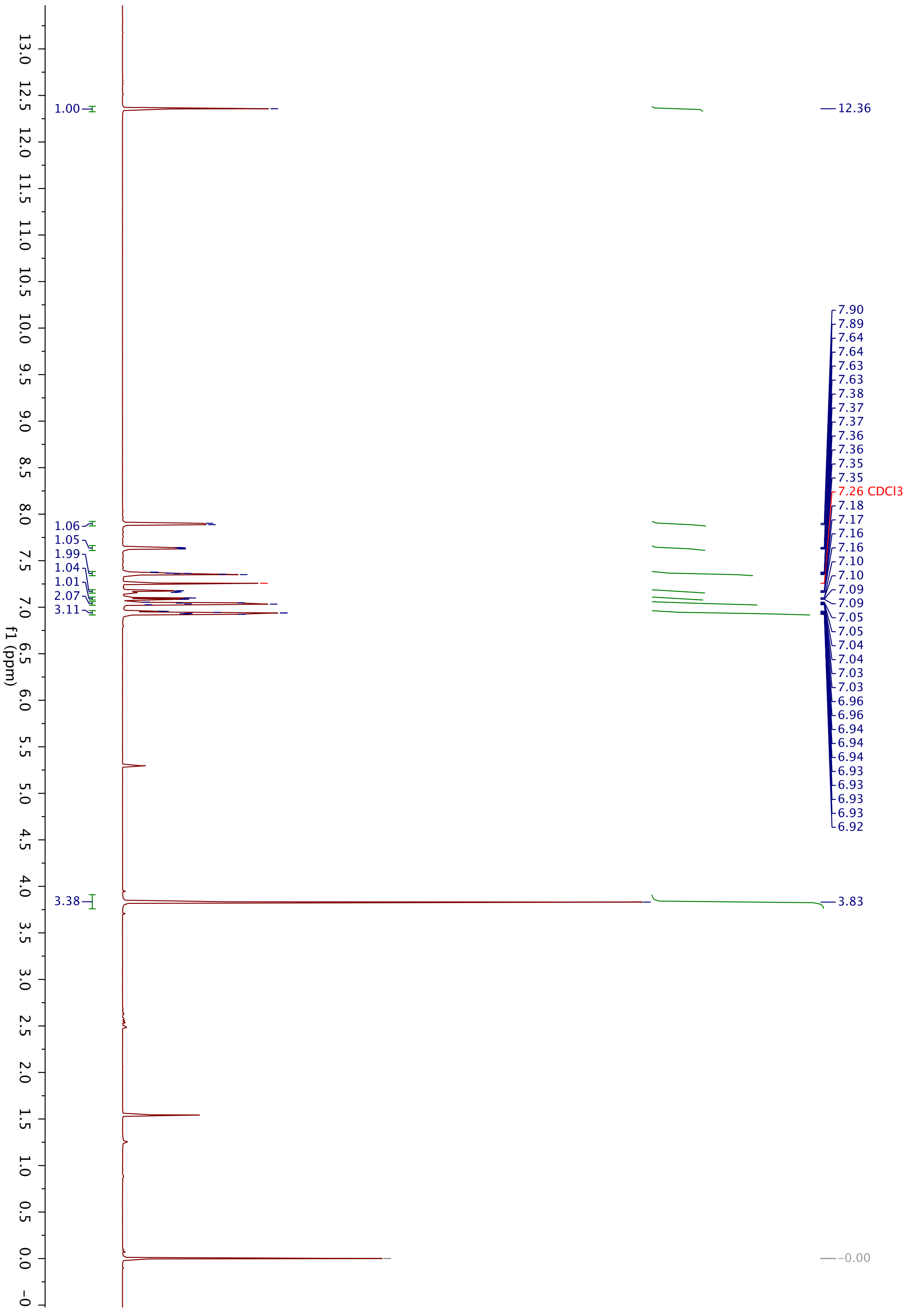

**9b**: $^{13}$C NMR ($CDCl_3$, 75 MHz)

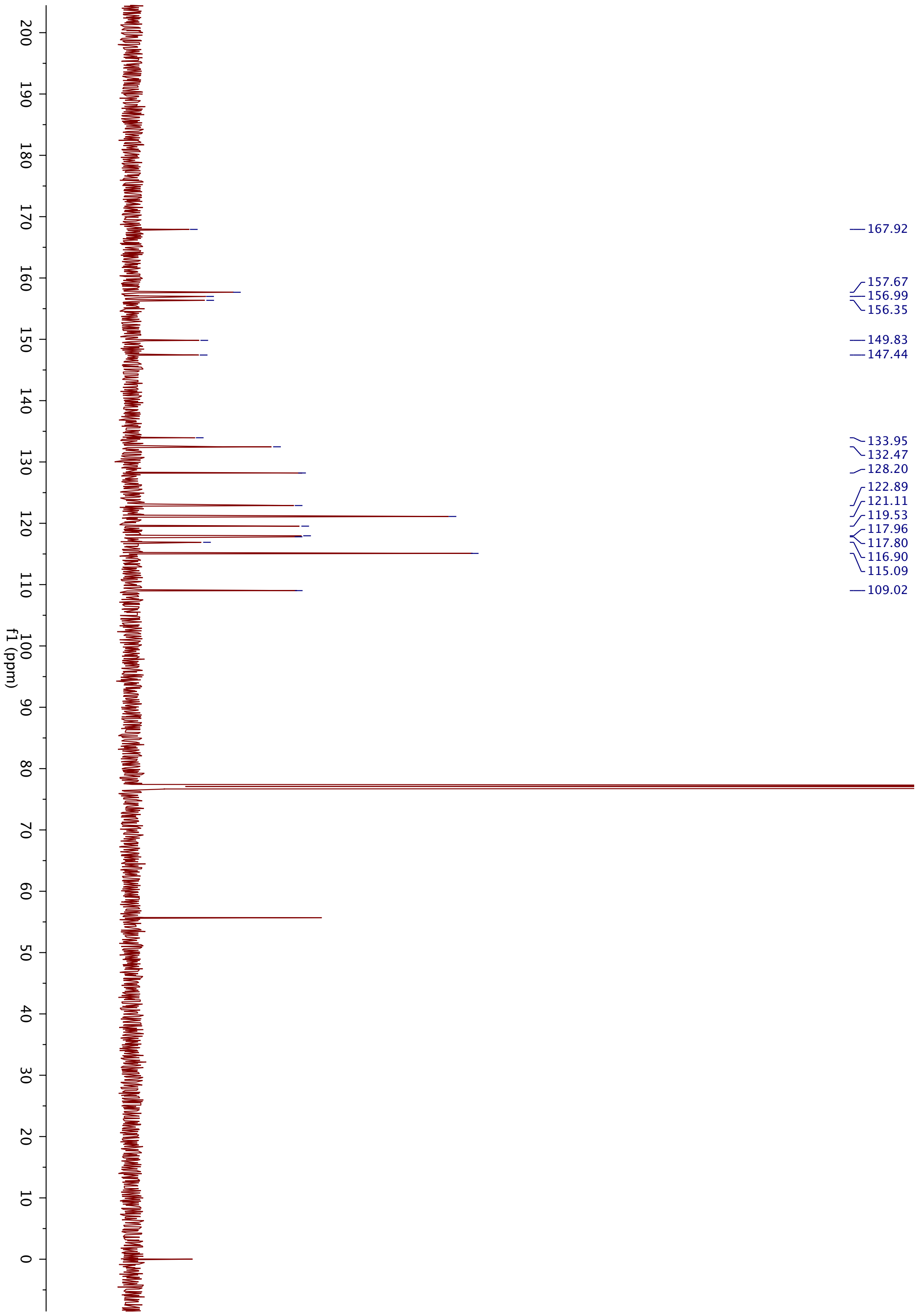

**9c**: $^{1}$H NMR ($CDCl_3$, 500 MHz)

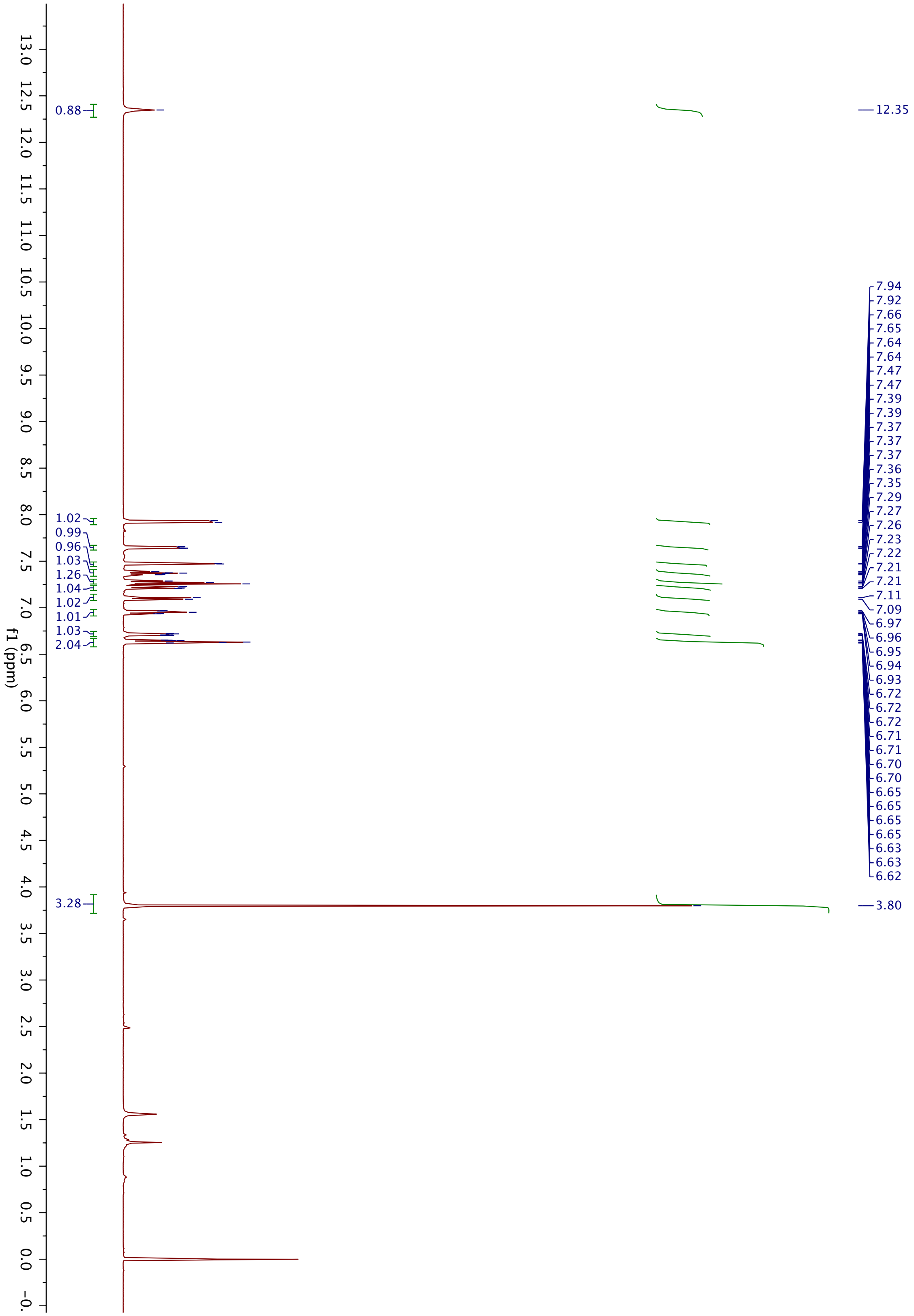

**9c**: $^{13}C$ NMR ($CDCl_3$, 125 MHz)

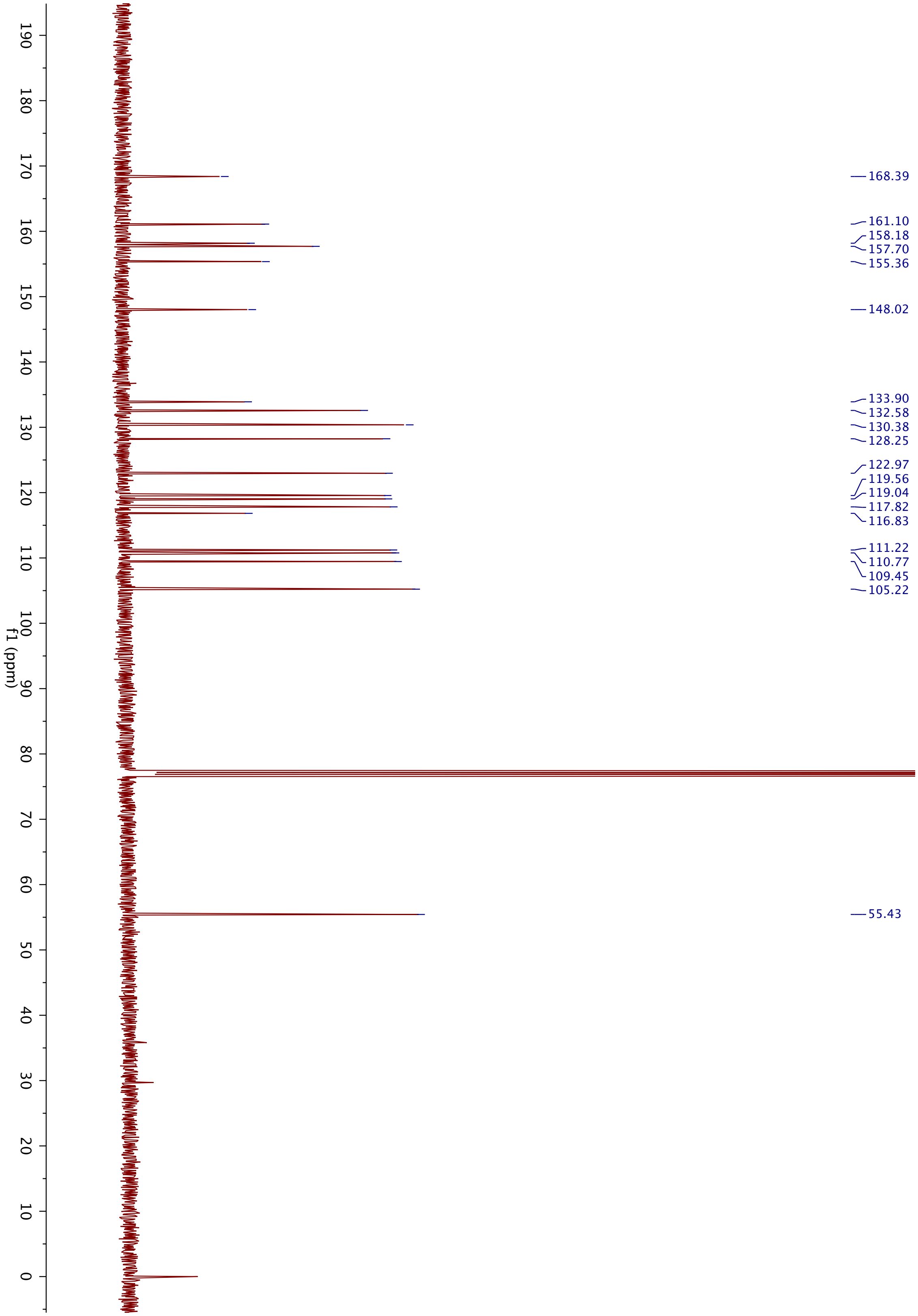

**10a**: $^{31}P$ NMR ($CDCl_3$, 160MHz)

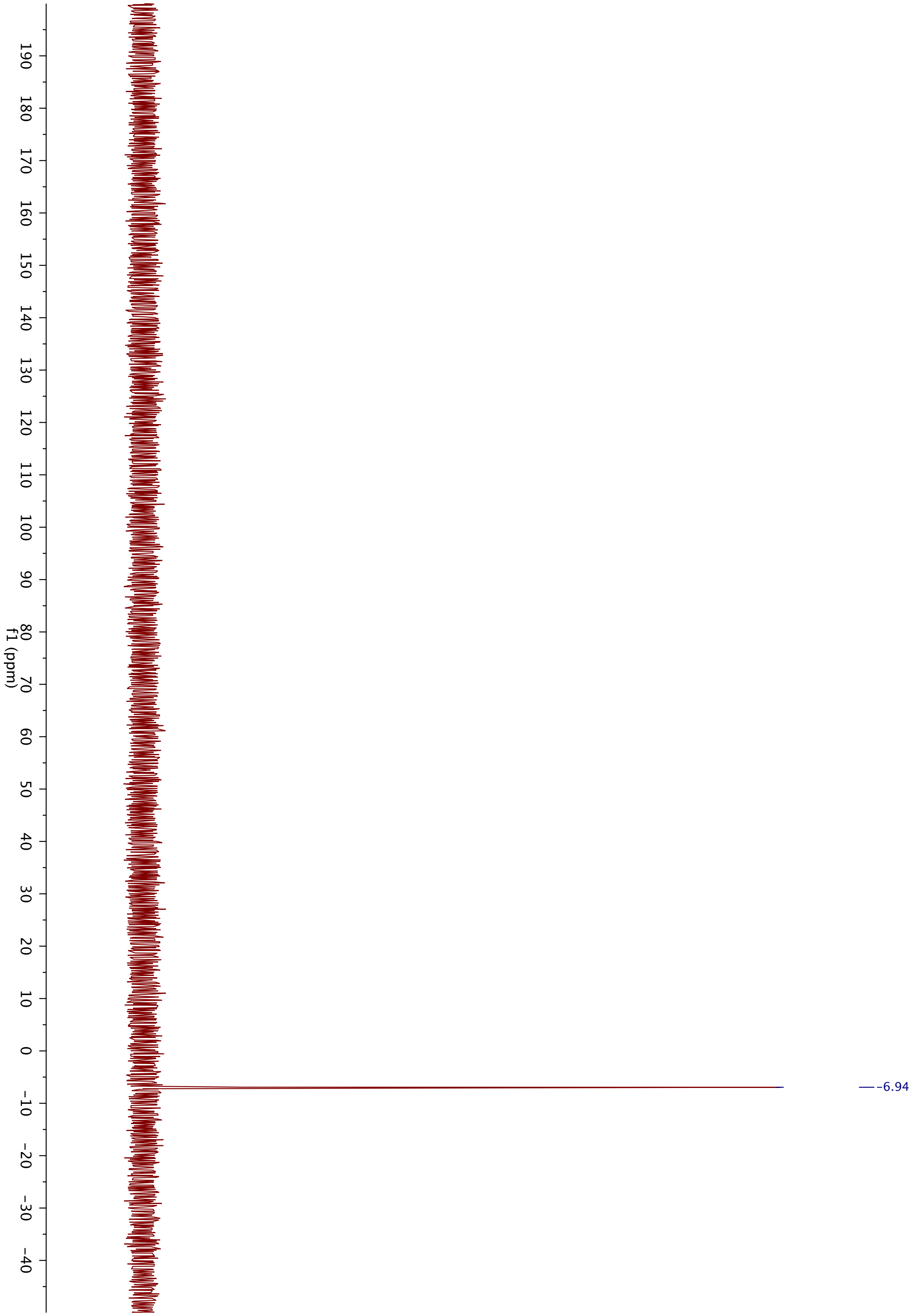

**10a**: $^{1}$H NMR ($CDCl_3$, 600 MHz)

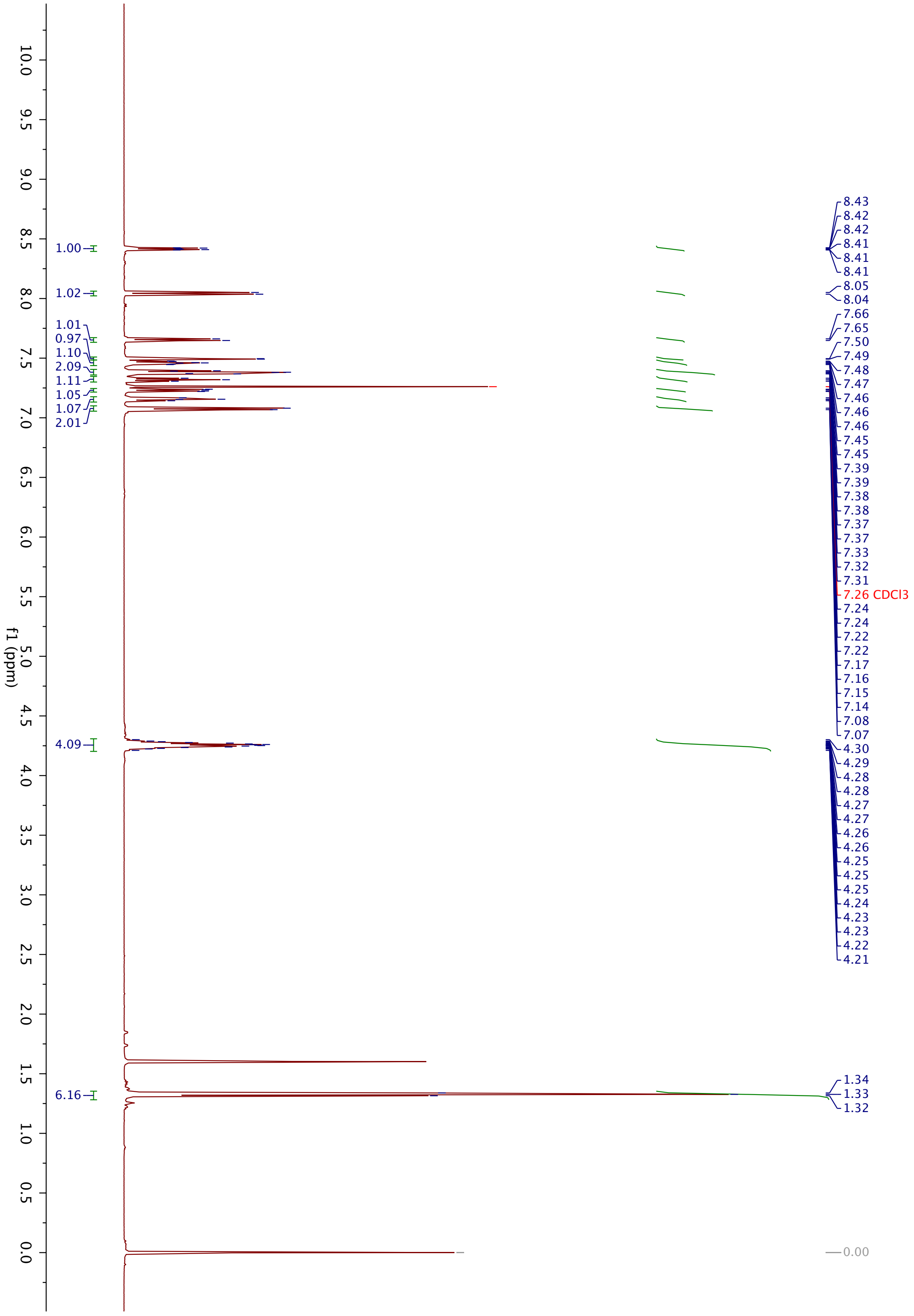

**10a**: $^{13}$C NMR (CDCl$_3$, 150 MHz)

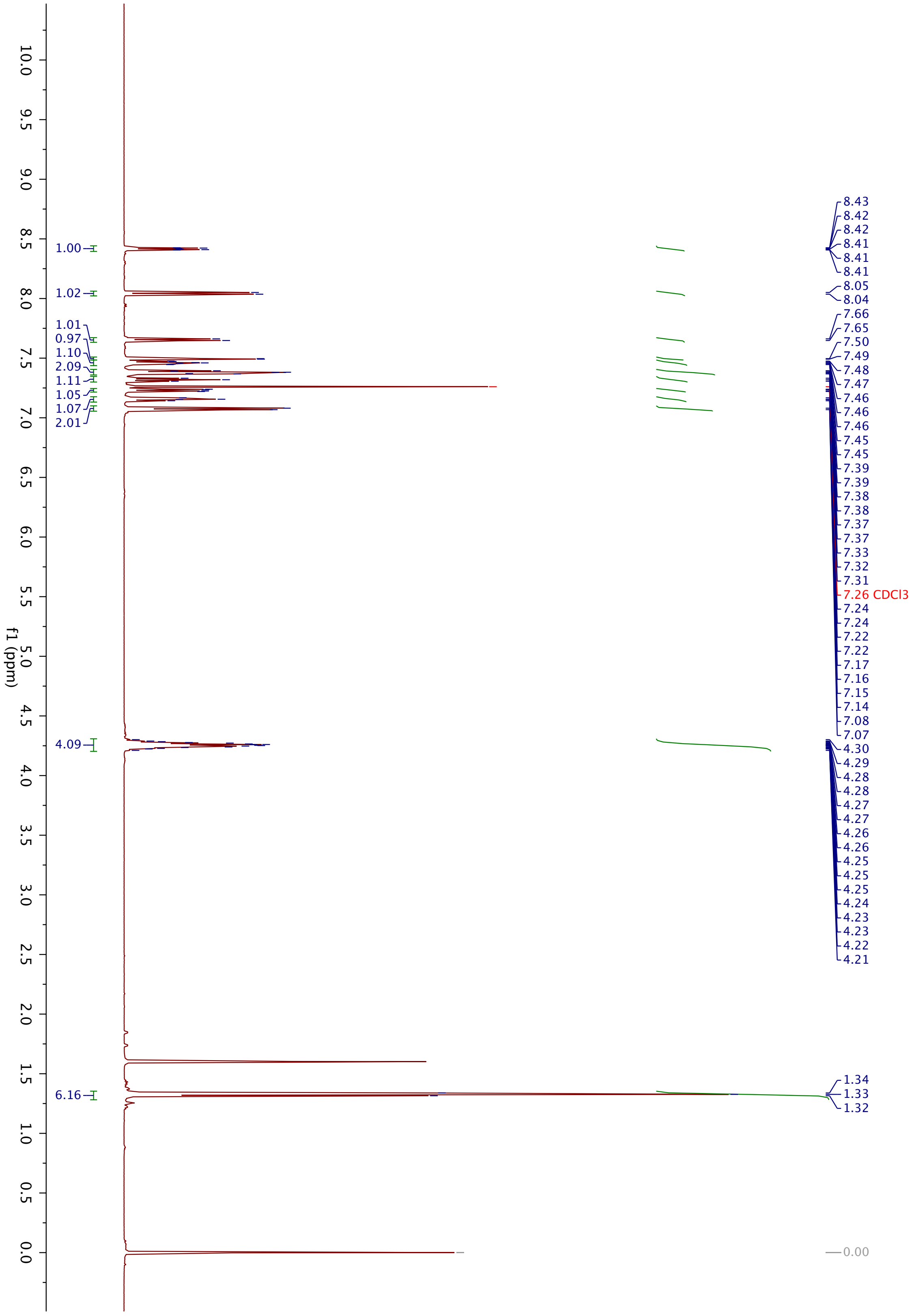

**10b**: $^{31}$P NMR ($CDCl_3$, 160MHz)

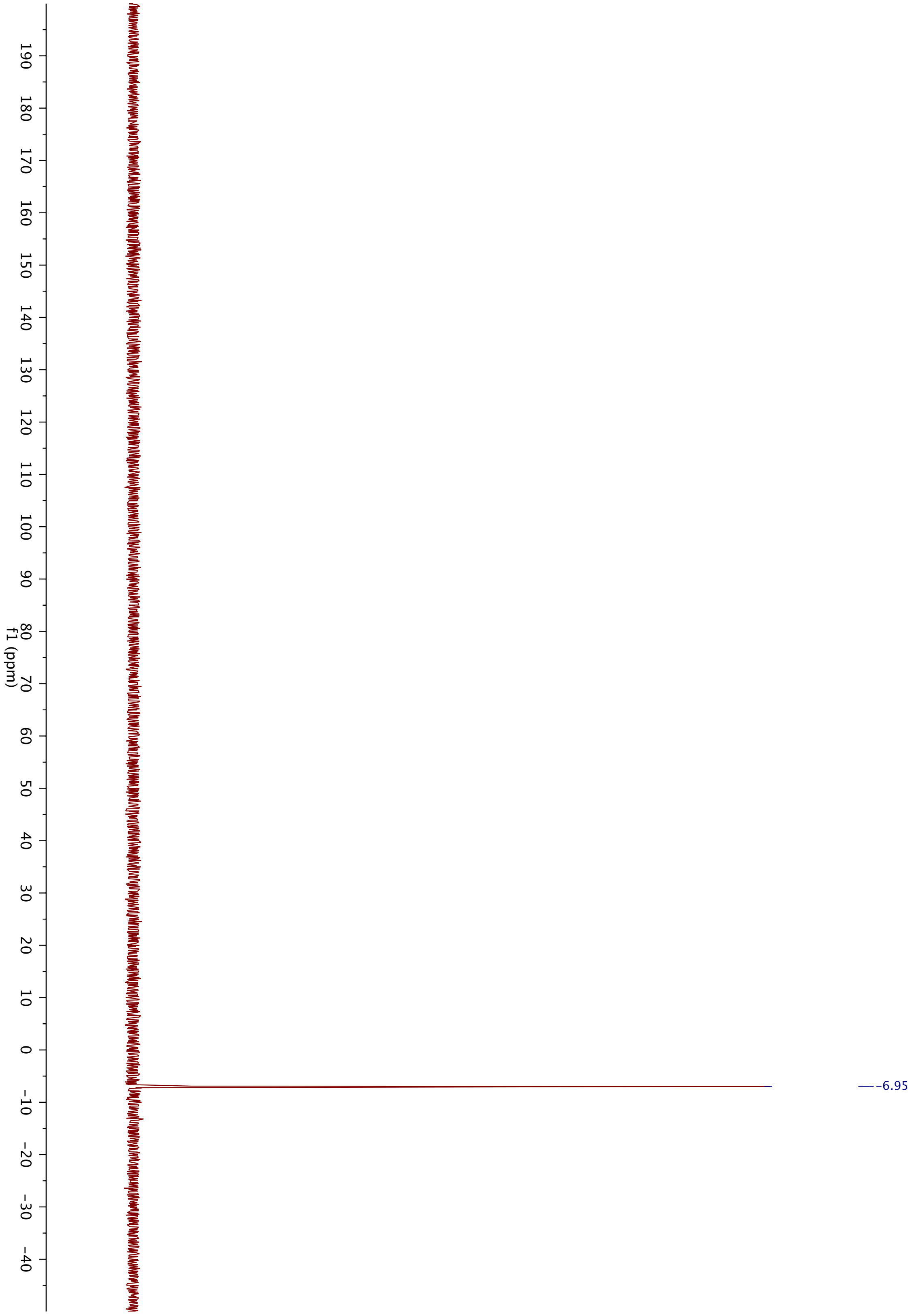

**10b**: $^{1}H$ NMR ($CDCl_3$, 600 MHz)

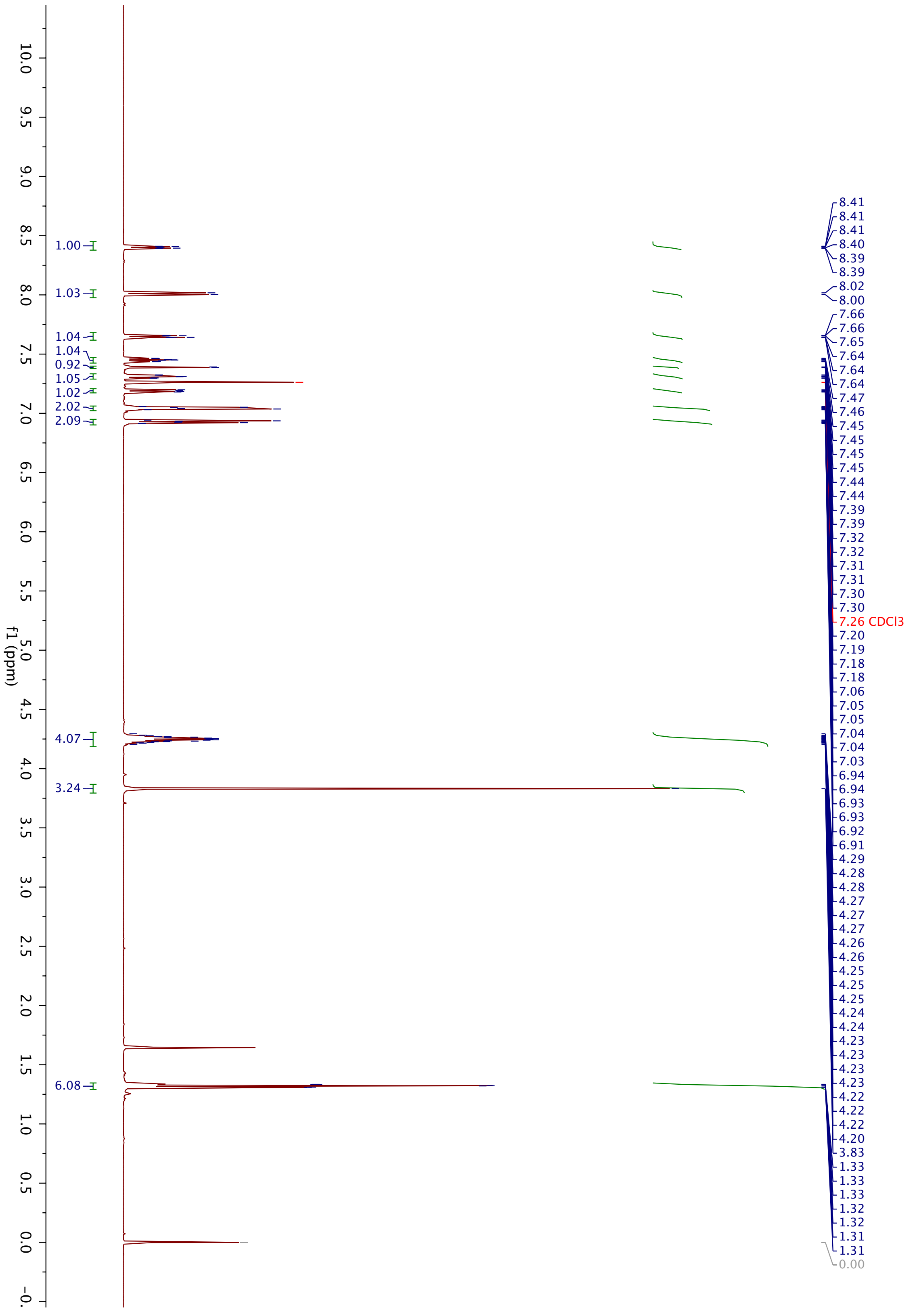

**10b**: $^{13}$C NMR ($CDCl_3$, 150 MHz)

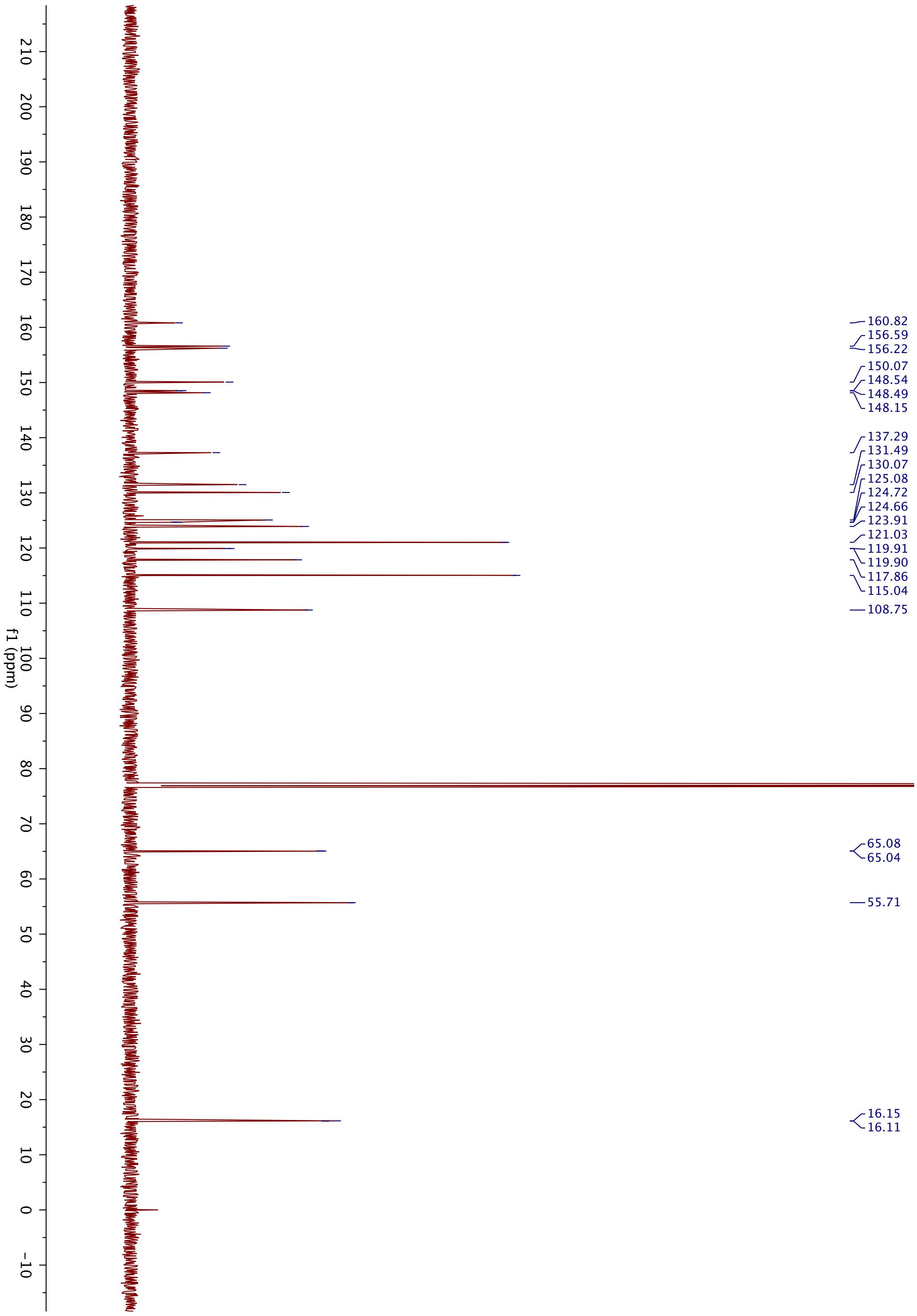

**10c**: $^{31}P$ NMR ($CDCl_3$, 160MHz)

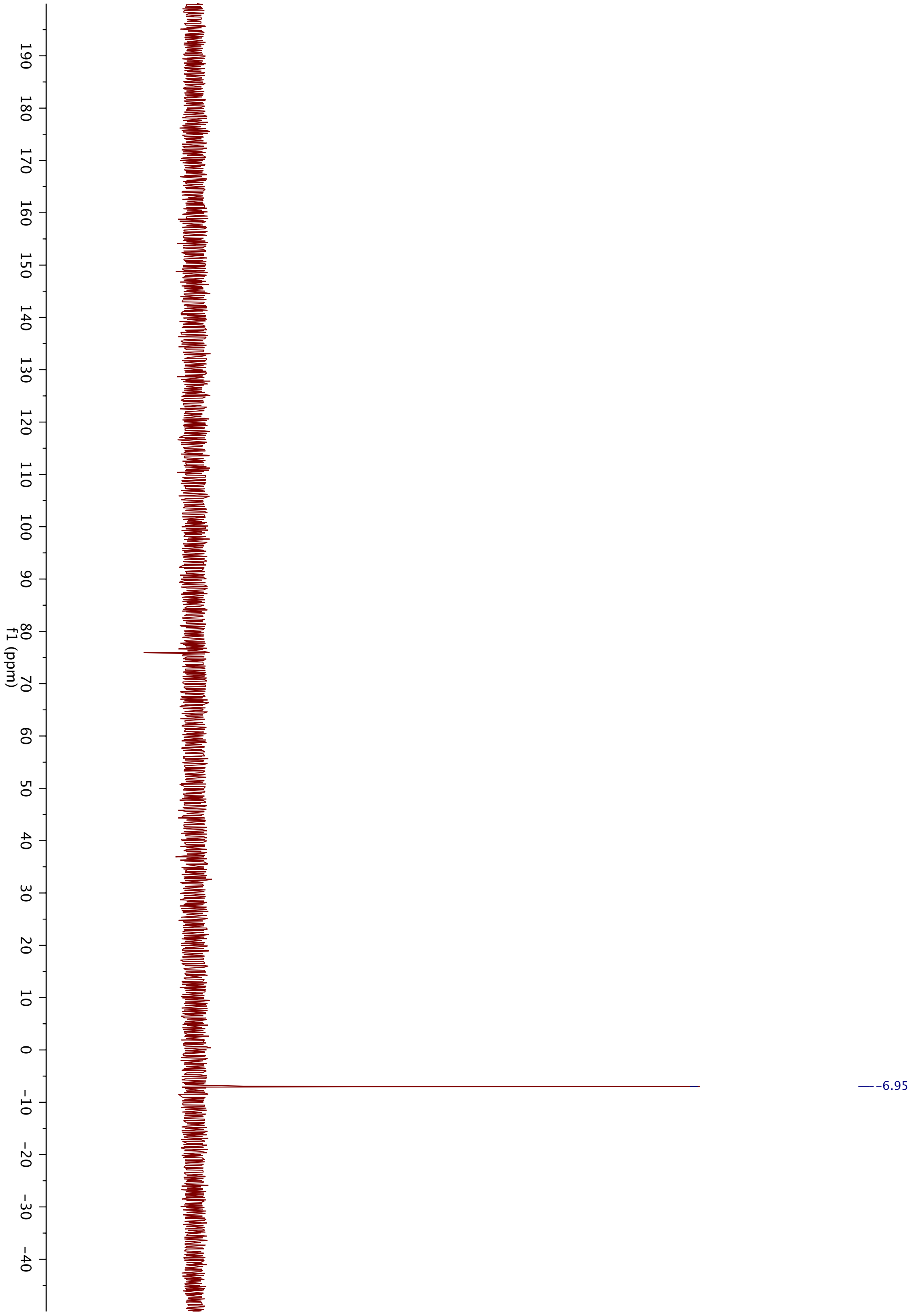

**10c**: $^1$H NMR ($CDCl_3$, 600 MHz)

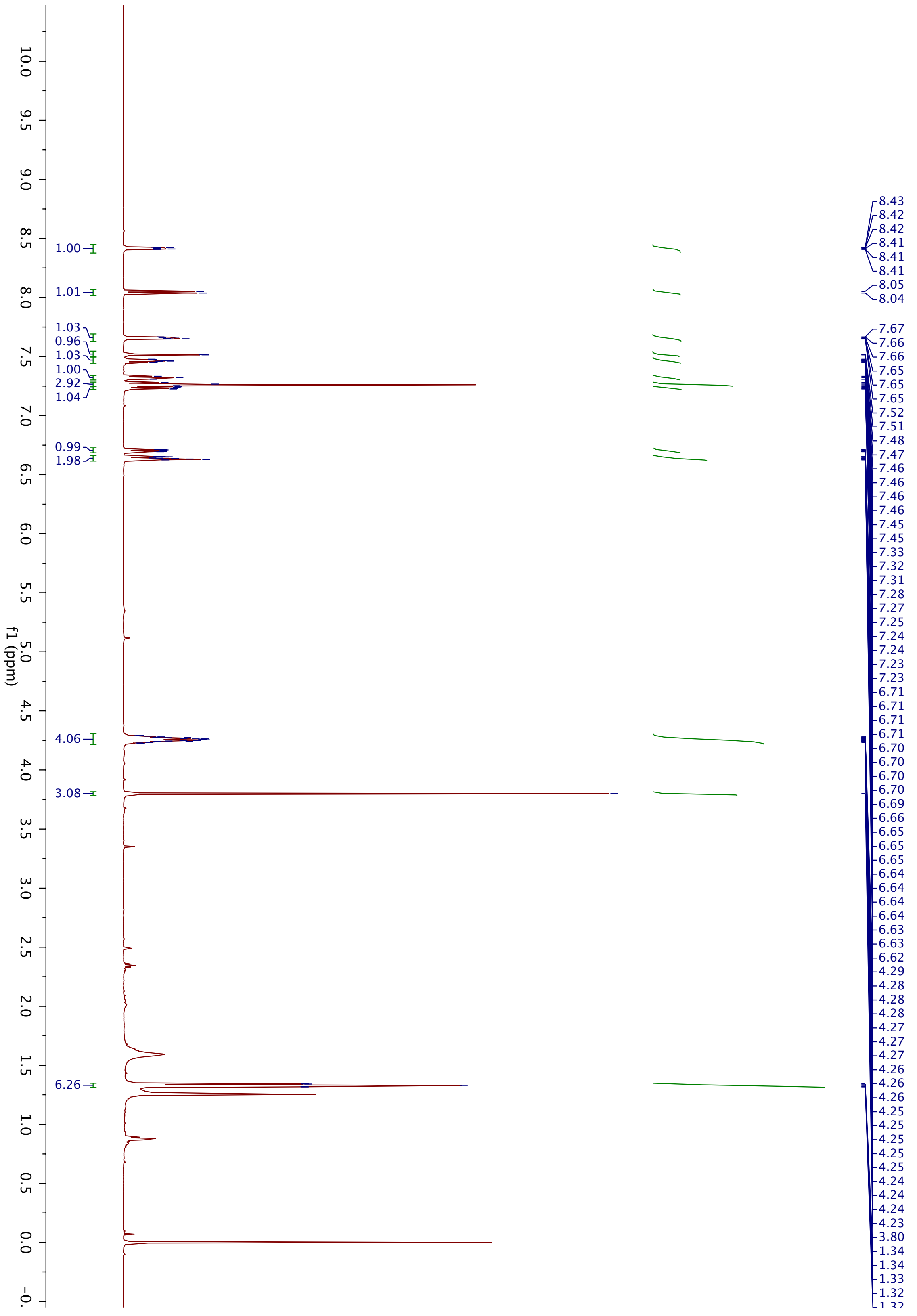

**10c**: $^{13}$C NMR ($CDCl_3$, 150 MHz)

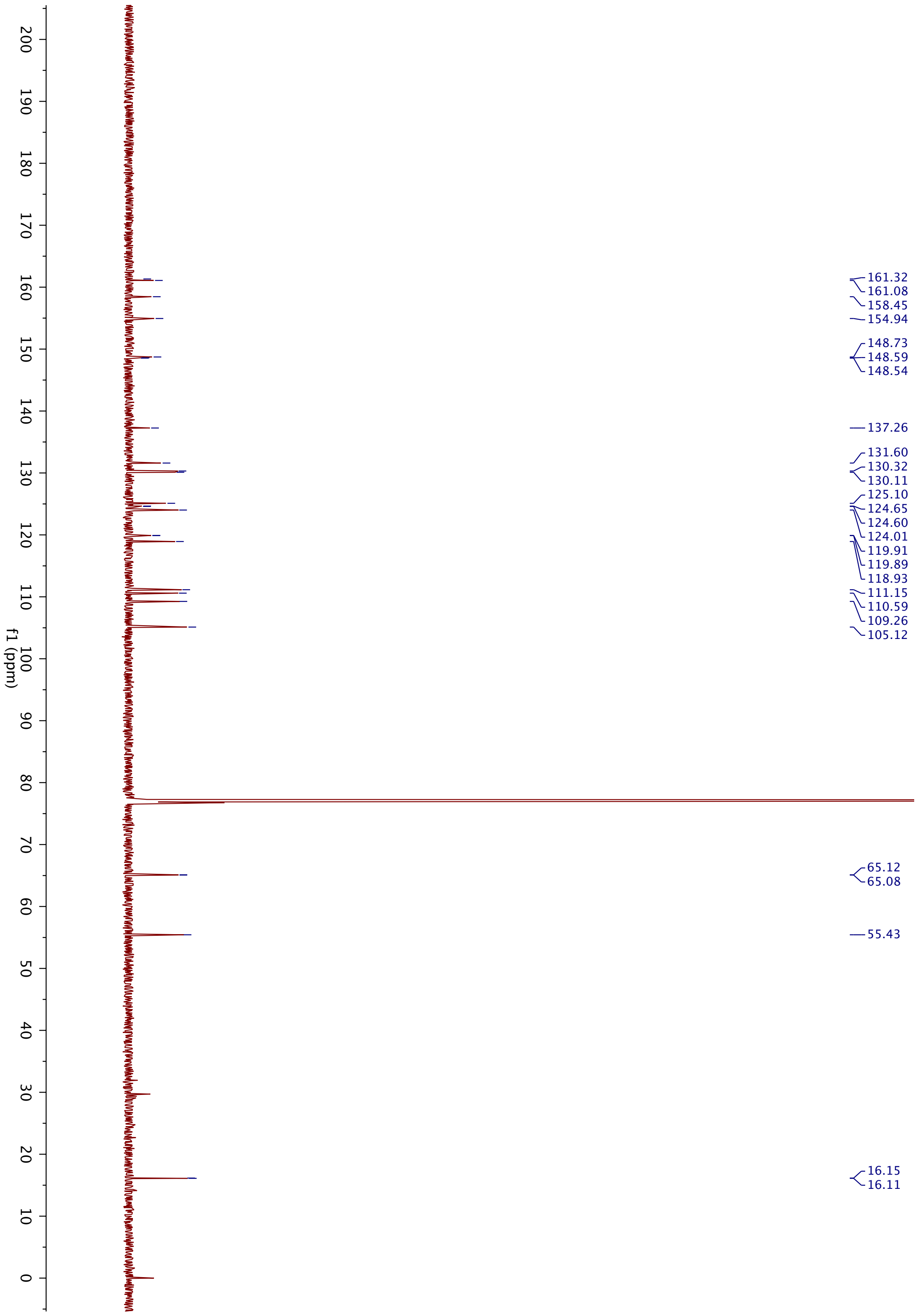

**12a**: $^{1}$H NMR ($CDCl_3$, 600 MHz)

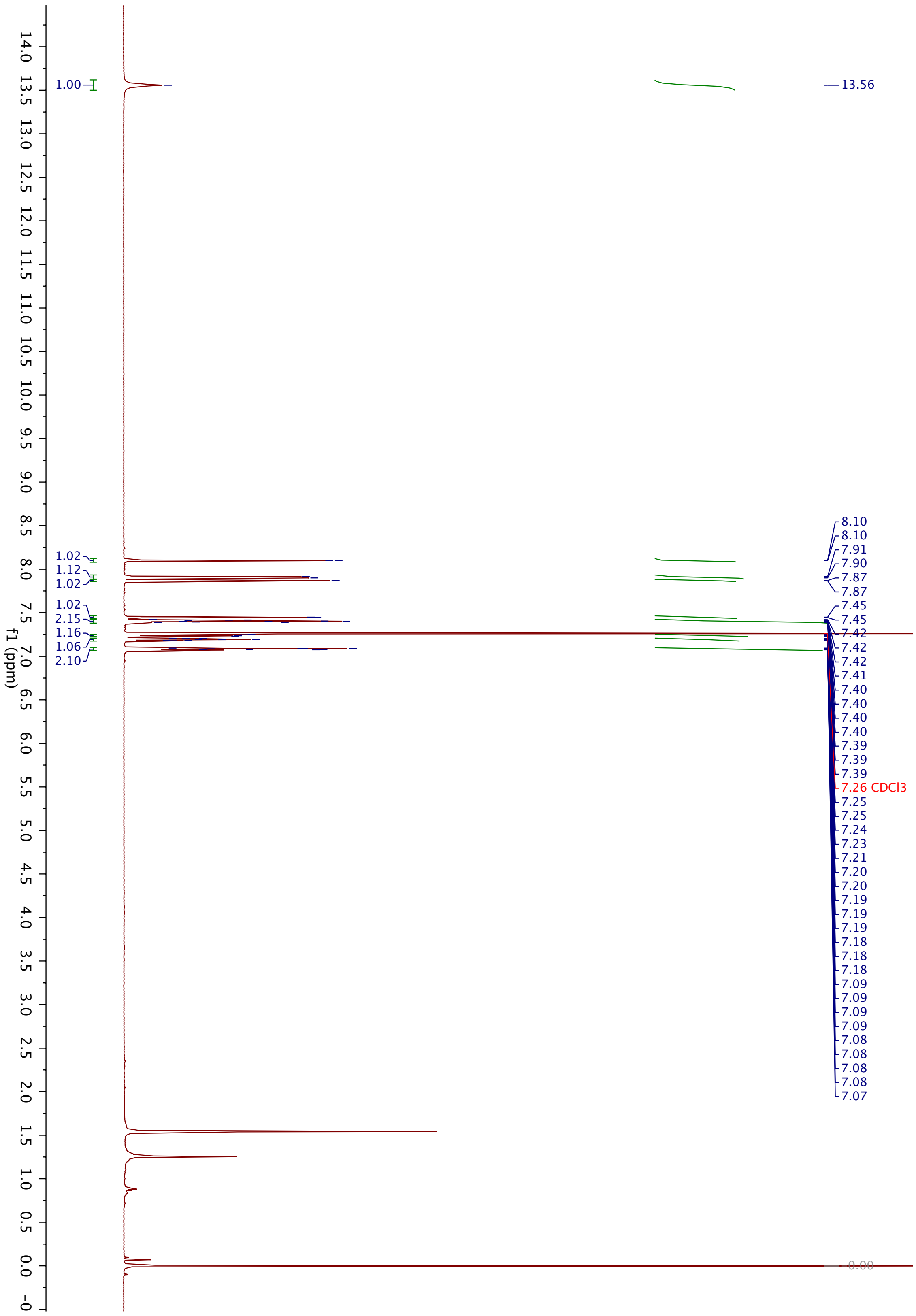

**12a**: $^{13}$C NMR ($CDCl_3$, 150 MHz)

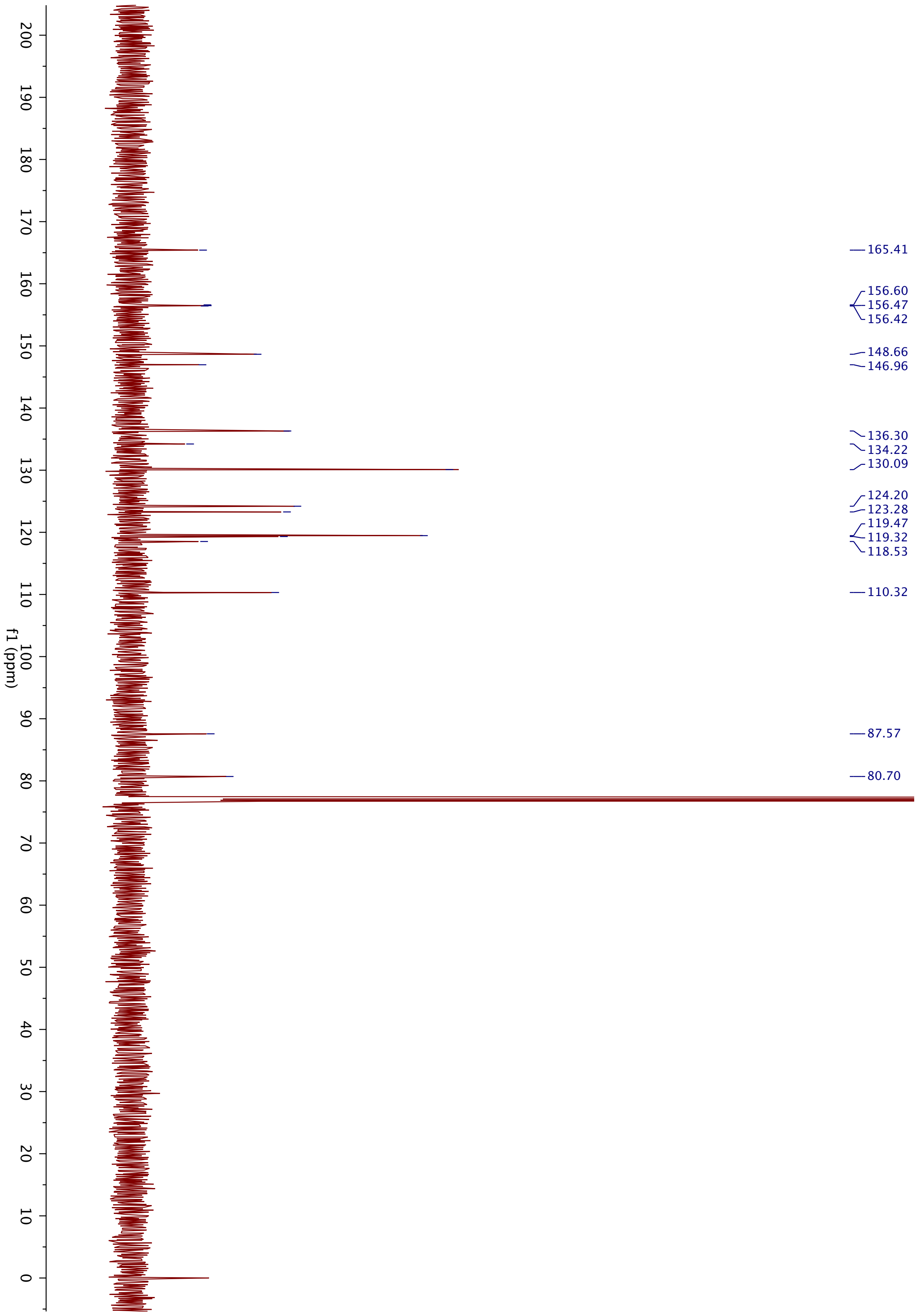

**12b**: $^1$H NMR ($CDCl_3$, 600 MHz)

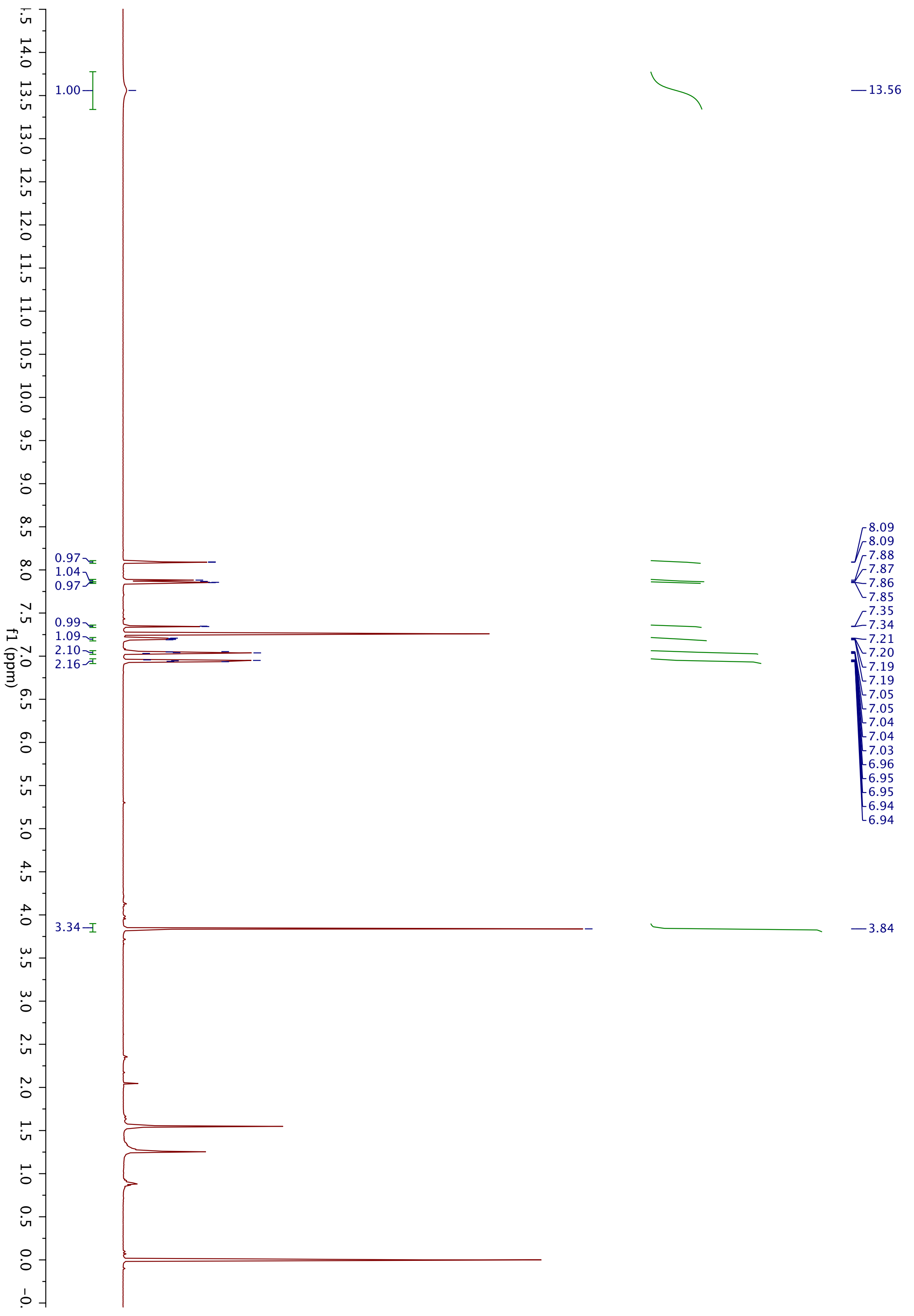

**12b**: $^{13}C$ NMR ($CDCl_3$, 150 MHz)

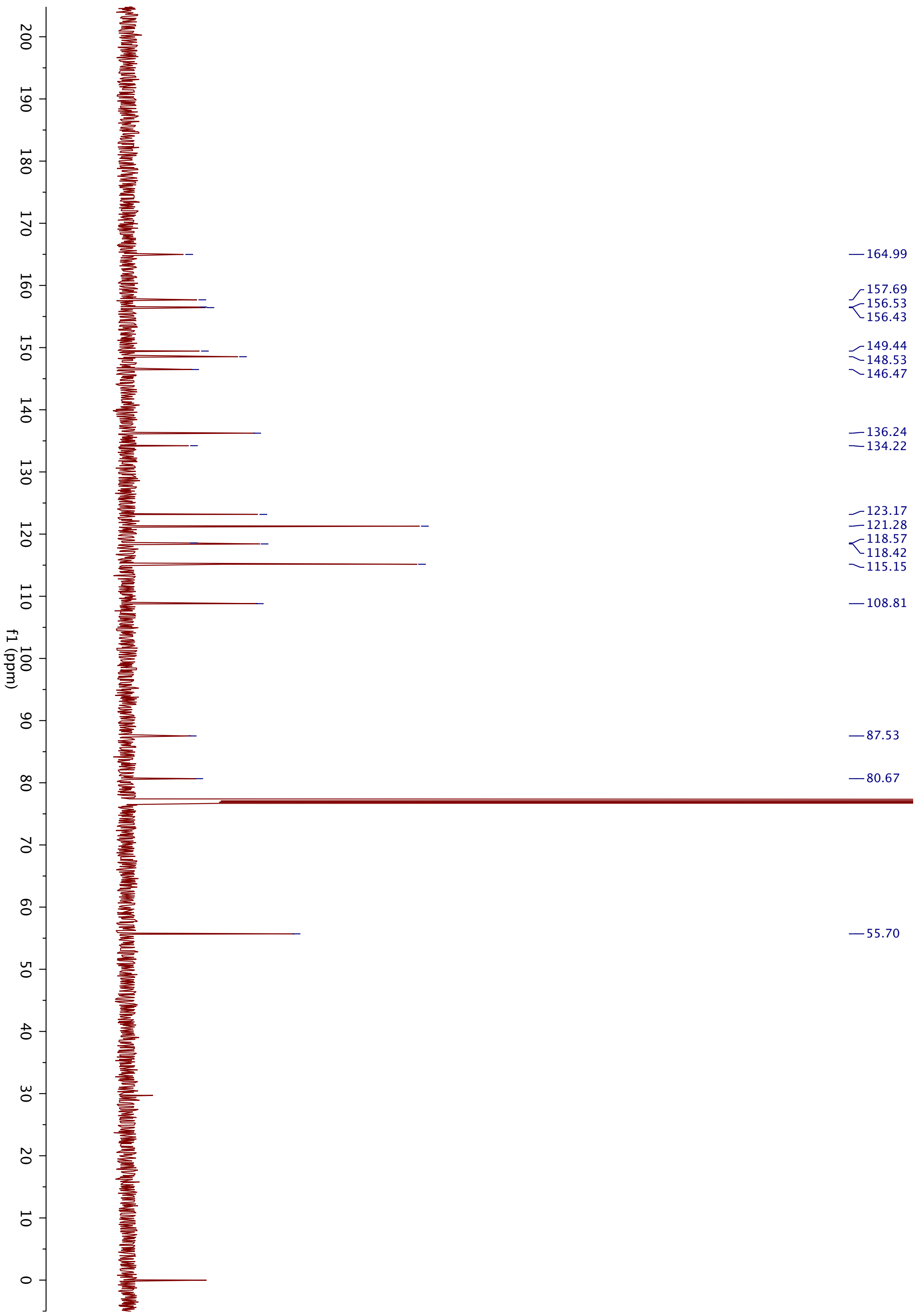

**12c**: $^{1}$H NMR ($CDCl_3$, 600 MHz)

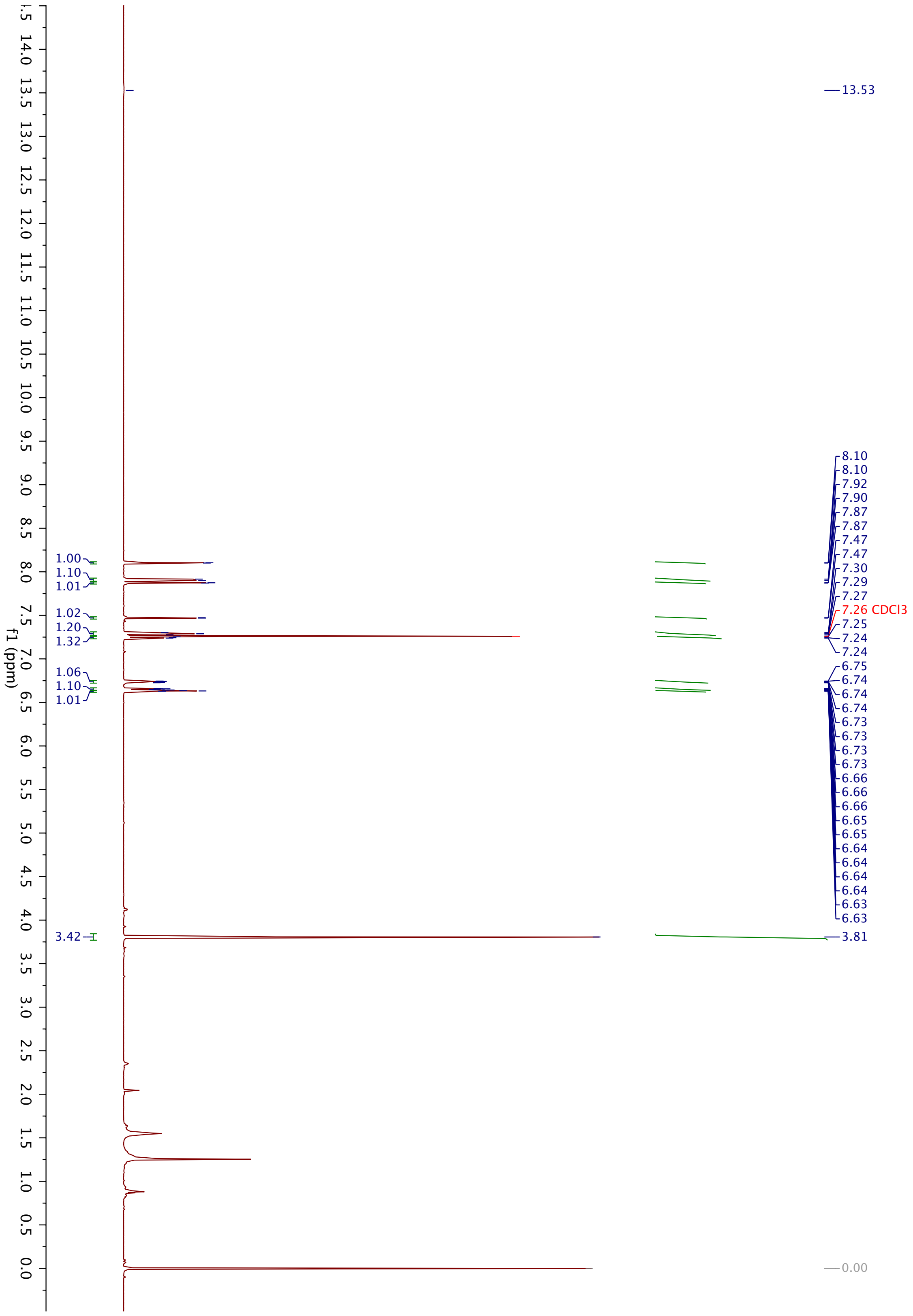

**12c**: $^{13}$C NMR ($CDCl_3$, 150 MHz)

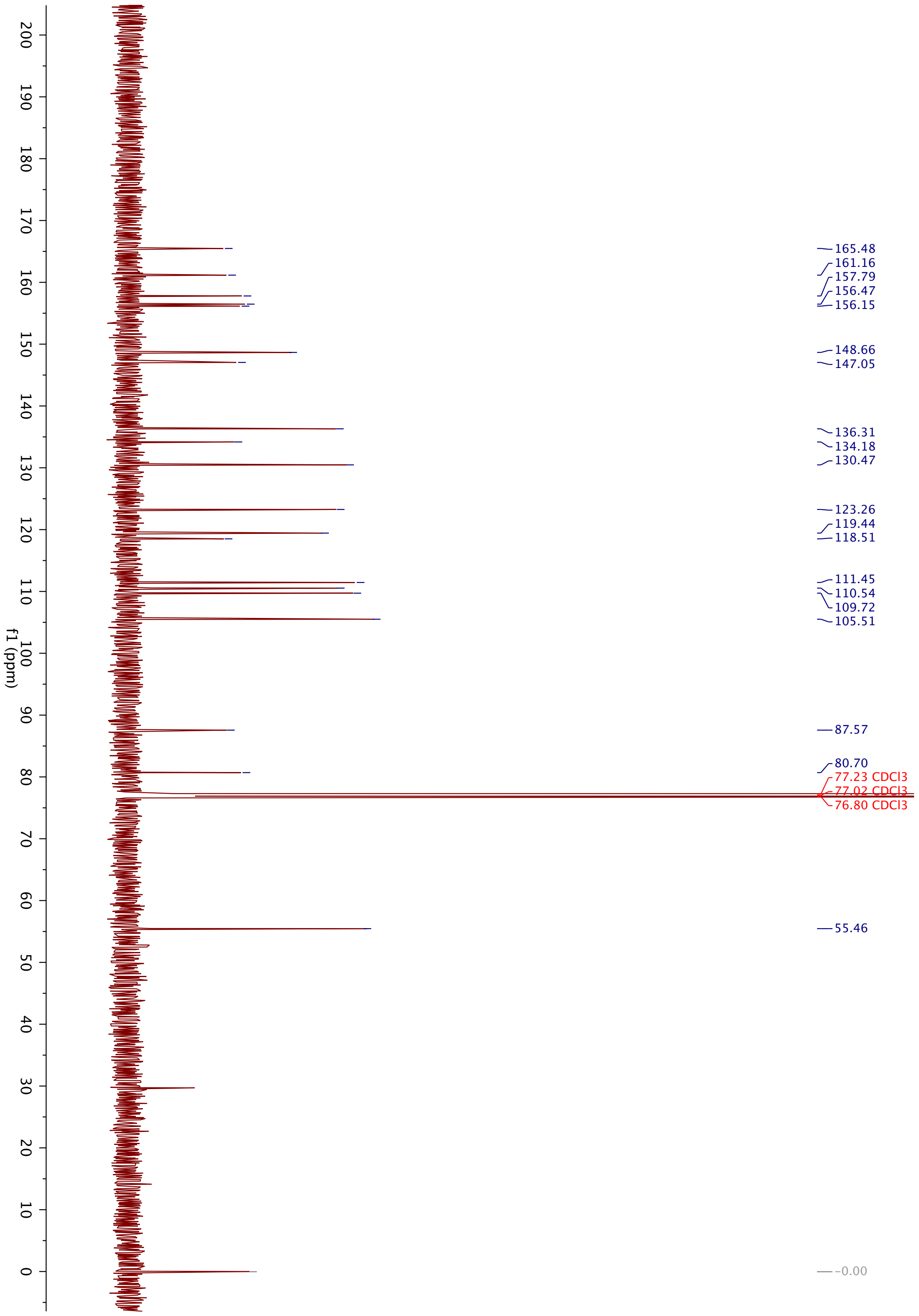

**14**: $^{1}$H NMR ($CDCl_3$, 600 MHz)

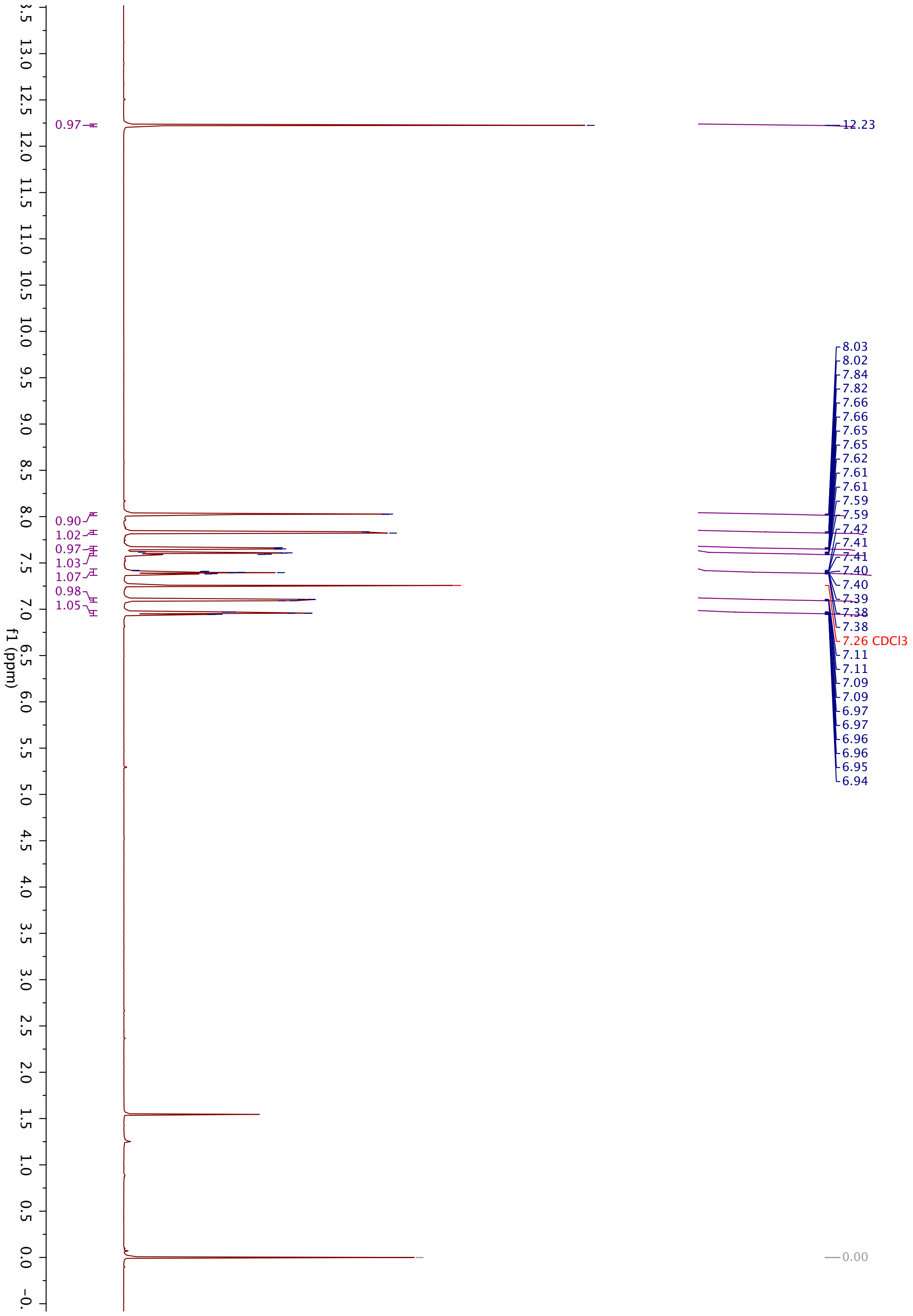

**14**: $^{13}$C NMR ($CDCl_3$, 150 MHz)

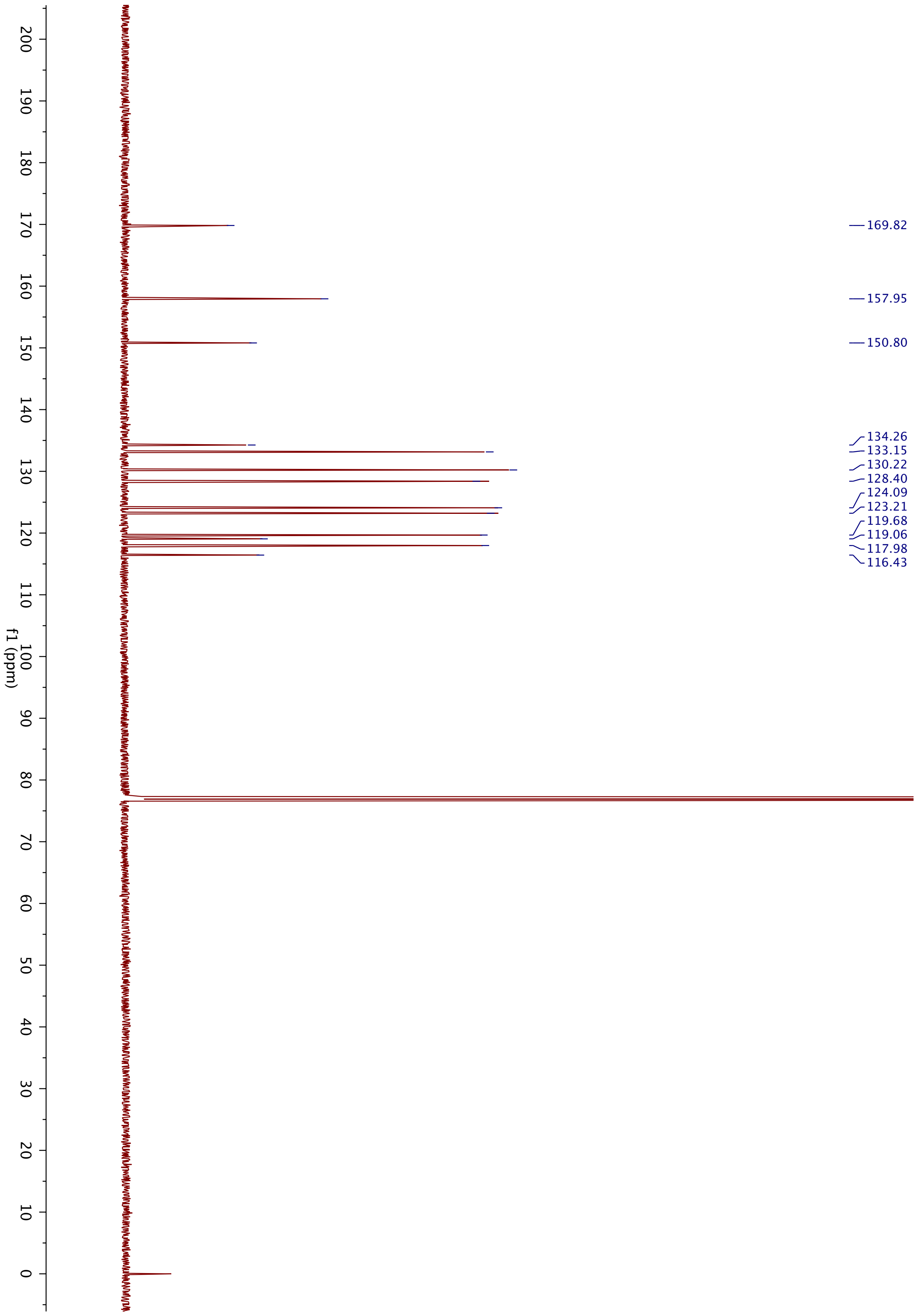

**15**: $^1$H NMR (THF-$d_8$, 500 MHz)

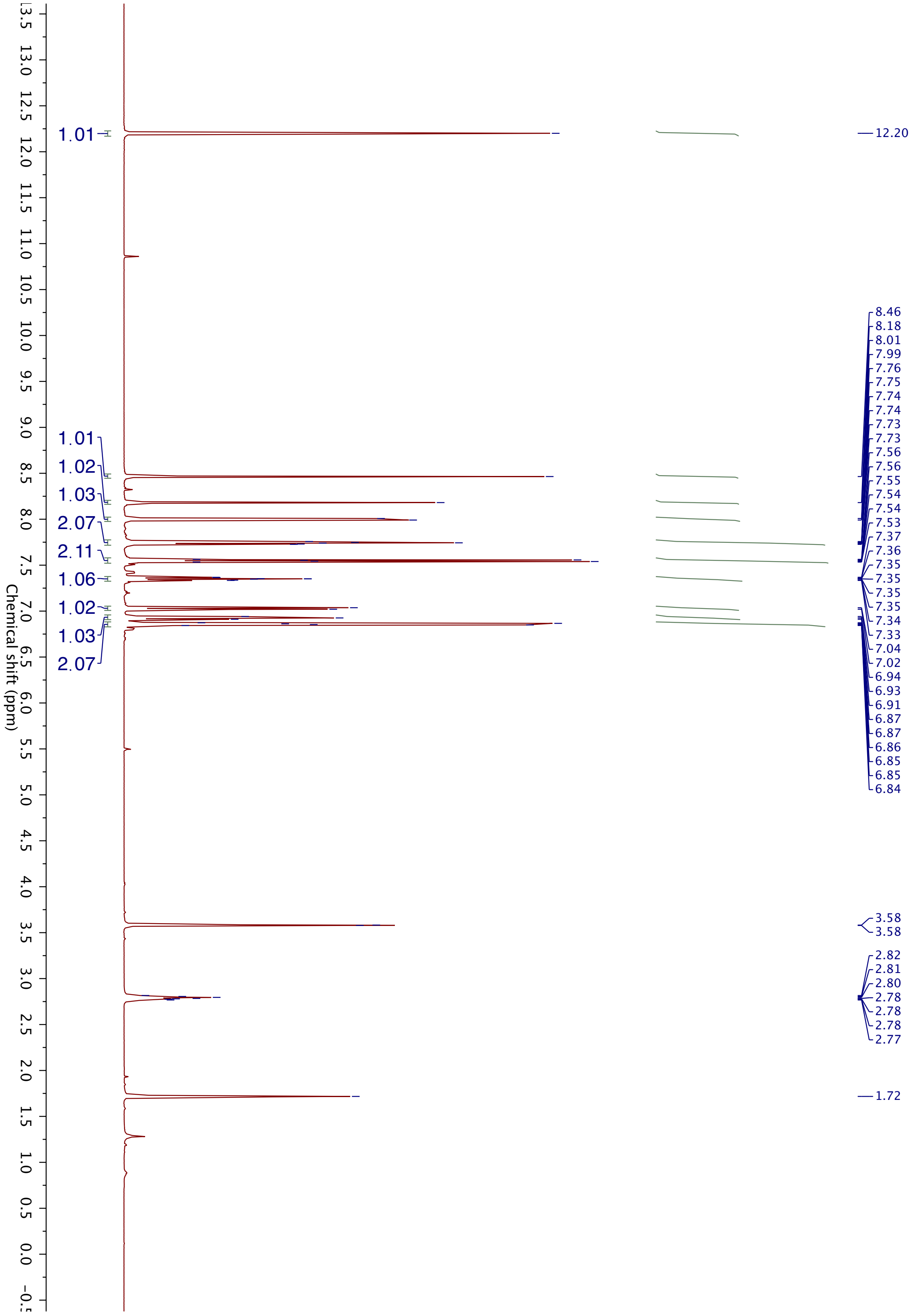

**15**: $^{13}$C NMR (THF-$d_8$, 125 MHz)

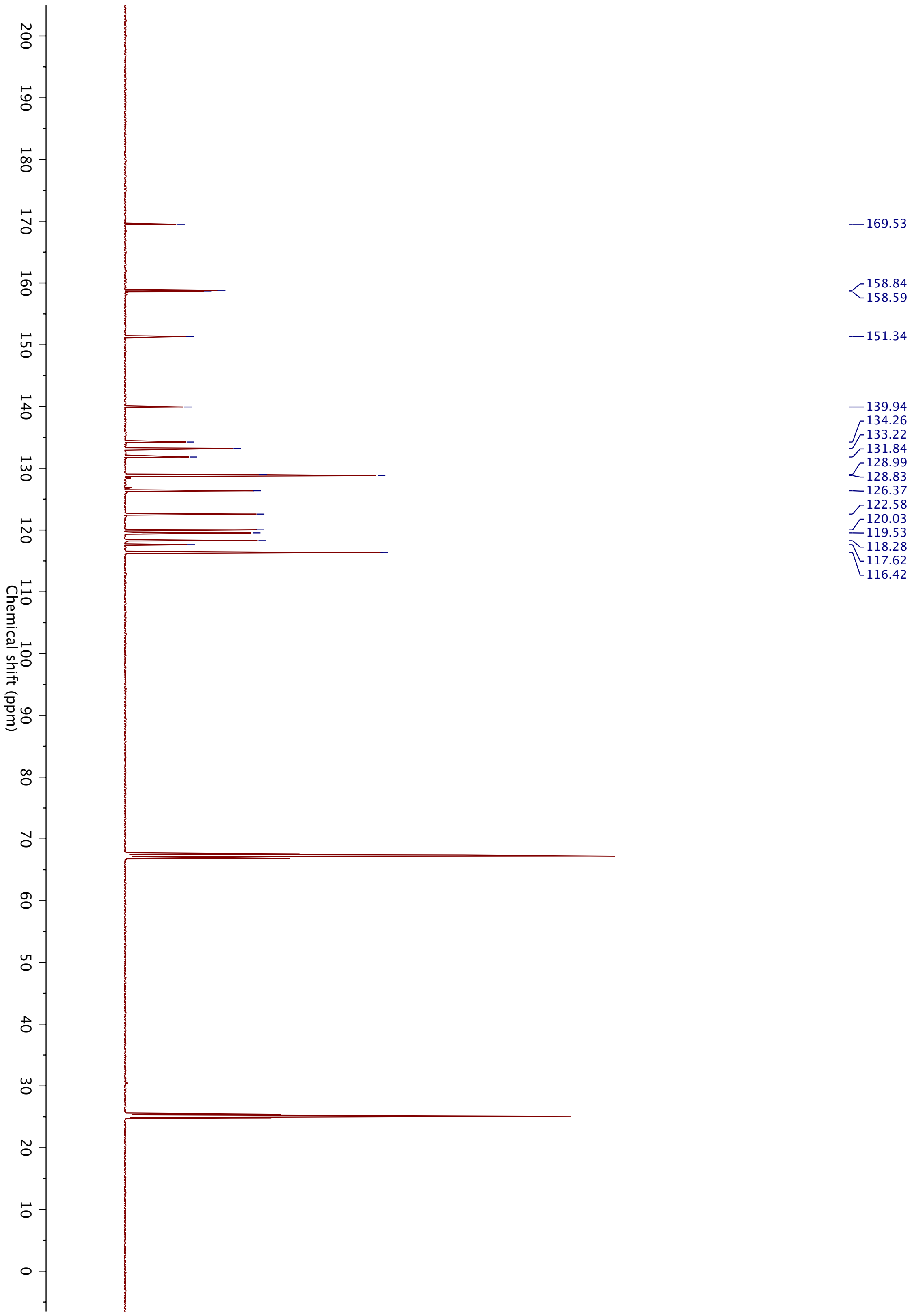

**16**: $^{1}$H NMR (THF-$d_8$, 600 MHz)

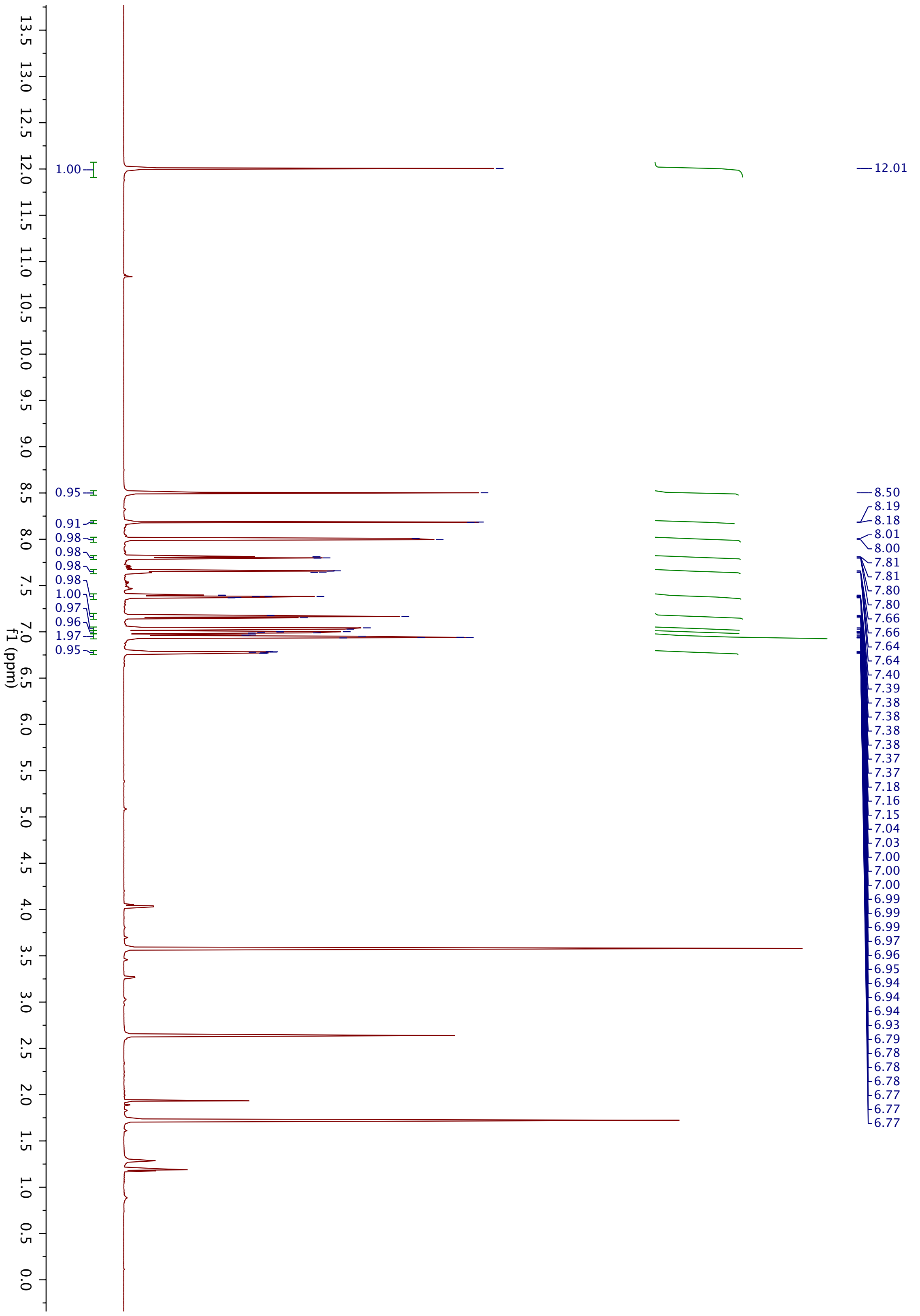

**16**: $^{13}$C NMR (THF-$d_8$, 150 MHz)

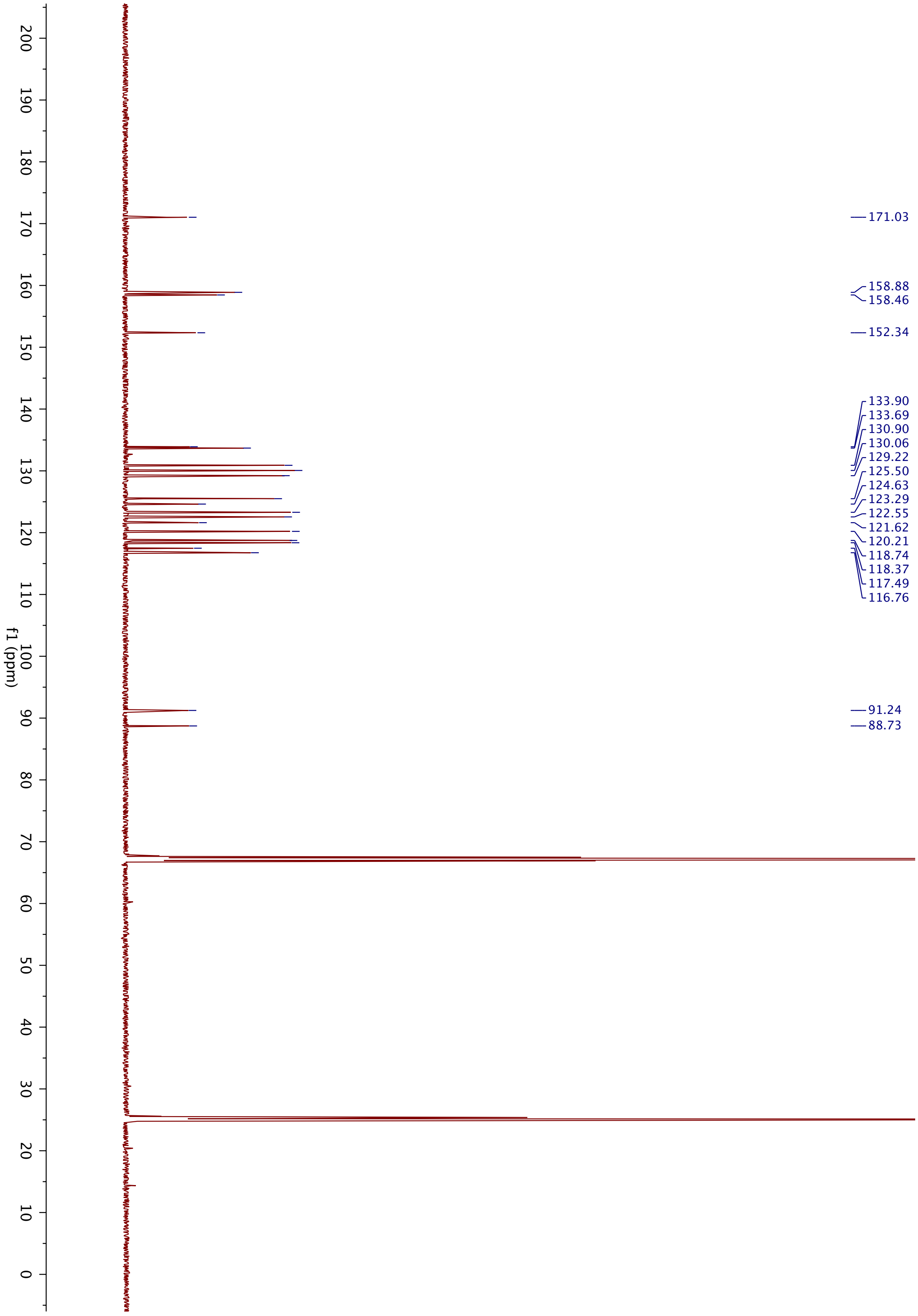

**17**: $^{31}$P NMR (CDCl3, 240 MHz)

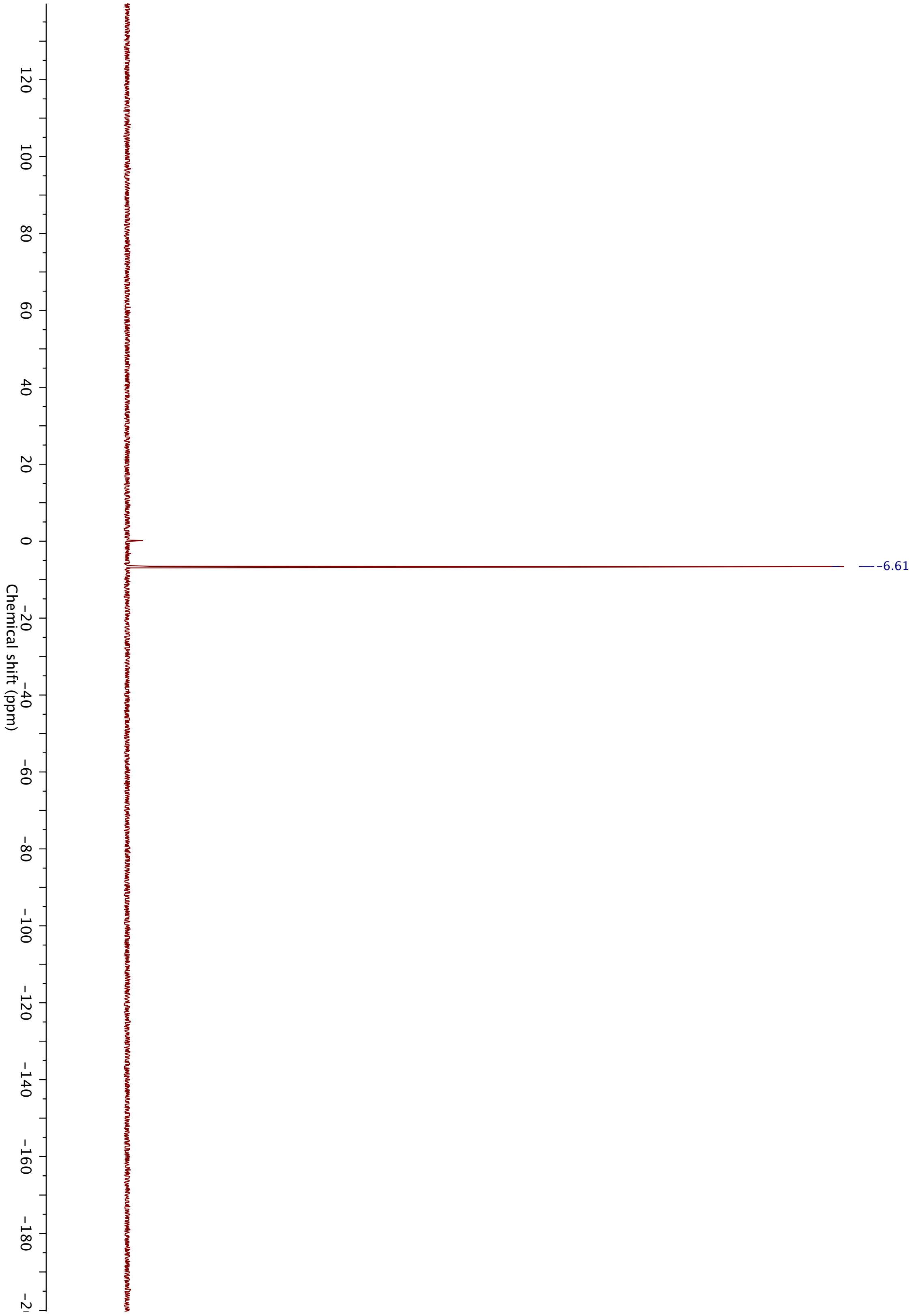

**17**: $^{1}$H NMR ($CDCl_3$, 600 MHz)

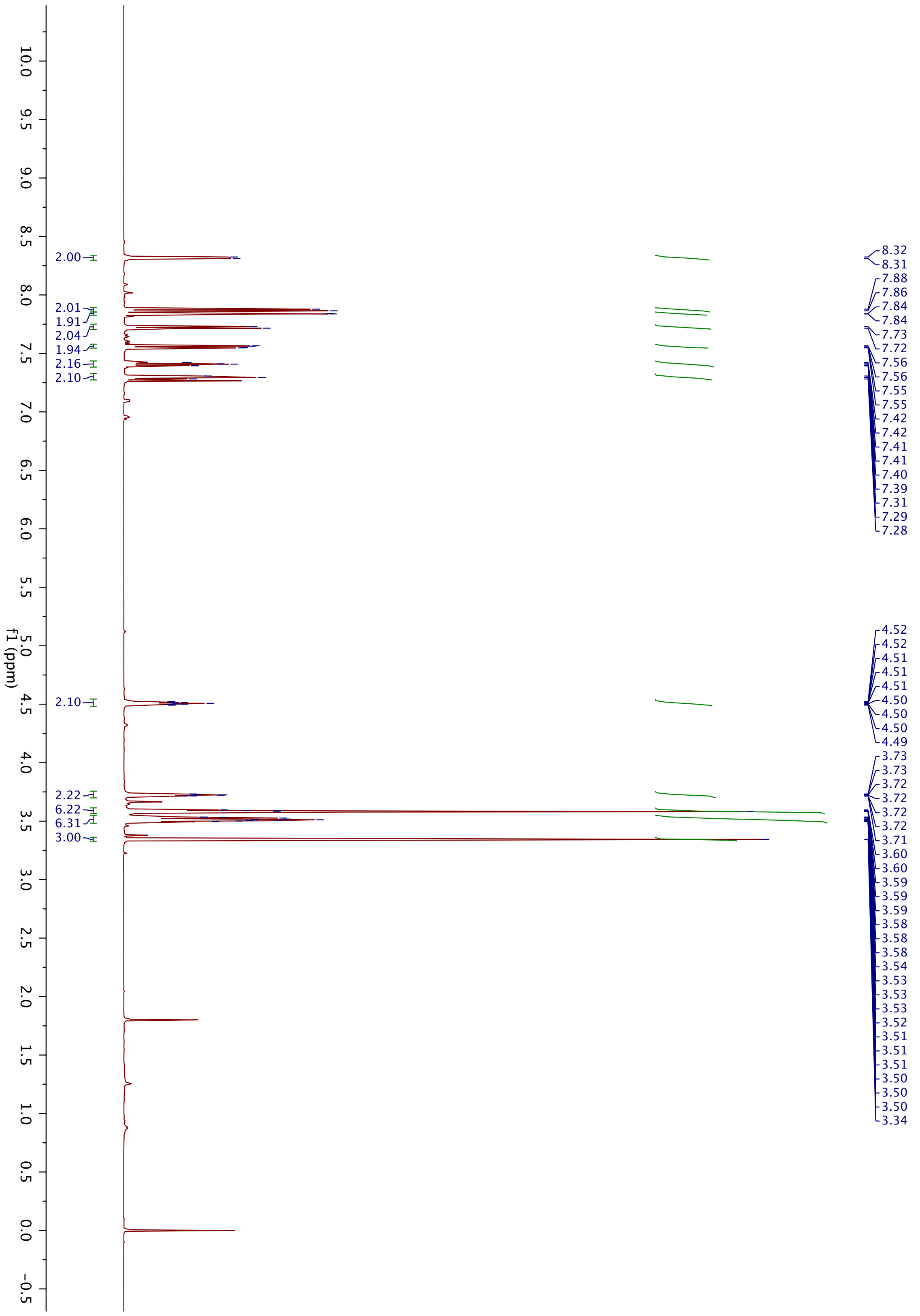

**17**: $^{13}$C NMR ($CDCl_3$, 150 MHz)

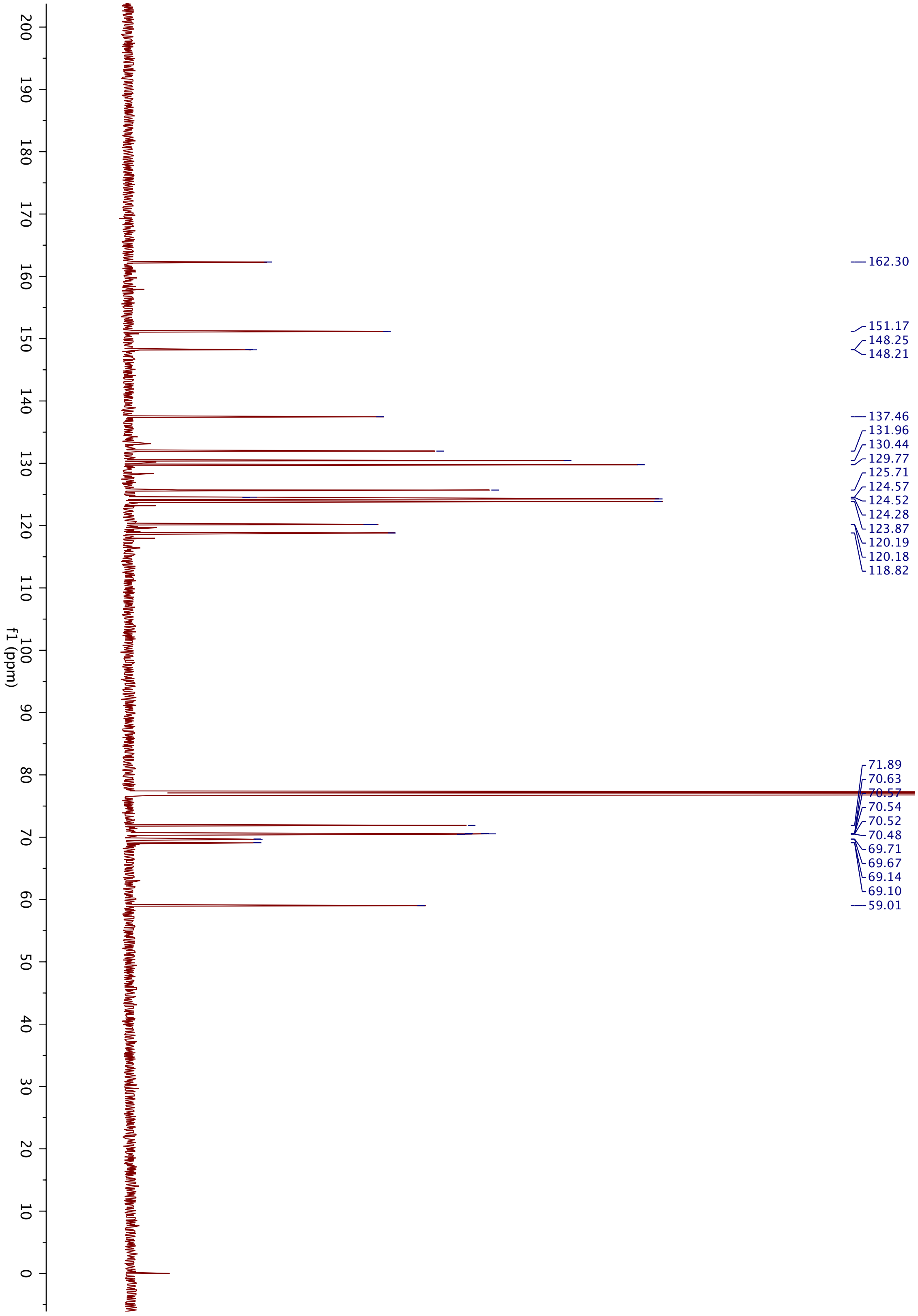

**18**: $^{31}$P NMR (CDCl3, 200 MHz)

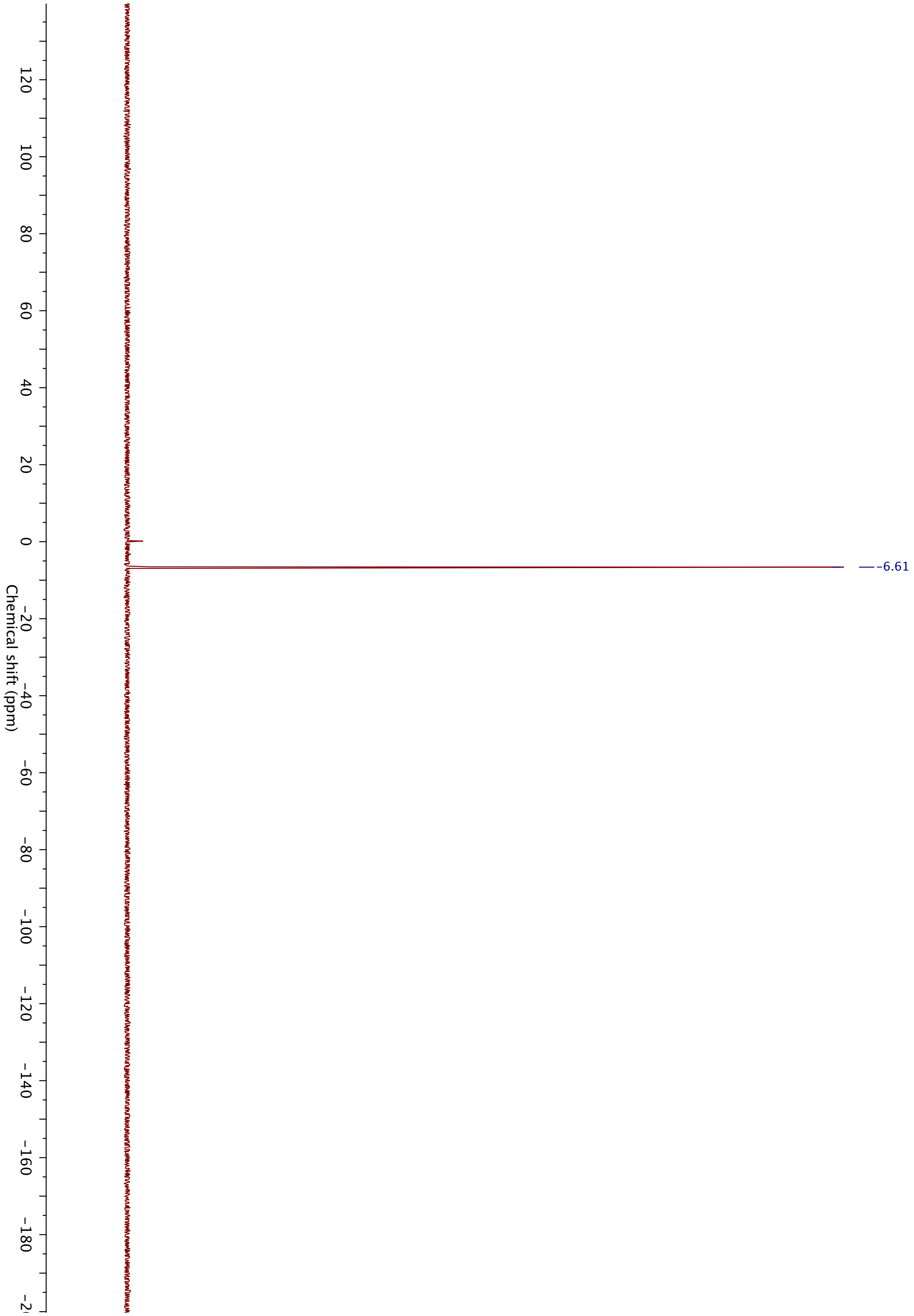

**18**: $^{1}$H NMR ($CDCl_3$, 600 MHz)

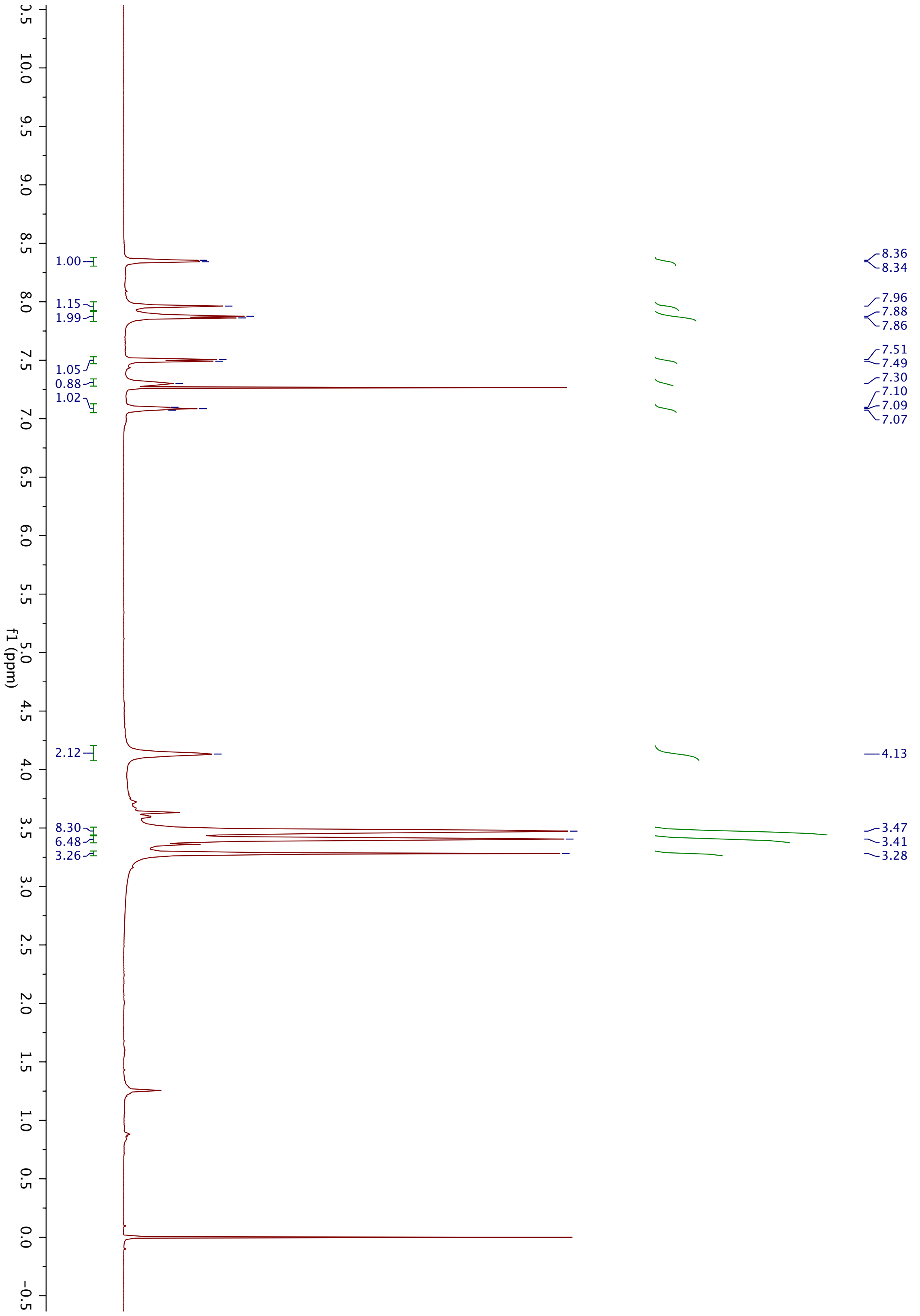

**18**: $^{13}$C NMR ($CDCl_3$, 150 MHz)

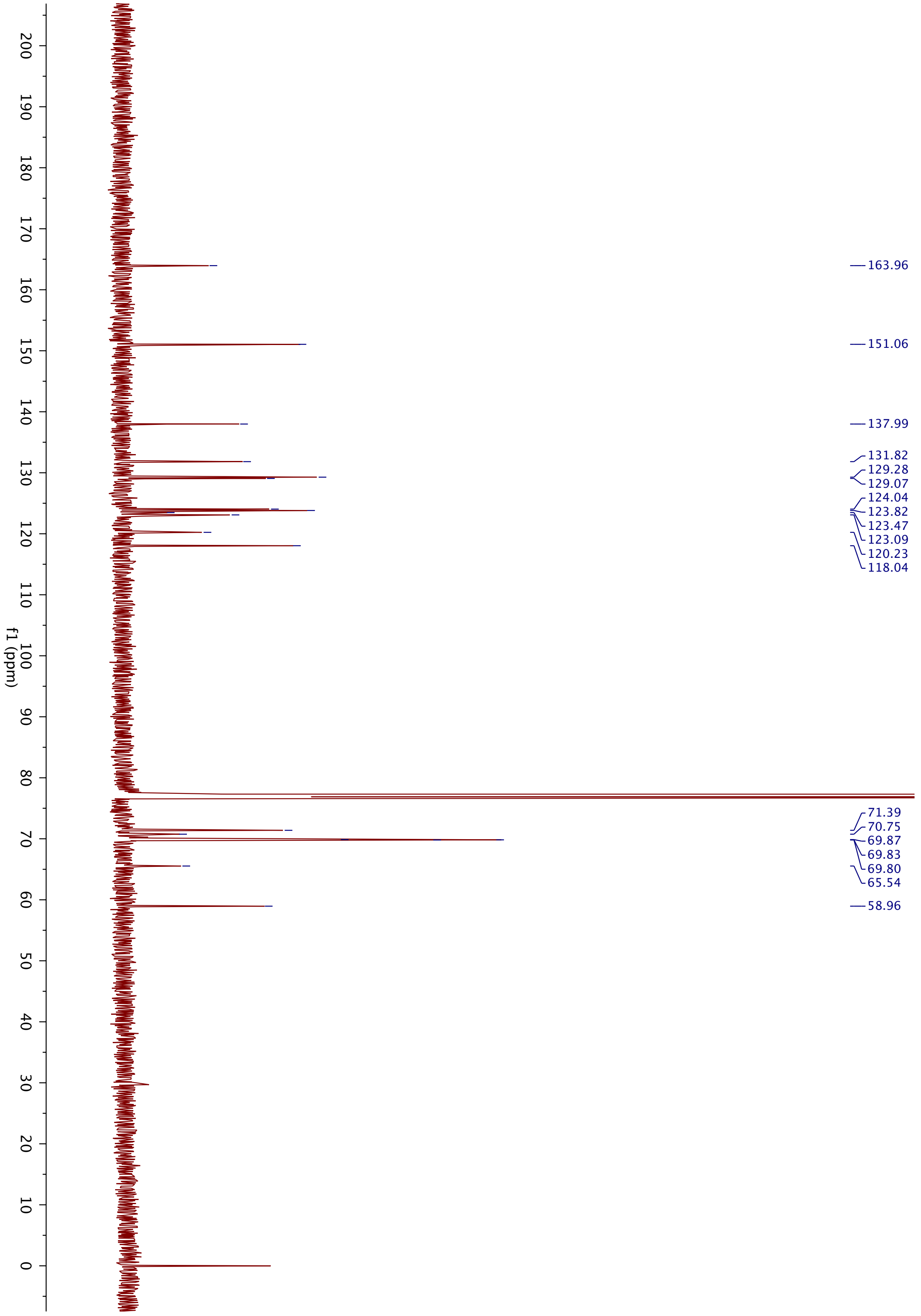

**19**: $^{31}$P NMR ($CD_3OD$, 200 MHz)

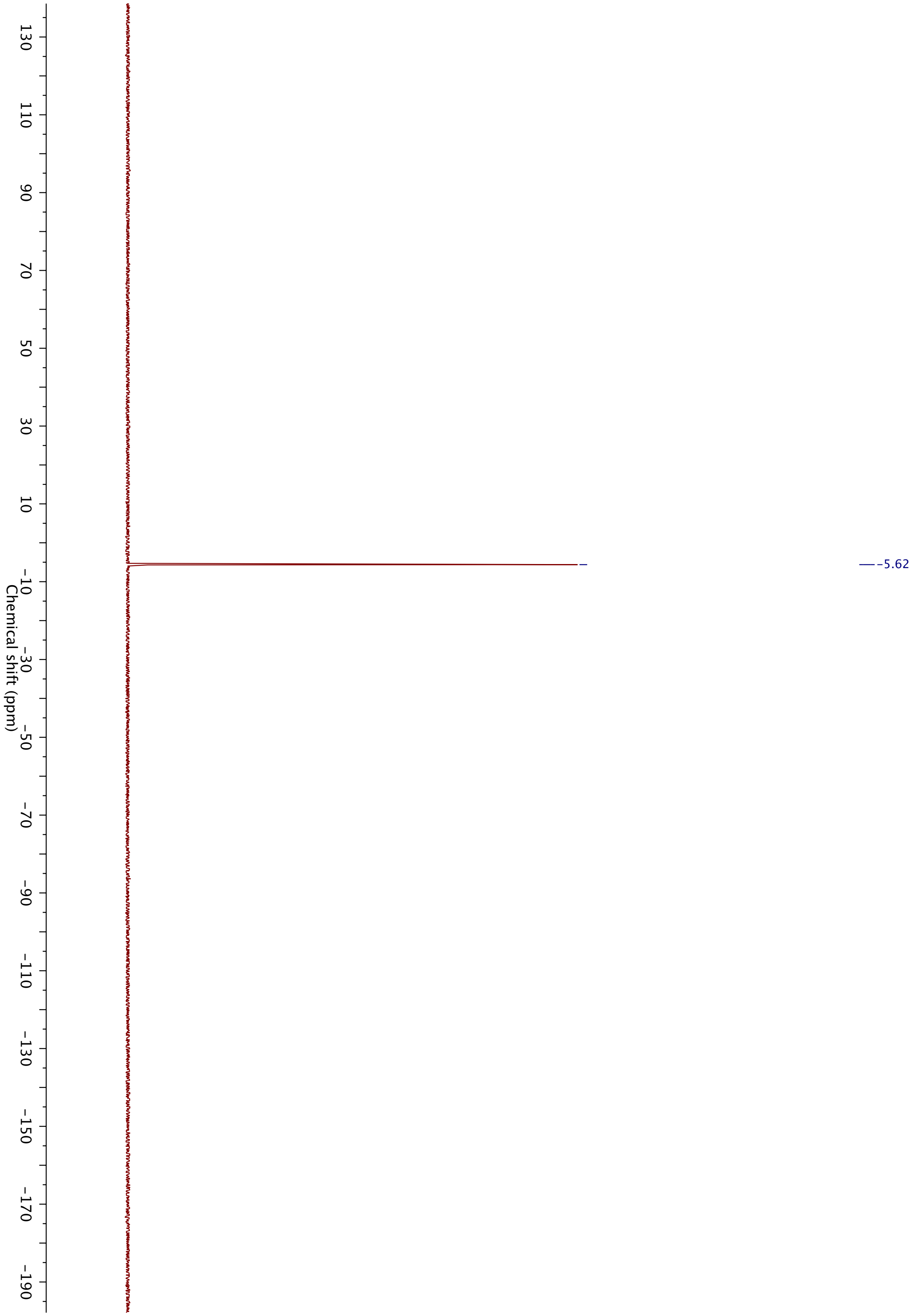

**19**: $^{1}$H NMR ($CD_3OD$, 500 MHz)

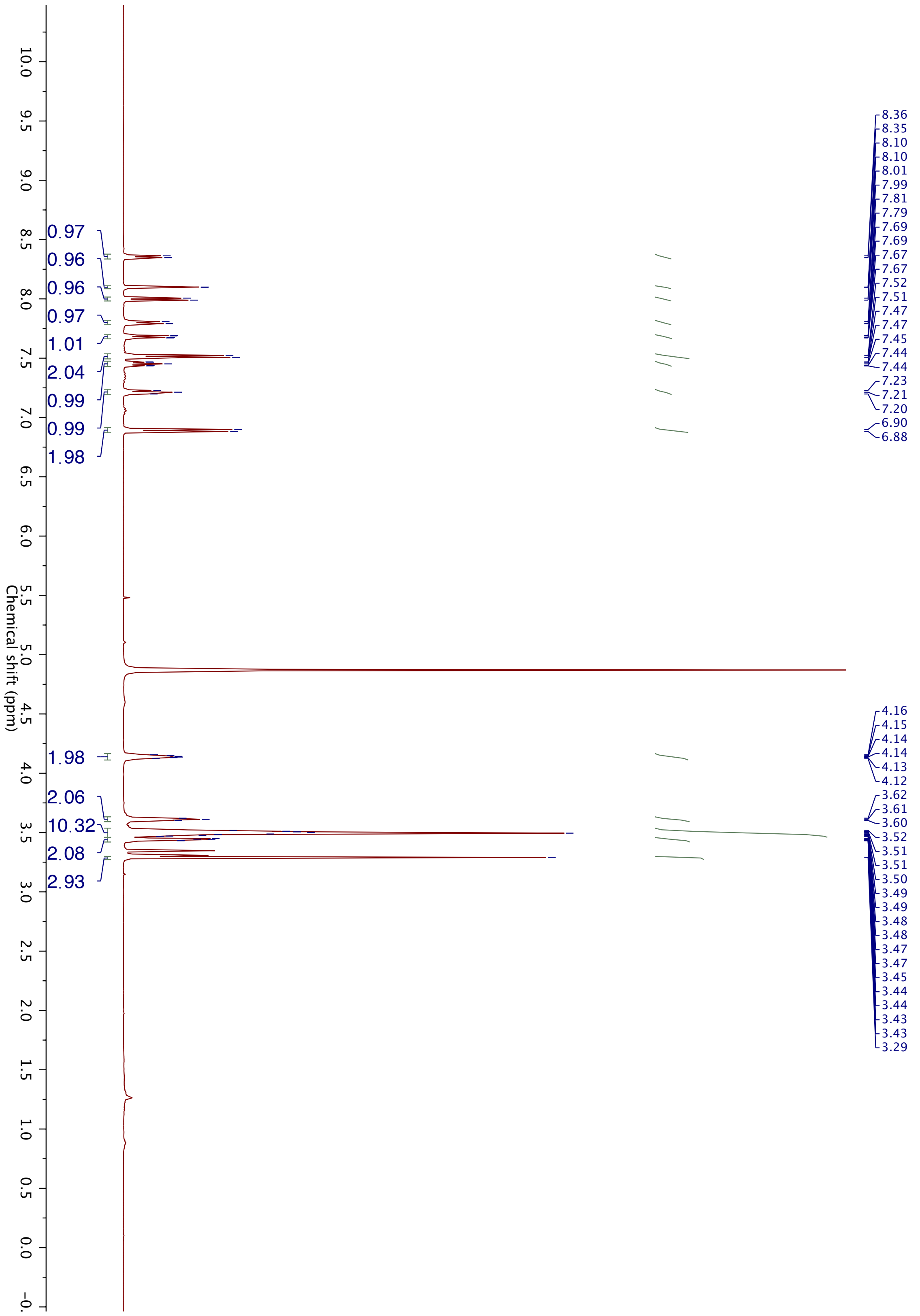

**19**: $^{13}$C NMR ($CD_3OD$, 125 MHz)

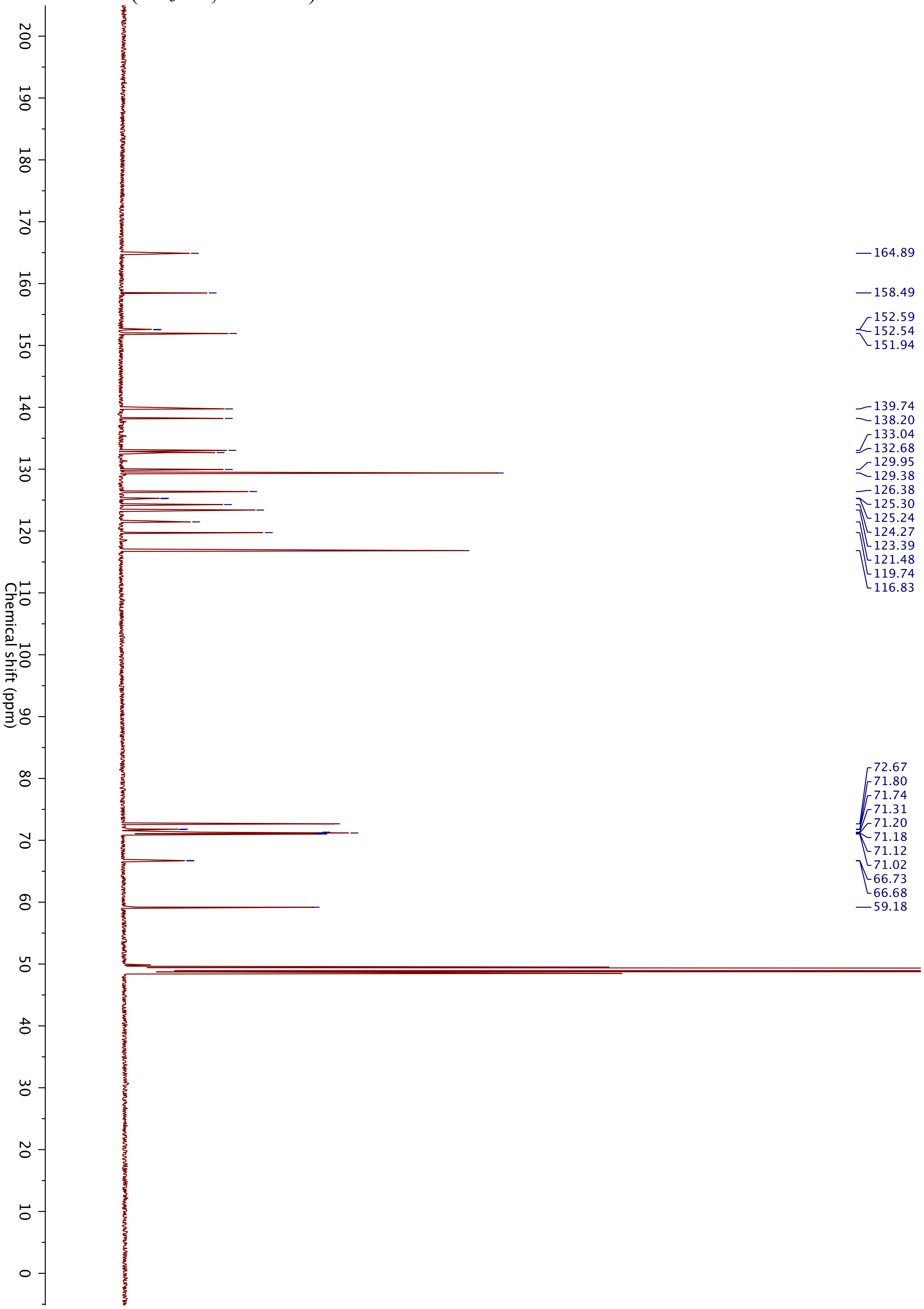

**19-I$_2$**: $^{31}$P NMR ($CD_3OD$, 200 MHz)

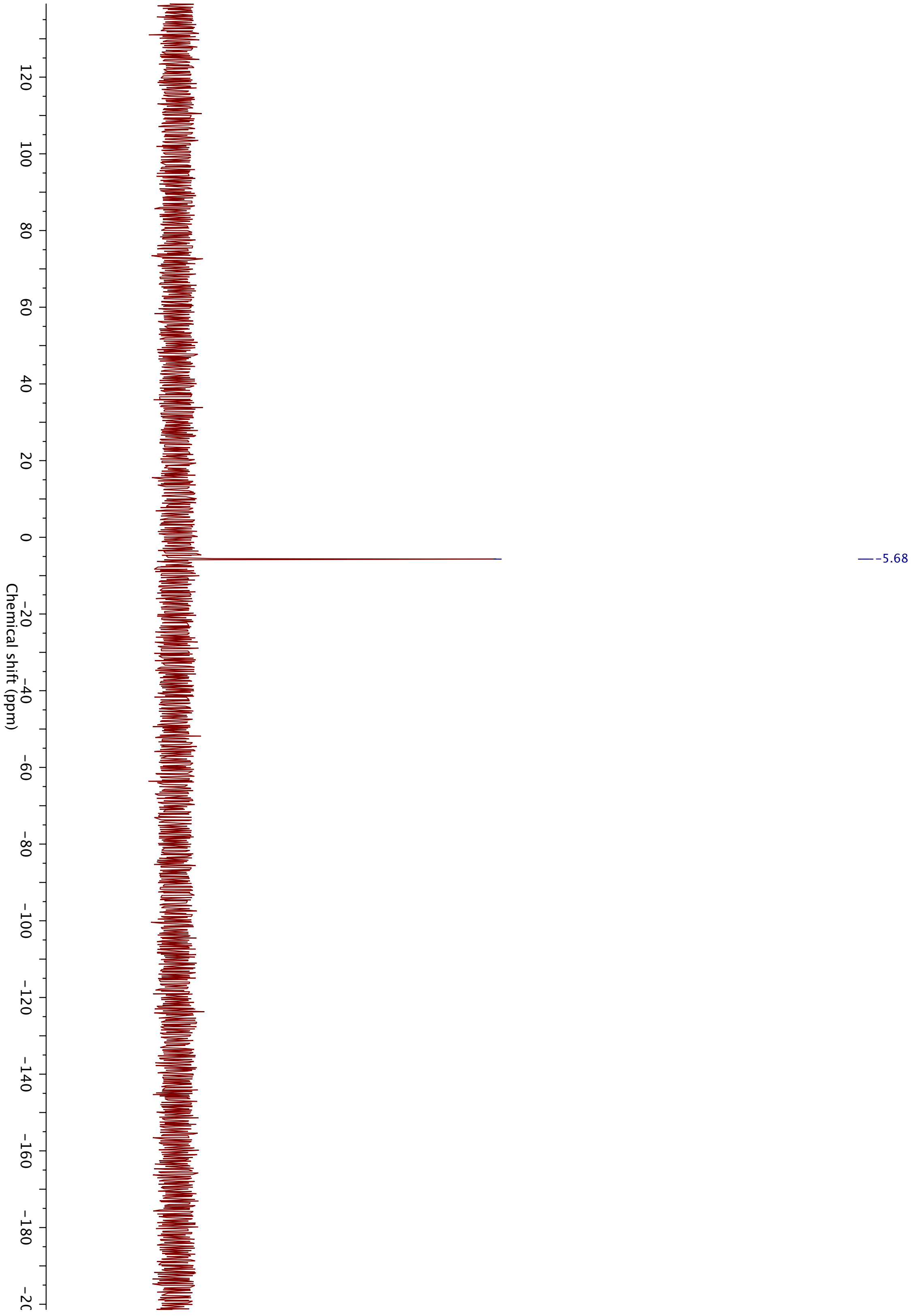

**19-I$_2$**: $^1$H NMR ($CD_3OD$, 600 MHz)

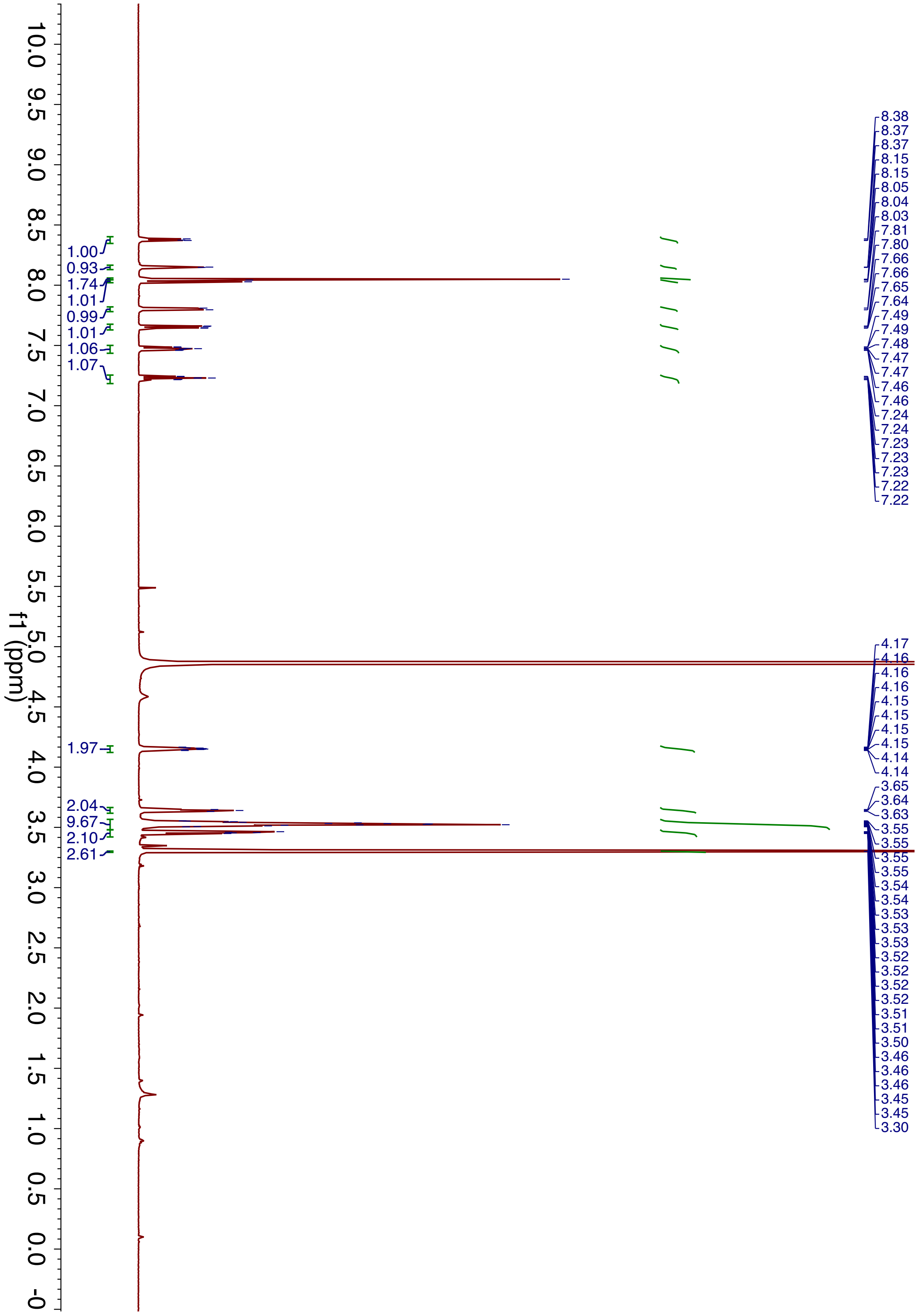

**19-I₂**: $^{13}$C NMR ($CD_3OD$, 150 MHz)

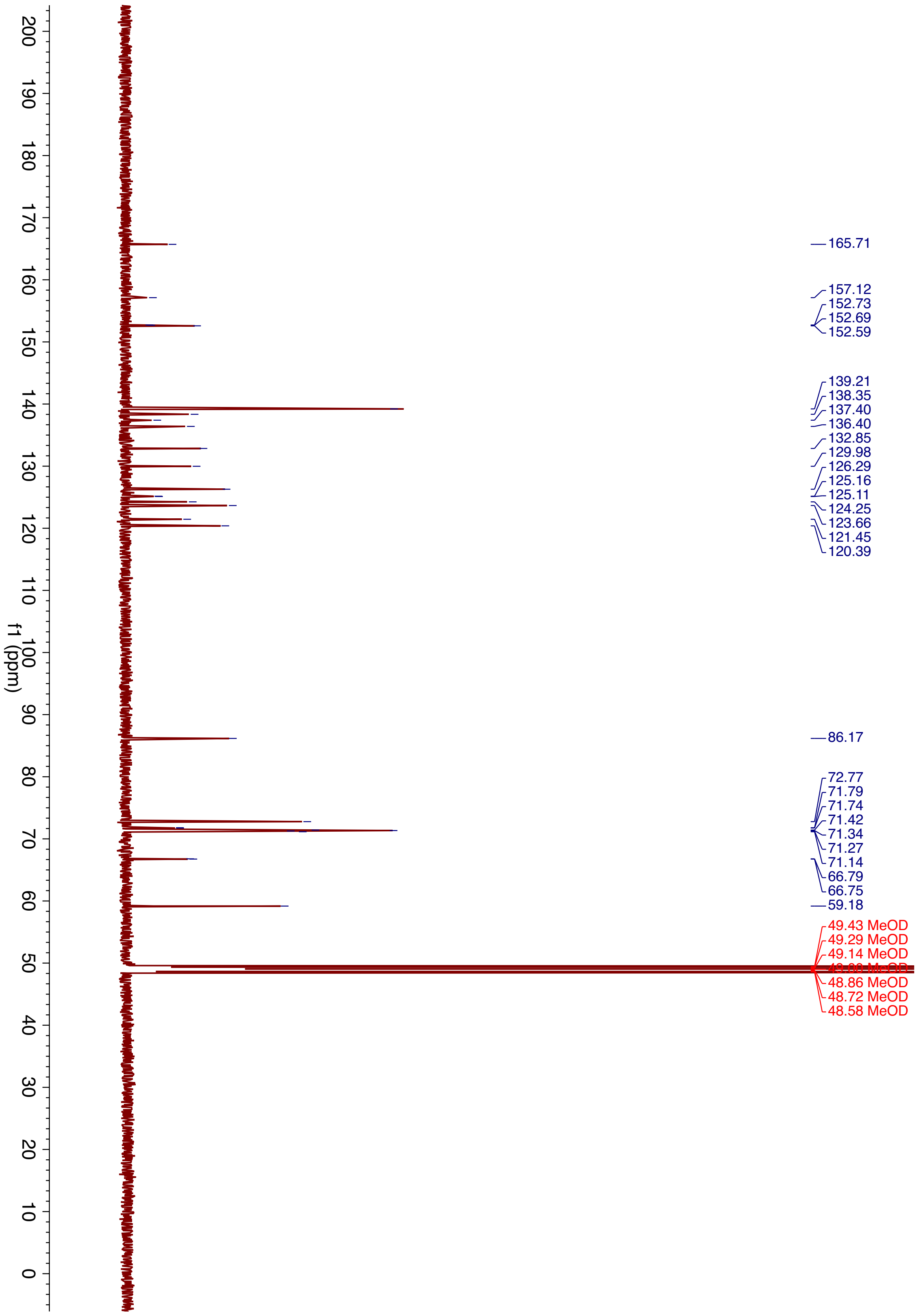

Supplement: Supplementary file 1 [file molecules-27-08682-s001.zip › molecules-2036077-supplementary.pdf]
